# Host-transposable element coexistence: a matter of resistance, tolerance and trade-off

**DOI:** 10.1038/s44318-026-00795-z

**Published:** 2026-05-13

**Authors:** Séverine Chambeyron, Alain Pélisson, Yuica Koga, Charlotte Grimaud, Mikiko C Siomi

**Affiliations:** 1 Institute of Human Genetics (IGH), Univ Montpellier, CNRS, Montpellier, France; 2 Department of Biological Sciences, Graduate School of Science, The University of Tokyo, Tokyo, Japan

**Keywords:** Evolution & Ecology, RNA Biology

## Abstract

Transposable elements (TEs) are ubiquitous, mobile DNA elements that often exist as multiple copies within host genomes. To persist despite ongoing mutational decay, these genomic parasites must continuously generate new insertions into the germline genome, a process that risks compromising host reproductive capacity by disrupting coding regions and regulatory sequences and by reshaping chromosomal architecture. Accordingly, hosts have evolved mechanisms to repress TE activity and reduce its fitness costs. In principle, however, such antagonism could lead to lineage-specific TE extinction, which may be suboptimal for hosts, as TEs possess aspects that confer beneficial functions to them. Therefore, hosts not only resist but also tolerate and even exploit the presence of TEs. In parallel, TEs themselves have acquired and deployed unique strategies that promote their own persistence while limiting harm to the host. This review explores how “peaceful” coexistence between hosts and TEs is achieved, focusing on resistance, tolerance, and the associated trade-offs from both host and TE perspectives.

## Abstract (web summary)

Host genomes balance resistance, tolerance, and exploitation of transposable elements, while transposons evolve strategies to persist with minimal fitness costs, shaping a dynamic coexistence.

## Graphical abstract

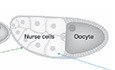

## Introduction

Transposable elements (TEs) are mobile genetic elements that parasitize a substantial portion of nearly all studied genomes (Wells and Feschotte, 2020) (Table 1). Like their hosts, TEs encode genes whose ultimate goal is to ensure their own survival and propagation across generations. To this end, new TE copies become inserted elsewhere in the host genome, which frequently generates a wide spectrum of host–genome mutations, including insertions, duplications, translocations, deletions, and inversions, with the potential to disrupt gene expression and function (Payer and Burns, 2019) and even to remodel chromosomal architecture (Choudhary et al, 2023; Haws et al, 2022; Li and Shen, 2023).

**Table 1:**
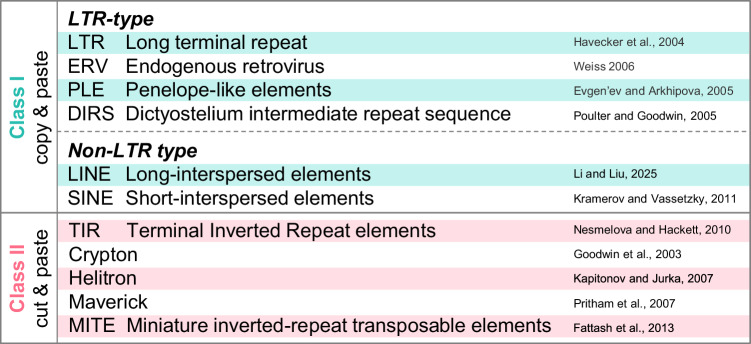
Classification of TEs.

Class I (retrotransposons): TEs in this class transpose via an RNA intermediate, replicating themselves in a “copy-and-paste” manner. The long terminal repeat (LTR) retrotransposons are characterized by the presence of LTRs at both ends, and their open reading frames (ORF) encode homologs of Gag and Pol retroviral proteins. Endogenous retroviruses (ERV) encode a third ORF, coding an envelope protein (Env), in addition to Gag and Pol. The Penelope-like elements (PLE) possess a reverse transcriptase domain similar to that of telomerase and a GIY-YIG endonuclease domain. The Dictyostelium intermediate repeat sequence (DIRS) relies on a tyrosine recombinase instead of the integrase found in typical LTR retrotransposons. The long-interspersed elements (LINE) are non-LTR retrotransposons transposing via a target-primed reverse transcription (TPRT) mechanism. The short-interspersed elements (SINE) lack autonomous mobility and rely on LINEs for their propagation. Class II (DNA transposons): TEs in this class mobilize via a DNA intermediate via a “cut-and-paste” mechanism. These are subdivided into four orders (Wicker et al, 2007). The Terminal Inverted Repeat elements (TIR) in the first order are characterized by the presence of terminal inverted repeats. The second-order Crypton uses a tyrosine recombinase for transposition. The third-order Helitron transposes through a rolling-circle replication mechanism, with single-stranded DNA intermediates. The fourth-order Maverick elements encode DNA polymerase and integrase. Miniature inverted-repeat transposable elements (MITEs) are non-autonomous and rely on the transposase enzymes of TIR for their mobility.

To mitigate such deleterious effects and maintain genomic stability, hosts have evolved multiple, partly redundant regulatory pathways to restrain TE activity. Prominent examples include the PIWI-interacting RNA (piRNA) pathway, the KRAB zinc finger protein (KRAB-ZFP)/KAP1–SETDB1 axis, and the HUSH complex (Czech et al, 2018; Rowe et al, 2010; Seczynska and Lehner, 2023). Endogenous small interfering RNAs (endo-siRNAs) also contribute to TE silencing in several organisms, including *Drosophila* (Chung et al, 2008; Ghildiyal et al, 2008). Whereas the piRNA and endo-siRNA pathways employ small RNAs that recognize TE-derived transcripts through RNA–RNA base-pairings, the KRAB-ZFP and HUSH pathways, respectively, rely on DNA- and RNA-binding proteins to identify their targets. Despite these mechanistic differences, all these systems are effective at suppressing TE activity by exploiting their distinct characteristics. The KRAB-ZFP axis is tetrapod-specific and increased rapidly in the mammalian lineage (Rosspopoff and Trono, 2023). The HUSH complex has a core subunit conserved in vertebrates, and no analogous complex has been found in major invertebrate models such as *Drosophila* (Lehner, 2025).

**Figure 1:**
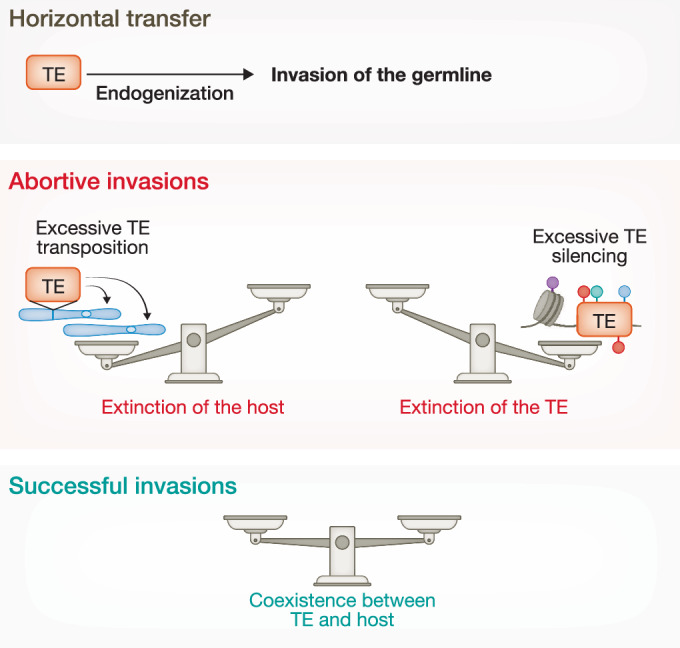
Balance between TE activity and host repression. Upon horizontal transfer of a transposable element (TE) and its integration into the germline genome (upper panel), several evolutionary scenarios of the interplay between this element and the host can be considered. If TE activity (transposition) is excessive, this can compromise host viability (middle panel, left balance). Conversely, if host defenses are excessively strong against a TE, this can fully silence it and lead to its extinction (middle panel, right balance). The third possibility (lower panel) reflects the dynamic evolutionary balance between the opposing forces employed by the TE and the host, in which neither the host nor the TE maintains lasting dominance, and which ensures TE and host sustainability.

However, when host defense mechanisms reduce TE activity below a critical threshold, lineage-specific TE families are ultimately driven to extinction (Charlesworth, 1994; Platt and Ray, 2012). Conversely, TEs that increase their copy number must also restrain their activity, as excessive transposition may compromise their own chances of survival if causing too much harm to the host they depend on. These mutual constraints drive an evolutionary arms race that settles into a dynamic equilibrium, where neither host nor TE ever gains a permanently dominant advantage (Fig. 1).

The present-day constitution of TEs observed across species thus represents the survivors of long and complex interactions between TEs and their hosts, marked by genomic conflict, selection, adaptation, and trade-off. In some cases of TE extinction, remnants of TE copies have been preserved and domesticated by the host to serve useful functions, such as providing new regulatory elements or protein domains (Feschotte, 2008; Fueyo et al, 2022; Sundaram and Wysocka, 2020). In other cases, the host biology has become more or less dependent on ongoing TE activity, despite the damage caused by new insertions (Chang et al, 2025). However, even this dependency is characterized by reciprocal antagonisms and unstable equilibria (Son et al, 2025). To better understand these dynamics, researchers have increasingly adopted ecological frameworks, applying concepts from community ecology to explore how selection, drift, and ecological constraints shape TE diversity and distribution (Brookfield, 2005; Le Rouzic et al, 2007; Venner et al, 2009).

**Figure 2:**
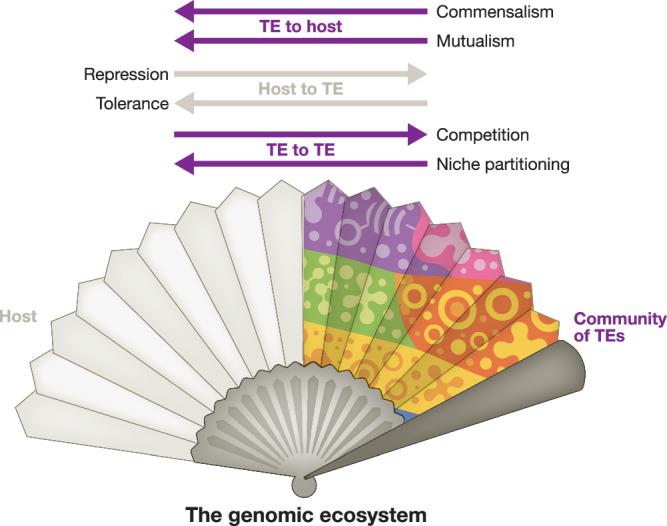
The genome ecosystem. Hosts evolved mechanisms that repress TEs and lower their fitness costs, yet complete elimination may be suboptimal because TEs can be beneficial. Thus, hosts both restrain and exploit TEs, while TEs evolve strategies to persist with limited host damage. TEs also employ strategies such as niche partitioning among TEs.

This review surveys strategies that enable host–TE coexistence from both perspectives (Fig. 2). The first section focuses on some host-centered strategies, highlighting how the *Drosophila* ovary safeguards genome integrity—and thus fertility—through piRNA-mediated repression of TEs, while also deploying modes of tolerance that permit coexistence. The piRNA pathway is highly conserved across diverse animal lineages, and knowledge of how hosts and TEs interact through this mechanism has grown substantially. In this review, however, we focus primarily on *Drosophila* and do not cover in detail other taxa such as nematodes and non-Drosophilid arthropods. Readers are referred to several excellent reviews for broader, comparative perspectives on piRNA pathways across animals (Juliano et al, 2011; Weick and Miska, 2014). The second section turns to the TE side, outlining TE strategies and the genomic “ecological niches” that permit their persistence. Viewing the genome as an ecosystem, we integrate recent insights into how antagonism, containment, and cooperation together shape shifting equilibrium states between hosts and TEs.

## Host-centered strategies for host–TE coexistence

### piRNA-mediated TE repression: basic framework

piRNAs are ~23–30-nt small RNAs that bind PIWI proteins in a stoichiometric manner to form piRNA-induced silencing complexes (piRISCs), the core effectors of TE repression (Brennecke et al, 2007; Saito et al, 2006; Vagin et al, 2006). The composition of PIWI families varies across organisms: members that localize to the nucleus mediate transcriptional gene silencing (TGS), whereas cytoplasmic PIWI proteins induce post-transcriptional gene silencing (PTGS). Although some species lack nuclear PIWI paralogs, such as silkworm, cytoplasmic PIWI proteins are essentially ubiquitous among organisms that harbor piRNAs (Hutvagner and Simard, 2008; Juliano et al, 2011).

piRNAs are generated from long, single-stranded transcripts produced from piRNA clusters, discrete genomic regions that accumulate a diverse repertoire of TE-derived sequences. These long precursors are processed in a phased manner, enabling efficient recognition and repression of cognate TE mRNAs even after they have accumulated mutations, a hallmark of TE evolution (Lawlor and Ellison, 2023). Newly invading TEs, which may initially transpose actively, are eventually incorporated into piRNA clusters (Trap model); their fragments then serve as templates for piRNA production, thereby silencing these elements (Khurana et al, 2011). In this way, piRNA clusters provide a heritable sequence registry that licenses recognition of invading elements—an “innate genome immunity” analogous in logic, though distinct in mechanism, to bacterial CRISPR systems (Malone et al, 2009; Ozata et al, 2019). This ability of piRNA clusters to tolerate and assimilate novel sequences is a defining feature of the piRNA pathway and is essential for maintaining reproductive capacity.

Many organisms also deploy PIWI–piRNAs and piRNA clusters in diverse ways (Ozata et al, 2019; Parhad and Theurkauf, 2019). For instance, mouse pachytene piRNA clusters are not particularly enriched for TEs (Gan et al, 2011; Girard et al, 2006). Moreover, in some organisms, such as mosquitoes, piRNAs are not restricted to the germline and have well-established antiviral roles (Haase et al, 2024; Miesen et al, 2016; Varjak et al, 2018).

#### The piRNA mechanism in the *Drosophila* ovary: overview

*Drosophila* encodes three PIWI proteins: Piwi, Aubergine (Aub), and Argonaute 3 (Ago3). Piwi is predominantly nuclear and, when bound to nascent TE transcripts, becomes able to conduct TGS by recruiting chromatin silencers, promoting heterochromatin assembly (Le Thomas et al, 2013; Sienski et al, 2012). Unlike many other animals, *Drosophila* does not employ DNA methylation for TE silencing because it lacks the requisite enzymatic machinery. Instead, it relies primarily on histone modifications to heterochromatinize the TE loci.

Aub and Ago3 are cytoplasmic endonucleases that execute PTGS by slicing TE transcripts; the resulting cleavage products seed the ping-pong cycle, which reinforces piRNA amplification (Brennecke et al, 2007; Gunawardane et al, 2007). Efficient piRNA amplification further depends on dedicated cofactors and specialized intracellular condensates known as nuage (Czech and Hannon, 2016; Ozata et al, 2019). Nuage is widely used as a germ cell marker in a variety of organisms (Kawaguchi et al, 2025; Voronina et al, 2011).

*Drosophila* piRNA clusters are broadly classified into uni-strand and dual-strand clusters based on their transcriptional mechanisms (Brennecke et al, 2007; Mohn et al, 2014). Uni-strand clusters are transcribed unidirectionally from their own promoters, whereas dual-strand clusters lack canonical promoters and are transcribed bidirectionally from multiple sites within the locus. The *Drosophila* ovary comprises somatic follicle cells and germ cells, which generate piRNAs from largely non-overlapping piRNA clusters. These two cell types have also evolved distinct mechanisms, including reliance on different PIWI family members, to repress TEs, as discussed below.

#### Cell type-specific diversification within one host: ovarian somatic cells vs ovarian germ cells

**Figure 3:**
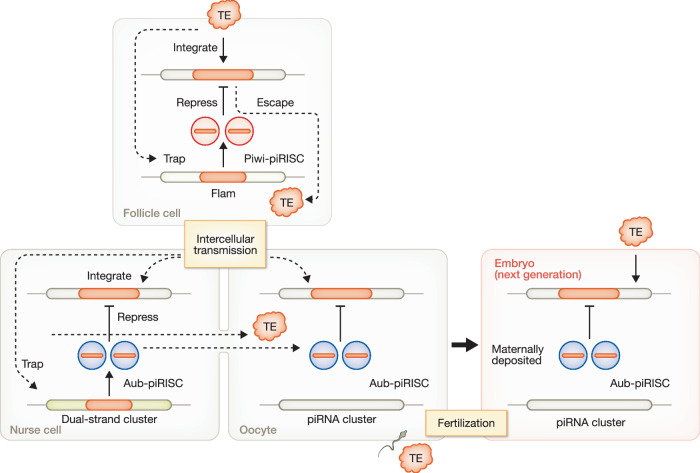
Model of TE invasion into the germline genome. In follicle (somatic) cells, Piwi-piRISC represses TEs, whereas in nurse cells and oocytes, Aub-piRISC (and Piwi-piRISC, not shown) mediates repression. TEs can access the germline genome via somatic follicle cells, through fertilization, or by direct entry into the embryonic genome. *Flam* is the uni-strand piRNA cluster active in follicle cells, while dual-strand clusters in nurse cells generate piRNAs for TE silencing.

Throughout most of *Drosophila* oogenesis, ovarian germ cells are encapsulated and protected by somatic cells (Roth, 2001; Wu and Johnston, 2009). This tissue architecture suggests that TEs first invade the somatic cell genome. Ovarian somatic cells repress TEs integrated into their own genome via Piwi-piRNA-dependent TGS, thereby limiting opportunities for vertical transmission; consequently, for inheritance, TEs must ultimately gain access to germ cells via some route (Brasset et al, 2006; Chalvet et al, 1999; Yoth et al, 2023) (Fig. 3).

piRNAs repress TEs through RNA–RNA base pairing with TE transcripts. Accordingly, TE insertions must first occur and be transcribed before silencing can take place. This requirement for a certain level of TE activity is an inherent feature of the piRNA pathway. In ovarian somatic cells, TE repression relies predominantly on Piwi-mediated nuclear silencing (TGS), whereas Aub/Ago3-dependent cytoplasmic PTGS (ping-pong amplification) is limited or absent, potentially reducing the energetic burden of sustained cytoplasmic cleavage and amplification. Breakdown of this surveillance could, in turn, create an opportunity for TEs to breach the somatic-germline barrier and invade ovarian germ cells.

Ovarian germ cells must contend not only with TEs originating in somatic cells, but also with copies embedded within their own genome that can reactivate and transpose during germ cell development. To counter these dual threats, ovarian germ cells deploy both TGS and PTGS (Fig. 3). PTGS and TGS piRISC effectors are inherited by the next generation and can repress TEs immediately at the onset of embryogenesis, targeting maternally deposited TE transcripts and/or reactivated TE transcription.

Below, we first outline the basic framework of the piRNA pathway in ovarian somatic and germ cells, and then describe how these systems diverge with respect to piRNA-cluster expression, condensate architecture, and associated cofactors. Studies of the piRNA pathway in ovarian somatic cells have been advanced by the use of cultured OSC lines. We note, however, that it is difficult—and often impractical—to draw a strict boundary between insights gained from these in vitro systems and those obtained from analyses of somatic cells within the ovary.

## Basic framework of the piRNA system in ovarian somatic and germ cells

### Ovarian somatic cells

**Figure 4:**
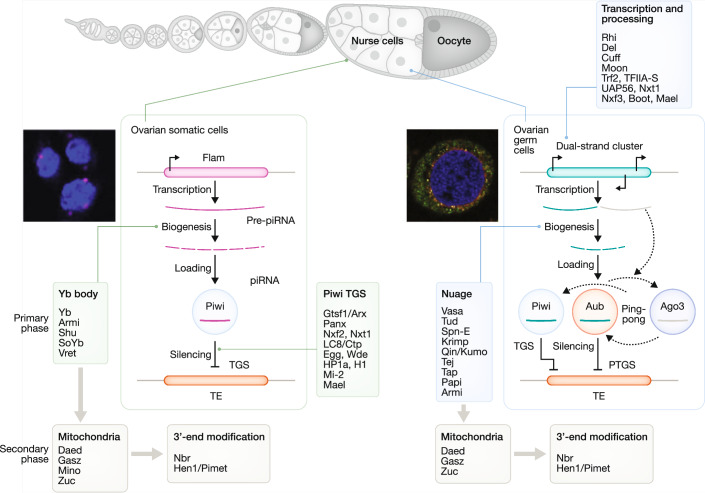
The piRNA pathway in *Drosophila* ovaries. Schematic of the piRNA pathway in ovarian somatic cells and germ cells, highlighting key factors at each stage. Pathway roles and additional features of each factor are summarized in Table 2.

These cells express Piwi but not Aub and Ago3, so within the piRNA pathway, they repress TEs exclusively via TGS. piRNAs are produced primarily from the uni-strand cluster *flamenco* (*Flam*) (Handler et al, 2013; Malone et al, 2009; van Lopik et al, 2023). Following transcription, processing (i.e., splicing, polyadenylation, and 5’-capping), and nuclear export, *Flam* transcripts are routed to Yb bodies—cytoplasmic condensates formed by liquid–liquid phase separation (LLPS) when Yb engages piRNA precursors (Hirakata et al, 2019; Ishizu et al, 2019; Qi et al, 2011; Saito et al, 2010; Sokolova et al, 2019; Szakmary et al, 2009). Within Yb bodies, long precursors undergo primary processing into shorter intermediates that are then loaded onto Piwi via their 5′ ends, thereby generating numerous Piwi-piRISC precursors (pre-Piwi-piRISC) (Ishizu et al, 2019; Saito et al, 2010) (Fig. 4).

Yb-body clients include Armitage (Armi), Sister of Yb (SoYb), Vreteno (Vret), and Shutdown (Shu), whose molecular roles and interdependencies are increasingly well defined (Table 2). The pre-Piwi-piRISC subsequently travels with Armi to the outer mitochondrial membrane, where the Gasz/Daedalus (Daed) heterodimer scaffolds Zucchini (Zuc)-dependent endonuclease cleavage, completing piRISC maturation (Handler et al, 2013; Koga et al, 2024; Munafò et al, 2019; Yamashiro et al, 2020). Minotaur (Mino) also contributes to piRISC maturation (Vagin et al, 2013), although its mechanism of action remains unclear.

**Table 2:**
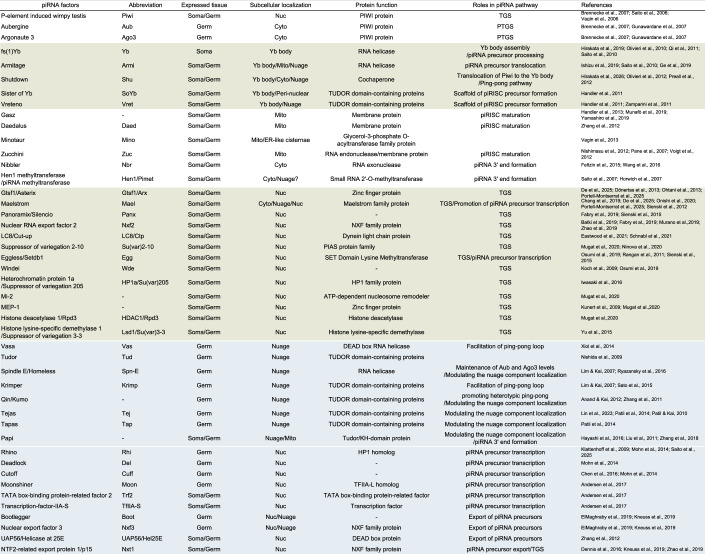
Drosophila piRNA factors and their functions.

*Soma* ovarian somatic (follicle) cells, *Germ* ovarian germ (oocyte and nurse) cells, *Nuc* nucleus, *Cyto* cytoplasm, *Mito* mitochondria, *TGS* transcriptional gene silencing, *PTGS* post-transcriptional gene silencing.

Expression domain, subcellular localization, molecular function, and pathway role are listed for factors acting in the Drosophila piRNA pathway. Row background colors map to the corresponding pathway stages in Fig. 4, indicating where each factor predominantly functions.

Subsequently, mature Piwi-piRISC is imported into the nucleus, where it initiates TGS of TEs. Piwi possesses a nuclear localization signal, which is only recognized by nuclear import factors after forming piRISC (Yashiro et al, 2018). Consequently, empty “apo” Piwi remains cytoplasmic, ensuring the formation of Piwi-piRISC. In the nucleus, upon its association with nascent TE transcripts, Piwi-piRISC forms the Piwi* complex in conjunction with Gtsf1/Asterix (Gtsf1) and Maelstrom (Mael) (De et al, 2025; Portell-Montserrat et al, 2025). Together, they recruit the PICTS/PPNP/SFiNX/Pandas complex (Batki et al, 2019; Fabry et al, 2019; Murano et al, 2019; Zhao et al, 2019) and several chromatin modifiers, including the histone H3 lysine 9 (H3K9) methyltransferase Eggless/Setdb1 (Egg), thereby promoting H3K9me3 deposition and leading to stable heterochromatin formation via the recruitment of HP1 and the linker histone H1 (Fig. 4 and Table 2) (Sato and Siomi, 2018).

### Ovarian germ cells

*Drosophila* ovarian gem cells employ both TGS and PTGS to silence TEs, and to do so, they express all three PIWI proteins. In these cells, piRNAs are generated mainly from dual-strand clusters scattered throughout the genome, as well as TE transcripts cleaved in a piRNA-driven, PTGS-dependent manner (Brennecke et al, 2007) (Fig. 4).

The combined action of TGS and PTGS ensures robust TE repression while simultaneously establishing a maternal pool of piRISCs for deposition into the embryo, enabling immediate recognition and cleavage of TE transcripts from the earliest stages of development (Fabry et al, 2021; Le Thomas et al, 2014). PTGS is predominantly carried out by Aub, which is typically loaded with antisense piRNAs and slices sense TE mRNAs. Importantly, Aub-cleaved TE mRNAs are not immediately wasted: the resulting 5′ cleavage products are converted into sense piRNAs that load onto Ago3. Aub defines piRNA 5′ ends, while their 3′ ends are subsequently matured by Zuc-dependent cleavage and exonuclease trimming by Nibbler (Nbr) (Feltzin et al, 2015; Hayashi et al, 2016; Ipsaro et al, 2012; Wang et al, 2016; Nishimasu et al, 2012).

Ago3, in turn, slices antisense transcripts, regenerating 5′ ends for antisense piRNAs that reload Aub. This reciprocal “ping-pong” cycle both enforces PTGS and amplifies the piRNA pool (Brennecke et al, 2007; Gunawardane et al, 2007). Piwi in germ cells is loaded predominantly with antisense piRNAs derived from phased trailer RNAs. Unlike in ovarian somatic cells, this loading occurs independently of Yb bodies (Fig. 4 and Table 2); nonetheless, as in ovarian somatic cells, nuclear Piwi executes TGS (Le Thomas et al, 2013; Rozhkov et al, 2013; Wang et al, 2015).

## Distinct piRNA pathways in ovarian somatic cells and germ cells

Differences between the piRNA pathways in ovarian somatic and germ cells are evident in several aspects. We first explain the mechanisms of piRNA-cluster transcription and regulation.

### piRNA-cluster transcription and regulation

#### Ovarian somatic cells

These cells mainly rely on the uni-strand *flam*, which is enriched in antisense fragments of various TEs, including gypsy family elements that produce virion-like particles (Brasset et al, 2006; Lécher et al, 1997; Pélisson et al, 1994; Song et al, 1997). *Flam* undergoes unidirectional, RNA pol II-dependent transcription from its own promoter and continuously supplies antisense precursors for resident TEs (Brennecke et al, 2007; Goriaux et al, 2014; Mével-Ninio et al, 2007). *Flam* transcripts display several mRNA-like features (Goriaux et al, 2014). The transcription factors Cubitus interruptus (Ci) and Traffic jam (Tj) are key regulators of *flam* (Alizada et al, 2025b; Goriaux et al, 2014; Rivera et al, 2025). Notably, Tj also promotes expression of Piwi and other somatic piRNA components, including Yb (Alizada et al, 2025b; Rivera et al, 2025; Saito et al, 2009). Furthermore, Tj activates a subset of TE transcripts in somatic follicle cells (Alizada et al, 2025b; Rivera et al, 2025), underscoring a finely tuned host–TE balance in which increased Tj-dependent TE expression is matched by strengthened Tj-dependent host defense.

#### Ovarian germ cells

Germ cells primarily use dual-strand clusters, which are transcribed bidirectionally (Brennecke et al, 2007; Malone et al, 2009; Mohn et al, 2014). Uniquely, these loci lack canonical promoters; instead, transcription depends on a germline-specialized apparatus comprising Rhino (Rhi), an HP1-family protein; Moonshiner (Moon), a TFIIA-L homolog; Deadlock (Del) and Cutoff (Cuff), which together with Rhi form the RDC complex (Andersen et al, 2017; Chen et al, 2016; Klattenhoff et al, 2009; Mohn et al, 2014) (Table 2). This machinery, together with nuclear export factors for precursors, such as Nxf3 and Bootlegger (Boot) (ElMaghraby et al, 2019; Kneuss et al, 2019), appears to be *Drosophila*-restricted, likely co-evolving with dual-strand clusters. Dual-strand clusters are maintained in a Rhi-dependent heterochromatic state, helping insulate cluster transcription from conventional gene expression. Dual-strand clusters may differ from each other by the specificity factors that are required for their targeting by the RDC complex (Akkouche et al, 2025; Baumgartner et al, 2022; Saito et al, 2025). Ovarian somatic cells do not express the factors required to activate these dual-strand clusters and therefore do not produce piRNAs from them (Table 2).

### Condensate architecture

Within cells, numerous reactions occur simultaneously. Recent studies indicate that many of these processes are spatially and temporally regulated through the formation of biomolecular condensates, which help prevent unwanted crosstalk between reactions (Banani et al, 2017; Choi et al, 2025).

#### Ovarian somatic cells

In these cells, piRNA biogenesis is organized around perinuclear, non-membranous Yb bodies. Yb bodies are *Drosophila*-specific, consistent with the restricted phyletic distribution of Yb (Chary and Hayashi, 2023). Although a subset of genic piRNAs is produced independently of Yb bodies but in an Armi- and Zuc-dependent manner (Ishizu et al, 2019), Yb appears to optimize somatic TE-directed piRNA output. Functionally, Yb bodies promote efficient TGS by channeling newly exported precursors into cytoplasmic processing and rapidly loading Piwi. Consistent with this, unloaded (“apo”) Piwi is less stable and/or turns over faster; efficient channeling within Yb bodies therefore both loads and stabilizes Piwi.

#### Ovarian germ cells

In germ cells, perinuclear, membrane-less nuage act as hubs for PTGS, providing a platform for Aub/Ago3-driven ping-pong amplification (Lim and Kai, 2007). Nuage are conserved in germlines across many animals. Their perinuclear positioning facilitates the capture of both cluster-derived precursors and TE transcripts as they exit the nucleus, enabling rapid cleavage of transcripts that escape TGS and bridging the latency before TGS is fully established. Studies using silkworm ovarian cell lines indicate that nuage can be classified into multiple condensate types based on client factors and function (Namba et al, 2022; Nishida et al, 2020; Xiol et al, 2014); whether a similar functional subdivision exists in *Drosophila* nuage remains to be determined.

### Specific cofactors and their functional platforms

Ovarian somatic cells and germ cells sometimes use the same cofactors, while other times they utilize their own unique cofactors (Table 2). Here, we introduce the cofactors used within each cell type.

#### Ovarian somatic cells

Biogenesis proceeds in two stages (Fig. 4): First, a primary phase occurs in Yb bodies and requires Yb, SoYb, Vret, Shu, and Armi (Handler et al, 2011; Hirakata et al, 2019; Ishizu et al, 2019; Olivieri et al, 2012; Preall et al, 2012; Qi et al, 2011; Saito et al, 2010) (Table 2). Second, Zuc-dependent maturation takes place on the mitochondrial outer membrane, scaffolded by the Gasz/Daed heterodimer (Koga et al, 2024; Munafò et al, 2019; Yamashiro et al, 2020). Within Yb bodies, clients show hierarchical localization (Yb → Armi → SoYb/Vret heterodimer) (Hirakata et al, 2019). Shu concentrates Piwi in Yb bodies to promote pre-Piwi-piRISC assembly (Hirakata et al, 2026), whereas the SoYb/Vret heterodimer serves as a scaffold for this step.

Gasz and Daed assemble a heterodimer but play non-equivalent roles. Loss of Gasz nearly abolishes Piwi-piRISC formation, while Daed primarily stabilizes Gasz (Koga et al, 2024; Munafò et al, 2019; Yamashiro et al, 2020). Daed is dipteran-specific and engages Armi (already bound to pre-piRISC) to drive 3′-end refinement; disrupting their interaction yields 3′-untrimmed piRNAs, lowers piRISC levels, and weakens silencing. Interestingly, mammals lack Daed but employ the exonuclease Poly(A)-specific ribonuclease-like domain containing 1 (PNLDC1) to trim 3′ ends after Zuc cleavage; PNLDC1 deficiency similarly disrupts piRNA function (Anastasakis et al, 2016; Ding et al, 2017; Nishimura et al, 2018; Zhang et al, 2017). *Drosophila* lacks PNLDC1, indicating mechanistically distinct yet functionally analogous solutions, illustrating lineage-specific adaptation to TE pressure.

As an aside, the L(3)mbt-Ovo axis broadly suppresses expression of germ-specific piRNA factors in ovarian somatic cells (Coux et al, 2018; Sumiyoshi et al, 2016; Yamamoto-Matsuda et al, 2022; Alizada et al, 2025a). Loss of L(3)mbt allows Ovo ectopic expression, which induces aberrant expression of ping-pong factors in ovarian somatic cells and causes female sterility.

#### Ovarian germ cells

piRNA maturation requires Vasa, Spindle-E/Homeless (Spn-E), Krimper (Krimp), Kumo/Qin, Tejas (Tej, Tdrd5), Tapas (Tap, Tdrd7), and Tudor (Tud) (Anand and Kai, 2012; Lim and Kai, 2007; Lin et al, 2023; Nishida et al, 2009; Patil et al, 2014; Patil and Kai, 2010; Ryazansky et al, 2016; Sato et al, 2015; Xiol et al, 2014; Zhang et al, 2011), alongside shared components such as Zuc and Armi (Fig. 4 and Table 2). Most germ cell-biased factors are nuage-associated. In the nuage, Aub/Ago3-mediated piRNA amplification takes place. Vasa, an example of a factor specifically expressed in ovarian germ cells and localized to the nuage, facilitates the transfer of RNA fragments generated by Aub-mediated cleavage to Ago3 (Nishida et al, 2015; Xiol et al, 2014). Qin/Kumo promotes efficient piRNA amplification by suppressing homotypic Aub-Aub ping-pong (Zhang et al, 2011). Meanwhile, Tud is likewise specifically expressed in ovarian germ cells and localized to the nuage, and although it is known to bind Aub and Ago3 (Nishida et al, 2009; Kirino et al, 2010), its precise function remains unknown, reflecting the complexity of this machinery. Given these distinct modules, a large cast of factors is indispensable. While many roles are genetically defined, epistasis and hierarchy remain incompletely resolved; a cohesive systems-level model in *Drosophila* is still emerging.

### Programmed DNA elimination as an extreme mode of host–TE coexistence

Section “piRNA-mediated TE repression: basic framework” has discussed TE repression in *Drosophila* ovaries in detail as one mode of host–TE coexistence. Here, as a contrasting example, we outline the TE silencing mechanism in ciliates. In these organisms, programmed DNA elimination (PDE) achieves repression not through transcriptional control, but through the physical excision of TE-containing DNA from the somatic genome (Cheng et al, 2010; Dubois et al, 2012). Strikingly, TE sequences are retained in the germline (Arnaiz et al, 2012; Hamilton et al, 2016). This dichotomy suggests that TEs can carry sustained physiological and evolutionary value, acting as a reservoir of sequences with the potential to drive innovation over time. In this sense, PDE offers an illuminating perspective on host–TE coexistence, a central theme of this review.

In ciliates such as *Paramecium*, *Tetrahymena*, and *Oxytricha*, small-RNA-directed TE repression is indeed executed via an extreme strategy: PDE of TE-rich sequences from the somatic genome. These unicellular eukaryotes contain two nuclei with a shared cytoplasm: a transcriptionally silent germline micronucleus (MIC) and a transcriptionally active somatic macronucleus (MAC). During sexual reproduction, a new MAC is generated from the MIC and developmentally regulated small RNAs known as scan RNAs (scnRNAs), which guide the selective removal of internal eliminated sequences (IESs) from the new MAC (Betermier and Duharcourt, 2014; Chalker et al, 2013; Gao et al, 2023). Because IESs are enriched for TE sequences, their excision effectively purges TEs from the somatic genome.

In *Paramecium* and *Tetrahymena*, scnRNAs are generated in a Dicer-dependent fashion (Malone et al, 2005; Mochizuki and Gorovsky, 2005), resembling endo-siRNA biogenesis but loaded onto Piwi-related proteins such as Ptiwi09 and Twi1p (Bouhouche et al, 2011; Mochizuki et al, 2002). Although scnRNAs are initially produced from the entire MIC genome, a subtraction process enriches the scnRNA pool with sequences corresponding to IESs and TEs (Charmant et al, 2025; Schoeberl et al, 2012; Wang et al, 2024). Then these scnRNAs guide the accurate DNA excision of target loci by domesticated PiggyBac transposases (Pgm in *Paramecium*, Tpb2 in *Tetrahymena*) (Baudry et al, 2009; Bischerour et al, 2018; Cheng et al, 2010) through deposition of repressive chromatin marks (H3K9me3, H3K27me3) by the Polycomb Repressive Complex 2 (PRC2) (Balan et al, 2024; Miró-Pina et al, 2022; Xu et al, 2021).

*Oxytricha* employs a related but mechanistically distinct form of PDE. In this species, scnRNAs are largely complementary to MAC-destined sequences, and appear to protect these regions from elimination, whereas unprotected segments—many derived from active Tc1/mariner-type transposons—are excised (Fang et al, 2012). How scnRNAs confer protection against transposase-mediated removal remains unclear.

### Host tolerance of TEs: biological relevance and underlying strategies

Due to frequent horizontal transfers (Zhang et al, 2020) and TE reactivations, host genomes have likely been the theaters of multiple attempts of TE invasion, only some of which (the present-day TEs) have eventually ended up in successful colonization phases (Blumenstiel, 2019). Indeed, as outlined above (Fig. 1), while unleashed transposition is expected to cause host demise and, consequently, failure of the invasion, excessive repression of the TE by the host may likewise result in a completely aborted invasion. Moreover, while host fitness is obviously preserved by piRNAs-mediated TE repression, it may also suffer from off-targeting, when excessive repression leads to unintended spreading of repressive epigenetic marks into neighboring host genes (Choi and Lee, 2020).

However, TE repression may also participate in the success of TE invasions (the double-edged sword paradox) by attenuating the deleterious effects of mobilization and therefore reducing the risk of TE elimination by selection (Lu and Clark, 2010). Generalization of this paradoxical observation to a wide survey of metazoans, confirmed that species with piRNAs do tend to contain a larger number of TE families, each of which having fewer copies in their genome (Schneider et al, 2021). Since TEs can, in the long term, fuel host regulatory innovations (Bourque et al, 2018; Fueyo et al, 2022), such “tolerant” TE-rich hosts are more likely to have adapted to new environments. That is probably why TE repression has been conserved in present-day host-TE interactions, although not always at optimal levels: permitting limited transposition would preserve essential functions while enabling long-term adaptation. Accordingly, piRNAs operate less as a blunt instrument of resistance than as gatekeepers of managed coexistence, tolerating controlled TE activity under realistic constraints while safeguarding genome integrity. In this tolerance model, particularly under stress, hosts would exploit manageable transposition rates and the resulting genetic diversity instead of striving for absolute repression.

Moreover, bringing newly invading TEs under control requires the prior insertion of at least one TE copy into piRNA clusters, enabling the subsequent production of cognate piRNAs. This creates a lag during which some activity must be tolerated. For instance, at the onset of P element invasion, *Drosophila* premeiotic cells can tolerate low levels of DNA breakage induced by the initial actively transposing copies (Jansen et al, 2024). Above this threshold, germ cell loss imposes positive selection for P element insertions competent for piRNA production. The degree of this tolerance varies throughout germline development (Jansen et al, 2024). This developmental modulation of tolerance appears to be a conserved feature, as similar windows of transiently relaxed TE silencing have been observed in both mammals (Grow et al, 2015; Sakashita et al, 2023) and *Drosophila* (Dufourt et al, 2014). Tolerance thresholds also depend on genetic factors (Kelleher et al, 2018) and on environment, particularly temperature: piRNA defense initiates more slowly at low temperatures and more rapidly at high temperatures (Kofler et al, 2018).

Another mechanism of tolerance may explain how, within only decades, the P element managed to colonize natural fly populations worldwide. This rapid spread was likely mediated by frequent fly migrations between naïve and already colonized populations, facilitated by intense human mobility. When P-containing males mate with naïve females, their progeny exhibit the so-called P-M hybrid dysgenesis syndrome (Khurana et al, 2011), which is due to the inability of the paternal genome to immediately initiate piRNA production in the absence of maternally deposited piRNAs. This sterility is partly reversible with age and results from a DNA damage-induced checkpoint that transiently blocks germinal development, allowing piRNA production to gradually resume (Khurana et al, 2011; Moon et al, 2018). In the absence of this checkpoint, DNA breaks caused by unleashed transposition lead to complete sterility, thereby preventing vertical transmission and amplification of the P element.

## Genetic conflicts considered from the TE side

**Figure 5:**
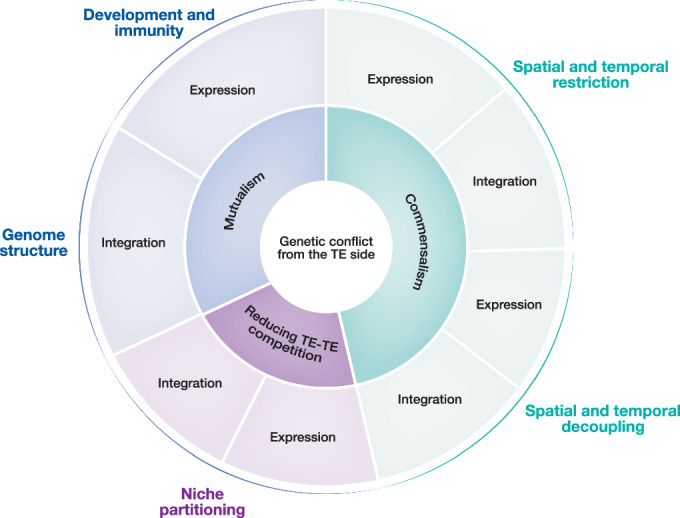
Resolution of the genetic conflicts from the transposable element side. Schematic representation of three different types of strategies employed by TEs. Each type of strategy involves different niches of expression and integration that TEs modulate in specific ways. **Commensalism**: *Spatial and temporal restriction: expression/integration* (Carter et al, 2022; Cheung et al, 2016; Ke, 1997; Kirchner et al, 1995; Lawlor et al, 2021; Sultana et al, 2017; Xie et al, 2001; Zou and Voytas, 1997; Sakashita et al, 2023; Ghanim et al, 2020; Kojima et al, 2016)*. Spatial and temporal decoupling: expression/integration* (Senti et al, 2025; Varoqui et al, 2025; Voichek et al, 2025; Wang et al, 2018). **Mutualism:** *Genome structure: Integration* (Casacuberta, 2017; Chabot et al, 2024; Nelson et al, 2023; Kojima et al, 2016). *Development and immunity: Expression* (Brégnard et al, 2016; Chang et al, 2025; Grow et al, 2015; Jachowicz et al, 2017; M’Angale et al, 2025; Mathavarajah and Dellaire, 2024; Moore et al, 2021; Percharde et al, 2018; Wang et al, 2022; Sakashita et al, 2023; Larouche et al, 2024). **Reducing TE–TE competition**: *Niche partitioning: Expression/integration* (Klumpe et al, 2025; Senti et al, 2025; Son et al, 2025; Varoqui et al, 2025; Ye et al, 2005).

TEs face a persistent challenge: how to propagate within host genomes while minimizing their negative impact on host fitness that eventually threatens their own long-term survival. To address this, these parasites seem to have developed a sophisticated set of adaptive strategies (Fig. 5). Most of these strategies allow them to exploit specific expression and integration niches, both spatial and temporal, that facilitate safe replication and vertical transmission, while reducing the deleterious effects on the host (commensalism). Some TEs can adapt even further by integrating themselves so deeply into the host’s genome function that it becomes dependent on some of their activities (or components). In this case, the parasitic relationships turn into a form of mutualism.

### Commensalism: neutral expression and integration strategies

To ensure their long-term maintenance within a host organism, TEs must integrate new copies into the DNA of germline cells. TEs that cannot cross cell boundaries must therefore express in these cells, which thus constitute an ecological niche for their replication cycle, from expression to integration. However, the syncytial nature of germline cysts (Spradling, 2024) may be exploited to transfer genetic material from cell to cell through cytoplasmic bridges, resulting in spatial decoupling of the expression and integration steps. Moreover, some TEs have evolved the capacity to infect neighboring cells to ultimately integrate into the germline genome while expressing in the surrounding somatic tissues.

#### Restriction of expression to the germline or to specific developmental windows

Several TEs restrict their full transposition activity to germline cells. For instance, in *Drosophila*, the P element (Table 3) undergoes alternative splicing that produces a functional transposase mostly in the germline. Conversely, in somatic tissues, retention of the third intron produces a truncated protein that acts as a transcriptional repressor that safeguards somatic cells from potential genomic instability (Jensen et al, 2008; Laski et al, 1986; Roche et al, 1995). This strategy ensures that active transposition indeed operates in the appropriate cell lineage for vertical transmission while preventing “useless” and harmful somatic expression. Because the PSI protein sequence (the P element somatic inhibitor regulating P element alternative splicing) is highly conserved among species independently of transposable element presence, this somatic repression is not viewed as a host adaptation (Lee and Langley, 2012).

**Table 3:**
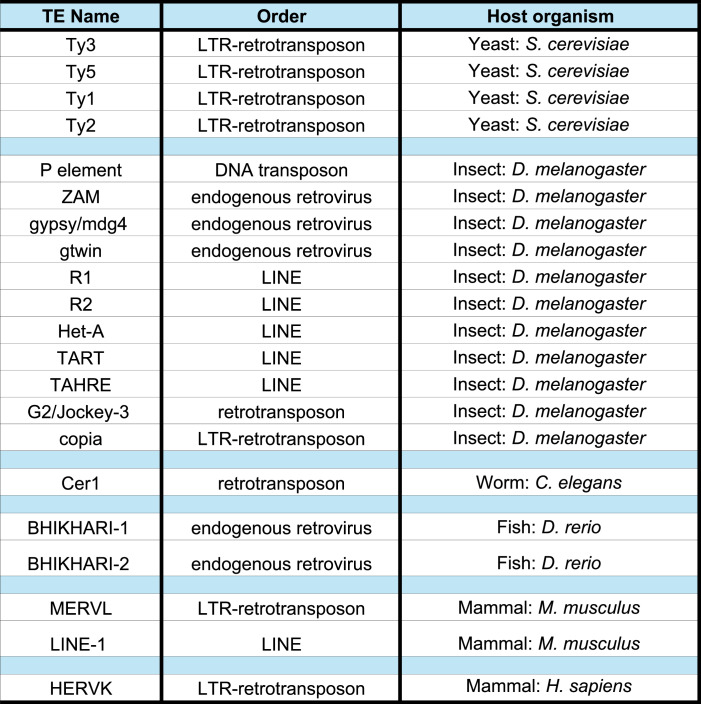
Summary of transposable elements cited in the review.

Table summarizing the name, the order, and the preferred host organism of the different transposable elements cited in the review.

Beyond simple tissue-specific expression, some TEs confine their transcription to particular developmental stages when, as mentioned above, cells are especially tolerant to the negative consequences of TE expression. In *S. cerevisiae*, Ty3 and Ty5 LTR retrotransposons are barely expressed during vegetative growth but are induced upon sexual reproduction (Ke, 1997; Kinsey and Sandmeyer, 1995). In mammals, early embryogenesis provides a similar permissive window for TE expression (Carter et al, 2022). Following fertilization, the zygote and early blastomeres exhibit a remarkable ability to buffer genetic perturbations and to correct developmental defects (Power and Tam, 1993). In mice, MERVL is transiently upregulated specifically at the two-cell stage, after which it is rapidly silenced; this brief window of activity coincides with a period when alterations in single cells can be mitigated by neighboring cells or compensated through cell-division dynamics (Sakashita et al, 2023). In humans, likewise, HERVK expression leverages the permissive window from the eight-cell stage until early blastocyst formation, before DNA methylation patterns in the inner cell mass shut down retroviral transcription (Grow et al, 2015). An expression burst of Y-chromosome-linked TEs has also been reported in *Drosophila* spermatocytes (Lawlor et al, 2021). Similarly, during early stages of *Drosophila* oogenesis, there is a short spatiotemporal window when some TEs may take advantage of some partial release of the piRNA-mediated host control (Dufourt et al, 2014). This so-called ‘Piwiless pocket’, has been proposed to allow the insertion of new TEs in the developing germline genome (Théron et al, 2018). By exploiting these narrow windows of opportunity, TEs are thought to minimize host harm while ensuring expression into the appropriate cells for future integration into the germline.

#### Spatial decoupling of expression and integration

Some TEs employ an elaborate strategy *via* decoupling the locations of the transcription and integration steps of their retrotransposition cycle. In this case, the element is transcribed in one cell type but can integrate into another one. In *Drosophila*, ERVs have gained the ability to infect the germline owing to their *env* gene, horizontally acquired from baculoviruses (Malik et al, 2000; Rohrmann and Karplus, 2001; Senti et al, 2025). Tissue-specificity of the ZAM ERV expression includes the embryonic somatic primordial cells which surround the primordial germ cells (PGCs) where ZAM integration likely occurs (Varoqui et al, 2025). In the ovaries, all infectious ERVs, including ZAM, are specifically transcribed in the somatic follicle cells (Senti et al, 2025), which surround the oocyte that they may infect. Interestingly, this strategy, whereby follicle cell-specific expression is followed by oocyte infection, has been independently invented in the *mdg1* clade of LTR retrotransposons lacking *env* gene (Voichek et al, 2025). In this example of convergent evolution, the mechanism of cell-to-cell transmission is similar to the system used by non-enveloped fusogenic viruses. Even if TE mobilization wreaks havoc in follicle cells, these terminally differentiated support cells are anyway destined to undergo degeneration at the end of oogenesis. The non-infectious TEs of the ERV clade, i.e., those that lack a functional *env* gene, as well as LINEs, are expressed in nurse cells, which are germinal support cells that also undergo programmed cell death at the end of oogenesis. Despite being transcribed in these “dead-end” lineages, these TEs can hijack the ovarian microtubule-dependent cell-to-cell transport mechanisms to transmit their intermediates of transposition (viral particles and RNAs, respectively) into the egg, where integration takes place (Senti et al, 2025; Wang et al, 2018). This adaptation could allow TEs to use expression-friendly environments that do not compromise long-term genomic integrity, while still achieving successful vertical transmission.

#### Preference for integration into specific insertional niches

Many TEs display distinct insertion site preferences within host genomes. These preferences might reflect the fact that certain genomic regions could tolerate transposition without suffering from major deleterious effects on host fitness. Such regions might therefore represent insertion niches where the negative consequences of integration are effectively buffered (Sultana et al, 2017). However, to which extent these sites qualify as true “safe havens” remains debated. Below, we provide examples illustrating how different TEs accommodate their integration into specific niches within their host genomes.

In *S. cerevisiae*, Ty1 and Ty2 LTR-retrotransposons integrate upstream of tRNA genes transcribed by RNA Polymerase III, regions generally devoid of essential protein-coding genes (Cheung et al, 2016; Kirchner et al, 1995). Similarly, Ty5 targets heterochromatin telomeres and the silent mating-type locus (Xie et al, 2001; Zou and Voytas, 1997). In a wide range of organisms, the R2 LINE specifically targets the ribosomal DNA (rDNA) repeats (Kojima et al, 2016). To avoid collision between transcription and replication machineries, cells have adopted a compartmentalization of the multiple rDNA copies into those that are actively transcribed and those that initiate replication. They therefore must carry a greater copy number of rDNAs than minimally required for sufficient rRNA cell production (Nelson et al, 2023). This could also explain why disruption of a few rDNA repeats by TEs does not impair ribosomal function, thereby minimizing harmful effects on host fitness.

Instead of being “safe havens”, some preferred insertion sites can be beneficial for the TE. The P element in *Drosophila* has a propensity to target specific hotspots within the genome, predominantly located in open chromatin or in regions that are actively transcribed in early germ cells, where transposition occurs. More precisely, P elements appear to target GC-rich regions adjacent to gene promoters, which function as replication origins (Spradling et al, 2011). Interestingly, it has been hypothesized that P elements, by targeting these loci, leverage host DNA replication to amplify their genomic copy number (Spradling et al, 2011). Such insertions into promoter regions might represent a genomic threat but can be tolerated if the second allele of the targeted gene remains functional.

#### Timing of integration during germline development

Different types of TEs face different constraints concerning the stage of integration during germline development. For instance, DNA transposons, which rely on a cut-and-paste mechanism, can only amplify in replicating cells. In contrast, some retrotransposons can be expressed, retrotranscribed, and integrated at later stages of gametogenesis that do not necessarily have to proliferate. Within this molecular framework, even when a TE’s transcription and/or integration are germline-restricted, the precise developmental stage of integration can determine the likelihood of a harmless outcome.

In *Drosophila*, the ovarian germline stem cells (GSCs) and the corresponding spermatogonial cells of the testis maintain totipotency and an “immortal” status, and disruptions here can have catastrophic consequences for fertility and the species’ genome integrity. Strikingly, TEs seem to preferentially integrate later, at more differentiated germline stages in which tolerance to double-strand breaks (DSBs) is higher (Jansen et al, 2024; Wang et al, 2018). For instance, the *copia* LTR retrotransposon is able to transpose in the *Drosophila* testis (Pasyukova et al, 1997), but, even though copia capsids start to accumulate in the spermatogonial cytoplasm, they cannot enter the nucleus before spermatocyte differentiation. This is how *copia* likely avoids insertions in the most vulnerable undifferentiated germ cells (Klumpe et al, 2025).

Some ERVs in *Drosophila*, such as gtwin, show an integration preference for very early embryonic stages, before segregation of somatic and germline lineages (Varoqui et al, 2025). Since the zygotic nuclei at this stage are within a syncytium that can compensate for the DNA damage caused to individual nuclei (Sullivan et al, 1993), integration at these early time points is thought to have minimal impact on development. As mentioned above, the ERV ZAM can infect primordial germ cells (PGCs) at later embryonic stages (Varoqui et al, 2025), when PGCs can still proliferate to compensate for PGC loss due to potential DNA damage (Gilboa and Lehmann, 2006).

### Mutualism: when TE and the host need each other

Some TEs have evolved to become essential components of host genome function. While many active TEs persist simply because they are not deleterious enough to be purged, others evolve into a state of molecular “dependency” in which the host genome biology relies on their activity. This state reflects an unstable equilibrium, where the TE remains potentially harmful, but provides functions that the host cannot easily replace. Over evolutionary time, and under specific selective and genomic constraints, some TE copies/fragments have been domesticated. In such cases, these domesticated remnants no longer behave as active mobile elements, but rather serve as co-opted genetic tools for the host’s regulatory, structural, or developmental processes (Chakrabarty et al, 2023; Cosby et al, 2019). Below are several examples of functions that have become important to the host even though they are, at least partly, provided by the expression and/or mobilization of still active TEs (Fig. 5).

#### Genome maintenance and TE mobilization

Unlike most eukaryotes, *D. melanogaster* lacks a canonical telomerase. Instead, three LINEs, Het-A, TAHRE, and TART, specifically transpose onto chromosome ends in tandem arrays (Casacuberta, 2017). By adding repeats via transposition, these elements maintain telomere length and preserve chromosome stability. Another example involves the R2 retrotransposon, which integrates exclusively into the rDNA repeats of many organisms (Kojima et al, 2016). In *Drosophila*, when rRNA gene dosage falls below a certain threshold, R2 endonuclease-mediated double-strand breaks stimulate recombination among rDNA units, leading to rDNA copy number expansion (Nelson et al, 2023). Finally, the *D. melanogaster* G2/Jockey-3 element is an active TE that appears to have evolved a preference for inserting into centromeric DNA. While this targeting may serve the element’s own selfish propagation, its transcription within centromeres could also contribute to centromere identity and function (Chabot et al, 2024).

#### Host development and TE expression

In mice, MERVL is transiently expressed at the two-cell stage, and its Gag protein stabilizes the totipotent state by preventing the unconventional prefoldin RPB5 interactor from protecting key pluripotency transcription factors (OCT4, SOX2) from degradation. MERVL Gag thus participates in establishing the transcriptional network required for totipotency (de la Rosa et al, 2024). Moreover, full-length MERVL transcripts themselves may act as chromatin organizers or RNA scaffolds, guiding the assembly of regulatory complexes that specify cell fate at the earliest embryonic stage (Sakashita et al, 2023). Similarly, LINE-1 transcripts are highly expressed after fertilization in the mouse. At these early stages of development, their expression is necessary to modulate global chromatin accessibility, a chromatin feature essential for proper subsequent development (Jachowicz et al, 2017). Later, at the blastocyst stage, LINE-1 expression helps recruit repressive chromatin factors (e.g., Nucleolin, TRIM28/Kap1) to the Dux and MERVL loci, modulating zygotic gene activation. By acting as a long noncoding RNA scaffold, LINE-1 participates in the epigenetic transitions that control totipotency and early differentiation (Percharde et al, 2018).

A recent study in Zebrafish, *Danio rerio*, pointed out that recently active TEs might contribute to crucial embryonic development (Chang et al, 2025). The authors of this study characterized the embryonic functions of BHIKHARI-1 (Bik-1) and BHIKHARI-2 (Bik-2, also known as crestin), two related families of endogenous retroviruses specifically expressed in the mesendoderm and neural crest, respectively. They demonstrated that Gag proteins encoded by Bik-1 and Bik-2 are essential for somatogenesis and neural crest development, respectively, via modulation of cell adhesion and/or migration. This illustrates how the expression patterns of two structurally related Gag proteins precisely fit with their recently acquired roles in distinct developmental pathways.

#### Host immunity and TE expression

Expression of certain TEs can trigger innate immune pathways that also respond to exogenous viruses. Double-stranded DNA (dsDNA) intermediates produced by LINE-1 can activate the cGAS–STING pathway upon autodetection of cytosolic dsDNA generated by reverse transcription of LINE-1 mRNA (Brégnard et al, 2016; Mathavarajah and Dellaire, 2024). During early mammalian embryogenesis, the expression by HERVK of the Rec accessory protein (resembling HIV Rev) upregulates the interferon-induced transmembrane protein IFITM1, creating a barrier against both endogenous retroviral replication and exogenous viral infection (Grow et al, 2015). Since HERVK activation is OCT4-dependent and occurs under genome-wide hypomethylation, it has been proposed that primordial germ cells (PGCs) may similarly harness HERVK to fend off viral threats (Grow et al, 2015). In *Drosophila melanogaster*, the gypsy/mdg4 ERV is expressed during metamorphosis and licenses antiviral responses in adults by priming the innate immune machinery (Wang et al, 2022). Recently, it has been proposed that interactions between TEs and adaptive immune response could contribute to the establishment of T-cell tolerance and prevention of autoimmunity (Larouche et al, 2024).

From another point of view, while these innate immune pathways provide a first line of defense against exogenous viral infection, in the germline, the piRNA pathway triggers some adaptive immunity against TEs per se. Indeed, TE integration into piRNA clusters imposes a feedback loop: TEs self-limit their own proliferation by donating sequences into the piRNA network. Because piRNA clusters are typically heterochromatic and safe for insertions, TEs often “accidentally” land there; however, once integrated, they contribute to their own silencing, stabilizing the TE–host equilibrium.

#### Host biology and TE-derived VLPs

In *C. elegans*, an interesting example of nucleic acid-based sort of immunity relies on the production by a TE of infectious virus-like particles (VLPs). Indeed, a recent insertion of the full-length Cer1 retrotransposon produces, in the germline, VLPs that are assumed to contain bacterial pathogen-encoded small RNAs and to carry this infectious information to the brain where pathogen-avoidance behavior is thus learned. Interestingly, these particles would not only mediate inter-tissue but also inter-worm transfer of this infectious signal, providing the basis for trans-generational inheritance of this acquired adaptive behavior (Moore et al, 2021).

The *Drosophila copia* retrotransposon, beyond its parasitic role, packages RNAs into VLPs that are transmitted across synapses in neuromuscular junctions. The amount of these copia VLPs may modulate the level or the function of Arc1, a domesticated Gag-like protein that regulates synapse formation and structural plasticity. By “hijacking” endogenous vesicular trafficking pathways, copia VLPs thus contribute to synaptic homeostasis and plasticity, illustrating a TE-derived mechanism co-opted for neuron–muscle communication (M’Angale et al, 2025).

### Does TE niche partitioning result from TE-TE competition?

Horizontal transfer of TEs represents one of the most potent mechanisms fueling their diversification and ecological turnover across species (Zhang et al, 2020). By crossing species barriers, TEs escape long-term coevolutionary constraints with their hosts and colonize new genomic environments that offer fresh ecological opportunities. A recent study tracking the dynamics of TE horizontal transfer in *Drosophila* suggests that some TEs have been rapidly transferred over a short evolutionary time scale and spread throughout host populations (Pianezza et al, 2025). This indicates that horizontal transfer of TEs is a common event in organisms. Such transfers can be viewed as invasion events in an ecological sense, where a newly introduced TE family competes with resident elements for host resources and genomic space. Depending on their relative fitness, resulting from transposition efficiency, host regulatory mechanism, and integration preferences, these foreign TE families may either competitively replace or coexist with endogenous TE families. The coexistence might require the emergence of specific expression and/or integration niches for the different TE families.

#### Expression niche partitioning in the same tissue

Within a single tissue or even at a single developmental stage, the expressions of different TE families often segregate spatially or temporally, as an apparent result of competition for transcription factors and replication machinery. In *Drosophila*, recent studies have shown that many ERV families are expressed in distinct subpopulations of the ovarian somatic cells (Senti et al, 2025; Varoqui et al, 2025). Some ERVs are active only early, in the germarium, whereas others are restricted to later stages and distinct subpopulations of follicle cells. By partitioning expression into different somatic niches, TE families are assumed to avoid exhausting host resources and minimize cumulative damage in any single cell type.

#### Evidence for the exclusivity of integration niches

Even when multiple TEs share a common integration site (e.g., rDNA repeats), they often avoid direct competition by targeting subtly different sub-regions or by employing mutually exclusive integration mechanisms. For example, the *Drosophila* retrotransposons R1 and R2 both insert into the 28S rDNA unit, and their insertion sites are separated by only ~74 base pairs. Studies have shown that insertion of one element (e.g., R1) modifies local nucleosome positioning and chromatin structure in a manner that sterically or structurally inhibits subsequent targeting by the other element (R2) (Ye et al, 2005). Consequently, R1 and R2 insertions within the same rDNA unit are mutually exclusive, reducing direct competition while leveraging target redundancy to ensure that both can coexist in the same host.

Evidence for TE competition in shared insertion niches has also been recently provided by a phylogenetic study of the LINEs that “cap” the telomeres of a hundred *Drosophila* species (Son et al, 2025). As mentioned above for *Drosophila melanogaster*, several clades of these “telomeric TEs” may transpose in the same telomeric niche to maintain telomere length in the absence of telomerase. However, the authors observed that frequent inter-host horizontal transfers of the 6 identified clades (TR1-6) may have led to the replacement of endogenous clades by the invaders. In the *Sophophora* subgenus, for instance, the TR1 ancestral telomeric TEs have been repeatedly replaced by the apparently more fit TR2 telomeric TEs originating from the *Drosophila* subgenus. Such events mirror migration phenomena between natural communities, where an introduced species may outcompete resident taxa by exploiting common resources more efficiently. These recurrent invasions illustrate how horizontal transfers act as a continuous source of ecological alteration within host genomes, analogous to the arrival of invasive species in natural ecosystems.

#### Evidence for intra-family competition between autonomous and non-autonomous elements

Experimental evolution studies further revealed that TE diversity is also shaped by complex interactions among TEs of the same family. For example, in vivo evolution experiments with mariner elements in *Drosophila melanogaster* populations have revealed hyperparasitism between autonomous and non-autonomous copies (Robillard et al, 2016). Non-autonomous elements, lacking their own transposase, can parasitize transposase-competent autonomous copies, thereby reducing the latter’s amplification success. This “intra-family” competition can drive the extinction of the corresponding TE family. This phenomenon is reminiscent of the concept of predator–prey interactions applied to ecological communities (Capy, 2021; Le Rouzic et al, 2007; Lokta, 1925; Volterra, 1926) where the predator (the non-autonomous element) will proliferate as long as the prey (autonomous copies) remains to sustain its amplification. However, once the prey population collapses due to overexploitation, the predator population also declines due to the loss of its required resource (here, the transposase enzyme), leading ultimately to the extinction of both. This dynamic mirrors classical ecological cycles of resource depletion and collapse, highlighting how intra-genomic ecological relationships can govern TE survival and long-term evolution.

## Concluding remarks

In this review, we have only covered a small part of the rich literature reporting the innovations made by transposable elements and their hosts to reach dynamic equilibria, characterized by both conflict-driven defense mechanisms and niche-based strategies that allow TEs to persist with minimal harm and host to benefit from controlled TE activity. Their intricate interactions make it difficult to consider them from a single partner’s perspective. Emerging insights into piRNA-mediated silencing, immune pathway crosstalks, and TE co-option for developmental or regulatory functions underscore the complexity of this genomic “ecosystem.” Ultimately, viewing genomes through an ecological lens reveals how cooperation and competition between TEs and hosts shape genome evolution and stability.

## Supplementary information

Peer Review File

## References

[CR1] Akkouche A, Kneuss E, Bornelöv S, Renaud Y, Eastwood EL, van Lopik J, Gueguen N, Jiang M, Creixell P, Maupetit-Mehouas S et al (2025) Binding of heterochromatin protein Rhino to a subset of piRNA clusters depends on a combination of two histone marks. Nat Struct Mol Biol 32:1517–1527 40527990 10.1038/s41594-025-01584-8 PMC12350163

[CR2] Alizada A, Hannon GJ, Nicholson BC (2025a) Transcriptional regulation of the piRNA pathway by Ovo in animal ovarian germ cells. Genes Dev 39:221–241 39797761 10.1101/gad.352120.124 PMC11789646

[CR3] Alizada A, Martins A, Mouniée N, Suarez JVR, Bertin B, Gueguen N, Mirouse V, Papameletiou A-M, Rivera AJ, Lau NC et al (2025b) The transcription factor Traffic jam orchestrates the somatic piRNA pathway in Drosophila ovaries. Cell Rep 44:115453 40209715 10.1016/j.celrep.2025.115453 PMC7618790

[CR4] Anand A, Kai T (2012) The tudor domain protein Kumo is required to assemble the nuage and to generate germline piRNAs in Drosophila. EMBO J 31:870–882 22157814 10.1038/emboj.2011.449 PMC3280549

[CR5] Anastasakis D, Skeparnias I, Shaukat A-N, Grafanaki K, Kanellou A, Taraviras S, Papachristou DJ, Papakyriakou A, Stathopoulos C (2016) Mammalian PNLDC1 is a novel poly(A) specific exonuclease with discrete expression during early development. Nucleic Acids Res 44:8908–8920 27515512 10.1093/nar/gkw709 PMC5062988

[CR6] Andersen PR, Tirian L, Vunjak M, Brennecke J (2017) A heterochromatin-dependent transcription machinery drives piRNA expression. Nature 549:54–59 28847004 10.1038/nature23482 PMC5590728

[CR7] Arnaiz O, Mathy N, Baudry C, Malinsky S, Aury J-M, Denby Wilkes C, Garnier O, Labadie K, Lauderdale BE, Le Mouël A et al (2012) The Paramecium germline genome provides a niche for intragenic parasitic DNA: evolutionary dynamics of internal eliminated sequences. PLoS Genet 8:e1002984 23071448 10.1371/journal.pgen.1002984 PMC3464196

[CR8] Balan T, Lerner LK, Holoch D, Duharcourt S (2024) Small-RNA-guided histone modifications and somatic genome elimination in ciliates. WIREs RNA 15:e1848 38605483 10.1002/wrna.1848

[CR9] Banani SF, Lee HO, Hyman AA, Rosen MK (2017) Biomolecular condensates: organizers of cellular biochemistry. Nat Rev Mol Cell Biol 18:285–298 28225081 10.1038/nrm.2017.7 PMC7434221

[CR10] Batki J, Schnabl J, Wang J, Handler D, Andreev VI, Stieger CE, Novatchkova M, Lampersberger L, Kauneckaite K, Xie W et al (2019) The nascent RNA binding complex SFiNX licenses piRNA-guided heterochromatin formation. Nat Struct Mol Biol 26:720–731 31384064 10.1038/s41594-019-0270-6 PMC6828549

[CR11] Baudry C, Malinsky S, Restituito M, Kapusta A, Rosa S, Meyer E, Bétermier M (2009) PiggyMac, a domesticated piggyBac transposase involved in programmed genome rearrangements in the ciliate *Paramecium tetraurelia*. Genes Dev 23:2478–2483 19884254 10.1101/gad.547309 PMC2779751

[CR12] Baumgartner L, Handler D, Platzer SW, Yu C, Duchek P, Brennecke J (2022) The Drosophila ZAD zinc finger protein Kipferl guides Rhino to piRNA clusters. eLife 11:e80067 36193674 10.7554/eLife.80067 PMC9531945

[CR13] Betermier M, Duharcourt S (2014) Programmed rearrangement in ciliates: Paramecium. Microbiol Spectr. 10.1128/microbiolspec.MDNA3-0035-2014 10.1128/microbiolspec.MDNA3-0035-2014 26104450

[CR14] Bischerour J, Bhullar S, Denby Wilkes C, Régnier V, Mathy N, Dubois E, Singh A, Swart E, Arnaiz O, Sperling L et al (2018) Six domesticated PiggyBac transposases together carry out programmed DNA elimination in Paramecium. eLife 7:e37927 30223944 10.7554/eLife.37927 PMC6143343

[CR15] Blumenstiel JP (2019) Birth, school, work, death, and resurrection: the life stages and dynamics of transposable element proliferation. Genes 10:336 31058854 10.3390/genes10050336 PMC6562965

[CR16] Bouhouche K, Gout J-F, Kapusta A, Bétermier M, Meyer E (2011) Functional specialization of Piwi proteins in *Paramecium tetraurelia* from post-transcriptional gene silencing to genome remodelling. Nucleic Acids Res 39:4249–4264 21216825 10.1093/nar/gkq1283 PMC3105430

[CR17] Bourque G, Burns KH, Gehring M, Gorbunova V, Seluanov A, Hammell M, Imbeault M, Izsvák Z, Levin HL, Macfarlan TS et al (2018) Ten things you should know about transposable elements. Genome Biol 19:199 30454069 10.1186/s13059-018-1577-z PMC6240941

[CR18] Brasset E, Taddei AR, Arnaud F, Faye B, Fausto AM, Mazzini M, Giorgi F, Vaury C (2006) Viral particles of the endogenous retrovirus ZAM from *Drosophila melanogaster* use a pre-existing endosome/exosome pathway for transfer to the oocyte. Retrovirology 3:25 16684341 10.1186/1742-4690-3-25 PMC1524798

[CR19] Brégnard C, Guerra J, Déjardin S, Passalacqua F, Benkirane M, Laguette N (2016) Upregulated LINE-1 activity in the *Fanconi anemia* cancer susceptibility syndrome leads to spontaneous pro-inflammatory cytokine production. EBioMedicine 8:184–194 27428429 10.1016/j.ebiom.2016.05.005 PMC4919473

[CR20] Brennecke J, Aravin AA, Stark A, Dus M, Kellis M, Sachidanandam R, Hannon GJ (2007) Discrete small RNA-generating loci as master regulators of transposon activity in Drosophila. Cell 128:1089–1103 17346786 10.1016/j.cell.2007.01.043

[CR21] Brookfield JFY (2005) The ecology of the genome—mobile DNA elements and their hosts. Nat Rev Genet 6:128–136 15640810 10.1038/nrg1524

[CR22] Capy P (2021) Taming, domestication and exaptation: trajectories of transposable elements in genomes. Cells 10:3590 34944100 10.3390/cells10123590 PMC8700633

[CR23] Carter TA, Singh M, Dumbović G, Chobirko JD, Rinn JL, Feschotte C (2022) Mosaic cis-regulatory evolution drives transcriptional partitioning of HERVH endogenous retrovirus in the human embryo. eLife 11:e76257 35179489 10.7554/eLife.76257 PMC8912925

[CR24] Casacuberta E (2017) Drosophila: retrotransposons making up telomeres. Viruses 9:192 28753967 10.3390/v9070192 PMC5537684

[CR25] Chabot BJ, Sun R, Amjad A, Hoyt SJ, Ouyang L, Courret C, Drennan R, Leo L, Larracuente AM, Core LJ et al (2024) Transcription of a centromere-enriched retroelement and local retention of its RNA are significant features of the CENP-A chromatin landscape. Genome Biol 25:295 39558354 10.1186/s13059-024-03433-1 PMC11575011

[CR26] Chakrabarty P, Sen R, Sengupta S (2023) From parasites to partners: exploring the intricacies of host-transposon dynamics and coevolution. Funct Integr Genomics 23:1–24 10.1007/s10142-023-01206-w 37610667

[CR27] Chalker DL, Meyer E, Mochizuki K (2013) Epigenetics of ciliates. Cold Spring Harb Perspect Biol 5:a017764 24296171 10.1101/cshperspect.a017764 PMC3839606

[CR28] Chalvet F, Teysset L, Terzian C, Prud’homme N, Santamaria P, Bucheton A, Pélisson A (1999) Proviral amplification of the Gypsy endogenous retrovirus of Drosophila melanogaster involves env-independent invasion of the female germline. EMBO J 18:2659–2669 10228177 10.1093/emboj/18.9.2659 PMC1171345

[CR29] Chang N-C, Wells JN, Wang AY, Schofield P, Huang Y-C, Truong VH, Simoes-Costa M, Feschotte C (2025) Gag proteins encoded by endogenous retroviruses are required for zebrafish development. Proc Natl Acad Sci USA 122:e2411446122 40294259 10.1073/pnas.2411446122 PMC12067270

[CR30] Chang TH, Mattei E, Gainetdinov I, Colpan C, Weng Z, Zamore PD (2019) Maelstrom represses canonical polymerase II transcription within bi-directional piRNA clusters in *Drosophila melanogaster*. Mol Cell 73:291–303.e6 30527661 10.1016/j.molcel.2018.10.038 PMC6551610

[CR31] Charlesworth B (1994) Evolution. How does increased fitness evolve? Curr Biol CB 4:1146–1148 7704584 10.1016/s0960-9822(00)00259-1

[CR32] Charmant O, Gruchota J, Arnaiz O, Nowak KP, Moisan N, Zangarelli C, Bétermier M, Anielska-Mazur A, Legros V, Chevreux G et al (2025) The PIWI-interacting protein Gtsf1 controls the selective degradation of small RNAs in Paramecium. Nucleic Acids Res 53:gkae1055 39571614 10.1093/nar/gkae1055 PMC11724296

[CR33] Chary S, Hayashi R (2023) The absence of core piRNA biogenesis factors does not impact efficient transposon silencing in Drosophila. PLoS Biol 21:e3002099 37279192 10.1371/journal.pbio.3002099 PMC10243637

[CR34] Chen Y-CA, Stuwe E, Luo Y, Ninova M, Le Thomas A, Rozhavskaya E, Li S, Vempati S, Laver JD, Patel DJ et al (2016) Cutoff suppresses RNA polymerase II termination to ensure expression of piRNA precursors. Mol Cell 63:97–109 27292797 10.1016/j.molcel.2016.05.010 PMC4980073

[CR35] Cheng C-Y, Vogt A, Mochizuki K, Yao M-C (2010) A domesticated piggyBac transposase plays key roles in heterochromatin dynamics and DNA cleavage during programmed DNA deletion in *Tetrahymena thermophila*. Mol Biol Cell 21:1753–1762 20357003 10.1091/mbc.E09-12-1079 PMC2869380

[CR36] Cheung S, Ma L, Chan PHW, Hu H-L, Mayor T, Chen H-T, Measday V (2016) Ty1 integrase interacts with RNA polymerase III-specific subcomplexes to promote insertion of Ty1 elements upstream of polymerase (Pol) III-transcribed genes. J Biol Chem 291:6396–6411 26797132 10.1074/jbc.M115.686840 PMC4813574

[CR37] Choi JY, Lee YCG (2020) Double-edged sword: the evolutionary consequences of the epigenetic silencing of transposable elements. PLoS Genet 16:e1008872 32673310 10.1371/journal.pgen.1008872 PMC7365398

[CR38] Choi S, Lee J-M, Kim KK (2025) Biomolecular condensates: molecular structure, biological functions, diseases, and therapeutic targets. Mol Biomed 6:99 41191214 10.1186/s43556-025-00350-y PMC12589764

[CR39] Choudhary MNK, Quaid K, Xing X, Schmidt H, Wang T (2023) Widespread contribution of transposable elements to the rewiring of mammalian 3D genomes. Nat Commun 14:634 36746940 10.1038/s41467-023-36364-9 PMC9902604

[CR40] Chung W-J, Okamura K, Martin R, Lai EC (2008) Endogenous RNA interference provides a somatic defense against Drosophila transposons. Curr Biol 18:795–802 18501606 10.1016/j.cub.2008.05.006 PMC2812477

[CR41] Cosby RL, Chang N-C, Feschotte C (2019) Host–transposon interactions: conflict, cooperation, and cooption. Genes Dev 33:1098–1116 31481535 10.1101/gad.327312.119 PMC6719617

[CR42] Coux R-X, Teixeira FK, Lehmann R (2018) L(3)mbt and the LINT complex safeguard cellular identity in the Drosophila ovary. Development 145:dev160721 29511022 10.1242/dev.160721 PMC5963868

[CR43] Czech B, Hannon GJ (2016) A happy 3′ ending to the piRNA maturation story. Cell 164:838–840 26919421 10.1016/j.cell.2016.02.012

[CR44] Czech B, Munafò M, Ciabrelli F, Eastwood EL, Fabry MH, Kneuss E, Hannon GJ (2018) piRNA-guided genome defense: from biogenesis to silencing. Annu Rev Genet 52:131–157 30476449 10.1146/annurev-genet-120417-031441 PMC10784713

[CR45] De D, Sarkar S, Gebert LFR, Wiryaman T, Anzelon TA, MacRae IJ (2025) A conserved PIWI silencing complex detects piRNA-target engagement. Mol Cell 85:3275–3287.e7 40912244 10.1016/j.molcel.2025.08.010 PMC12416740

[CR46] de la Rosa S, del Mar Rigual M, Vargiu P, Ortega S, Djouder N (2024) Endogenous retroviruses shape pluripotency specification in mouse embryos. Sci Adv 10:eadk9394 38266080 10.1126/sciadv.adk9394 PMC10807815

[CR47] Dennis C, Brasset E, Sarkar A, Vaury C (2016) Export of piRNA precursors by EJC triggers assembly of cytoplasmic Yb-body in Drosophila. Nat Commun 7:13739 27929060 10.1038/ncomms13739 PMC5155165

[CR48] Ding D, Liu J, Dong K, Midic U, Hess RA, Xie H, Demireva EY, Chen C (2017) PNLDC1 is essential for piRNA 3’ end trimming and transposon silencing during spermatogenesis in mice. Nat Commun 8:819 29018194 10.1038/s41467-017-00854-4 PMC5635004

[CR49] Donertas D, Sienski G, Brennecke J (2013) Drosophila Gtsf1 is an essential component of the Piwi-mediated transcriptional silencing complex. Genes Dev 27:1693–1705 23913922 10.1101/gad.221150.113 PMC3744727

[CR50] Dubois E, Bischerour J, Marmignon A, Mathy N, Régnier V, Bétermier M (2012) Transposon invasion of the paramecium germline genome countered by a domesticated PiggyBac transposase and the NHEJ pathway. Int J Evol Biol 2012:436196 22888464 10.1155/2012/436196 PMC3408717

[CR51] Dufourt J, Dennis C, Boivin A, Gueguen N, Théron E, Goriaux C, Pouchin P, Ronsseray S, Brasset E, Vaury C (2014) Spatio-temporal requirements for transposable element piRNA-mediated silencing during *Drosophila* oogenesis. Nucleic Acids Res 42:2512–2524 24288375 10.1093/nar/gkt1184 PMC3936749

[CR52] Eastwood EL, Jara KA, Bornelöv S, Munafò M, Frantzis V, Kneuss E, Barbar EJ, Czech B, Hannon GJ (2021) Dimerisation of the PICTS complex via LC8/Cut-up drives co-transcriptional transposon silencing in Drosophila. eLife 10:e65557 33538693 10.7554/eLife.65557 PMC7861614

[CR53] ElMaghraby MF, Andersen PR, Pühringer F, Hohmann U, Meixner K, Lendl T, Tirian L, Brennecke J (2019) A heterochromatin-specific RNA export pathway facilitates piRNA production. Cell 178:964–979.e20 31398345 10.1016/j.cell.2019.07.007

[CR235] Evgen’ev MB, Arkhipova IR (2005) Penelope-like elements--a new class of retroelements: distribution, function and possible evolutionary significance. Cytogenet Genome Res 110:510–521 10.1159/000084984 16093704

[CR54] Fabry MH, Ciabrelli F, Munafò M, Eastwood EL, Kneuss E, Falciatori I, Falconio FA, Hannon GJ, Czech B (2019) piRNA-guided co-transcriptional silencing coopts nuclear export factors. eLife 8:e47999 31219034 10.7554/eLife.47999 PMC6677536

[CR55] Fabry MH, Falconio FA, Joud F, Lythgoe EK, Czech B, Hannon GJ (2021) Maternally inherited piRNAs direct transient heterochromatin formation at active transposons during early Drosophila embryogenesis. eLife 10:e68573 34236313 10.7554/eLife.68573 PMC8352587

[CR56] Fang W, Wang X, Bracht JR, Nowacki M, Landweber LF (2012) Piwi-interacting RNAs protect DNA against loss during Oxytricha genome rearrangement. Cell 151:1243–1255 23217708 10.1016/j.cell.2012.10.045 PMC3678556

[CR236] Fattash I, Rooke R, Wong A, Hui C, Luu T, Bhardwaj P, Yang G (2013) Miniature inverted-repeat transposable elements: discovery, distribution, and activity. Genome 56:475–486 10.1139/gen-2012-0174 24168668

[CR57] Feltzin VL, Khaladkar M, Abe M, Parisi M, Hendriks G-J, Kim J, Bonini NM (2015) The exonuclease Nibbler regulates age-associated traits and modulates piRNA length in Drosophila. Aging Cell 14:443–452 25754031 10.1111/acel.12323 PMC4406673

[CR58] Feschotte C (2008) Transposable elements and the evolution of regulatory networks. Nat Rev Genet 9:397–405 18368054 10.1038/nrg2337 PMC2596197

[CR59] Fueyo R, Judd J, Feschotte C, Wysocka J (2022) Roles of transposable elements in the regulation of mammalian transcription. Nat Rev Mol Cell Biol 23:481–497. 1–17 35228718 10.1038/s41580-022-00457-y PMC10470143

[CR60] Gan H, Lin X, Zhang Z, Zhang W, Liao S, Wang L, Han C (2011) piRNA profiling during specific stages of mouse spermatogenesis. RNA 17:1191–1203 10.1261/rna.2648411 PMC3138557 21602304

[CR61] Gao Y, Solberg T, Wang C, Gao F (2023) Small RNA-mediated genome rearrangement pathways in ciliates. Trends Genet TIG 39:94–97 36371355 10.1016/j.tig.2022.10.001

[CR62] Ge DT, Wang W, Tipping C, Gainetdinov I, Weng Z, Zamore PD (2019) The RNA-binding ATPase, armitage, couples piRNA amplification in nuage to phased piRNA production on mitochondria. Mol Cell 74:982–995.e6 31076285 10.1016/j.molcel.2019.04.006 PMC6636356

[CR63] Ghanim GE, Rio DC, Teixeira FK (2020) Mechanism and regulation of P element transposition. Open Biol 10:200244 33352068 10.1098/rsob.200244 PMC7776569

[CR64] Ghildiyal M, Seitz H, Horwich MD, Li C, Du T, Lee S, Xu J, Kittler ELW, Zapp ML, Weng Z et al (2008) Endogenous siRNAs derived from transposons and mRNAs in Drosophila somatic cells. Science 320:1077–1081 18403677 10.1126/science.1157396 PMC2953241

[CR65] Gilboa L, Lehmann R (2006) Soma-germline interactions coordinate homeostasis and growth in the Drosophila gonad. Nature 443:97–100 16936717 10.1038/nature05068

[CR66] Girard A, Sachidanandam R, Hannon GJ, Carmell MA (2006) A germline-specific class of small RNAs binds mammalian Piwi proteins. Nature 442:199–202 16751776 10.1038/nature04917

[CR67] Goriaux C, Desset S, Renaud Y, Vaury C, Brasset E (2014) Transcriptional properties and splicing of the flamenco piRNA cluster. EMBO Rep 15:411–418 24562610 10.1002/embr.201337898 PMC3989672

[CR237] Goodwin TJD, Butler MI, Poulter RTM (2003) Cryptons: a group of tyrosine-recombinase-encoding DNA transposons from pathogenic fungi. Microbiology 149:3099–3109 10.1099/mic.0.26529-0 14600222

[CR68] Grow EJ, Flynn RA, Chavez SL, Bayless NL, Wossidlo M, Wesche DJ, Martin L, Ware CB, Blish CA, Chang HY et al (2015) Intrinsic retroviral reactivation in human preimplantation embryos and pluripotent cells. Nature 522:221–225 25896322 10.1038/nature14308 PMC4503379

[CR69] Gunawardane LS, Saito K, Nishida KM, Miyoshi K, Kawamura Y, Nagami T, Siomi H, Siomi MC (2007) A slicer-mediated mechanism for repeat-associated siRNA 5[prime] end formation in Drosophila. Science 315:1587–1590 17322028 10.1126/science.1140494

[CR70] Haase AD, Ketting RF, Lai EC, van Rij RP, Siomi M, Svoboda P, van Wolfswinkel JC, Wu P-H (2024) PIWI-interacting RNAs: who, what, when, where, why, and how. EMBO J 43:5335–5339 39327528 10.1038/s44318-024-00253-8 PMC11574264

[CR71] Hamilton EP, Kapusta A, Huvos PE, Bidwell SL, Zafar N, Tang H, Hadjithomas M, Krishnakumar V, Badger JH, Caler EV et al (2016) Structure of the germline genome of *Tetrahymena thermophila* and relationship to the massively rearranged somatic genome. eLife 5:e19090 27892853 10.7554/eLife.19090 PMC5182062

[CR72] Handler D, Meixner K, Pizka M, Lauss K, Schmied C, Gruber FS, Brennecke J (2013) The genetic makeup of the Drosophila piRNA pathway. Mol Cell 50:762–777 23665231 10.1016/j.molcel.2013.04.031 PMC3679447

[CR73] Handler D, Olivieri D, Novatchkova M, Gruber FS, Meixner K, Mechtler K, Stark A, Sachidanandam R, Brennecke J (2011) A systematic analysis of Drosophila TUDOR domain-containing proteins identifies Vreteno and the Tdrd12 family as essential primary piRNA pathway factors. EMBO J 30:3977–3993 21863019 10.1038/emboj.2011.308 PMC3209783

[CR238] Havecker ER, Gao X, Voytas DF (2004) The diversity of LTR retrotransposons. Genome Biol 5:1–6 10.1186/gb-2004-5-6-225 PMC463057 15186483

[CR74] Haws SA, Simandi Z, Barnett RJ, Phillips-Cremins JE (2022) 3D genome, on repeat: Higher-order folding principles of the heterochromatinized repetitive genome. Cell 185:2690–2707 35868274 10.1016/j.cell.2022.06.052 PMC10225251

[CR75] Hayashi R, Schnabl J, Handler D, Mohn F, Ameres SL, Brennecke J (2016) Genetic and mechanistic diversity of piRNA 3′-end formation. Nature 539:588–592 10.1038/nature20162 PMC5164936 27851737

[CR76] Hirakata S, Fujita A, Siomi MC (2026) Transient residence of the repulsive client shutdown in Yb bodies plays a critical role in Piwi-piRISC biogenesis and maintaining fertility. Mol Cell 86:1345–1361 10.1016/j.molcel.2026.03.003 41932311

[CR77] Hirakata S, Ishizu H, Fujita A, Tomoe Y, Siomi MC (2019) Requirements for multivalent Yb body assembly in transposon silencing in Drosophila. EMBO Rep 20:e47708 31267711 10.15252/embr.201947708 PMC6607011

[CR78] Horwich MD, Li C, Matranga C, Vagin V, Farley G, Wang P, Zamore PD (2007) The Drosophila RNA methyltransferase, DmHen1, modifies germline piRNAs and single-stranded siRNAs in RISC. Curr Biol CB 17:1265–1272 17604629 10.1016/j.cub.2007.06.030

[CR79] Hutvagner G, Simard MJ (2008) Argonaute proteins: key players in RNA silencing. Nat Rev Mol Cell Biol 9:22–32 18073770 10.1038/nrm2321

[CR80] Ipsaro JJ, Haase AD, Knott SR, Joshua-Tor L, Hannon GJ (2012) The structural biochemistry of Zucchini implicates it as a nuclease in piRNA biogenesis. Nature 491:279–283 23064227 10.1038/nature11502 PMC3493678

[CR81] Ishizu H, Kinoshita T, Hirakata S, Komatsuzaki C, Siomi MC (2019) Distinct and collaborative functions of Yb and armitage in transposon-targeting piRNA biogenesis. Cell Rep 27:1822–1835.e8 31067466 10.1016/j.celrep.2019.04.029

[CR82] Iwasaki YW, Murano K, Ishizu H, Shibuya A, Iyoda Y, Siomi MC, Siomi H, Saito K (2016) Piwi modulates chromatin accessibility by regulating multiple factors including histone H1 to repress transposons. Mol Cell 63:408–419 27425411 10.1016/j.molcel.2016.06.008

[CR83] Jachowicz JW, Bing X, Pontabry J, Bošković A, Rando OJ, Torres-Padilla M-E (2017) LINE-1 activation after fertilization regulates global chromatin accessibility in the early mouse embryo. Nat Genet 49:1502–1510 28846101 10.1038/ng.3945

[CR84] Jansen G, Gebert D, Kumar TR, Simmons E, Murphy S, Teixeira FK (2024) Tolerance thresholds underlie responses to DNA damage during germline development. Genes Dev 38:631–654 39054057 10.1101/gad.351701.124 PMC11368186

[CR85] Jensen PA, Stuart JR, Goodpaster MP, Goodman JW, Simmons MJ (2008) Cytotype regulation of P transposable elements in *Drosophila melanogaster*: repressor polypeptides or piRNAs? Genetics 179:1785–1793 18579507 10.1534/genetics.108.087072 PMC2516058

[CR86] Juliano C, Wang J, Lin H (2011) Uniting germline and stem cells: the function of Piwi proteins and the piRNA pathway in diverse organisms. Annu Rev Genet 45:447–469 21942366 10.1146/annurev-genet-110410-132541 PMC3832951

[CR245] Kapitonov VV, Jurka J (2007) Helitrons on a roll: eukaryotic rolling-circle transposons. Trends Genet 23:521–529 10.1016/j.tig.2007.08.004 17850916

[CR87] Kawaguchi S, Isshiki W, Kai T (2025) Factories without walls: the molecular architecture and functions of non-membrane organelles in small RNA-guided genome protection. Biochim Biophys Acta BBA - Gen Subj 1869:130811 10.1016/j.bbagen.2025.130811 40319768

[CR88] Ke N (1997) The pheromone response pathway activates transcription of Ty5 retrotransposons located within silent chromatin of *Saccharomyces cerevisiae*. EMBO J 16:6272–6280 9321406 10.1093/emboj/16.20.6272 PMC1326311

[CR89] Kelleher ES, Jaweria J, Akoma U, Ortega L, Tang W (2018) QTL mapping of natural variation reveals that the developmental regulator bruno reduces tolerance to P-element transposition in the Drosophila female germline. PLoS Biol 16:e2006040 30376574 10.1371/journal.pbio.2006040 PMC6207299

[CR90] Khurana JS, Wang J, Xu J, Koppetsch BS, Thomson TC, Nowosielska A, Li C, Zamore PD, Weng Z, Theurkauf WE (2011) Adaptation to P element transposon invasion in *Drosophila melanogaster*. Cell 147:1551–1563 22196730 10.1016/j.cell.2011.11.042 PMC3246748

[CR91] Kinsey PT, Sandmeyer SB (1995) Ty3 transposes in mating populations of yeast: a novel transposition assay for Ty3. Genetics 139:81–94 7705653 10.1093/genetics/139.1.81 PMC1206350

[CR92] Kirchner J, Connolly CM, Sandmeyer SB (1995) Requirement of RNA polymerase III transcription factors for in vitro position-specific integration of a retroviruslike element. Science 267:1488–1491 7878467 10.1126/science.7878467

[CR93] Kirino Y, Vourekas A, Sayed N, Alves F, de L, Thomson T, Lasko P, Rappsilber J, Jongens TA, Mourelatos Z (2010) Arginine methylation of Aubergine mediates Tudor binding and germ plasm localization. RNA 16:70–78 19926723 10.1261/rna.1869710 PMC2802038

[CR94] Klattenhoff C, Xi H, Li C, Lee S, Xu J, Khurana JS, Zhang F, Schultz N, Koppetsch BS, Nowosielska A et al (2009) The Drosophila HP1 homolog Rhino is required for transposon silencing and piRNA production by dual-strand clusters. Cell 138:1137–1149 19732946 10.1016/j.cell.2009.07.014 PMC2770713

[CR95] Klumpe S, Senti KA, Beck F, Sachweh J, Hampoelz B, Ronchi P, Oorschot V, Brandstetter M, Yeroslaviz A, Briggs JAG et al (2025) In-cell structure and snapshots of copia retrotransposons in intact tissue by cryoelectron tomography. Cell 188:2094–2110 40049165 10.1016/j.cell.2025.02.003

[CR96] Kneuss E, Munafò M, Eastwood EL, Deumer U-S, Preall JB, Hannon GJ, Czech B (2019) Specialization of the Drosophila nuclear export family protein Nxf3 for piRNA precursor export. Genes Dev 33:1208–1220 31416967 10.1101/gad.328690.119 PMC6719614

[CR97] Koch CM, Honemann-Capito M, Egger-Adam D, Wodarz A (2009) Windei, the Drosophila homolog of mAM/MCAF1, is an essential cofactor of the H3K9 methyl transferase dSETDB1/eggless in germ line development. PLoS Genet 5:e1000644 19750210 10.1371/journal.pgen.1000644 PMC2730569

[CR98] Kofler R, Senti K-A, Nolte V, Tobler R, Schlötterer C (2018) Molecular dissection of a natural transposable element invasion. Genome Res 28:824–835 29712752 10.1101/gr.228627.117 PMC5991514

[CR99] Koga Y, Hirakata S, Negishi M, Yamazaki H, Fujisawa T, Siomi MC (2024) Dipteran-specific Daedalus controls Zucchini endonucleolysis in piRNA biogenesis independent of exonucleases. Cell Rep 43:114923 39487988 10.1016/j.celrep.2024.114923

[CR100] Kojima KK, Seto Y, Fujiwara H (2016) The wide distribution and change of target specificity of R2 non-LTR retrotransposons in animals. PLoS ONE 11:e0163496 27662593 10.1371/journal.pone.0163496 PMC5035012

[CR239] Kramerov DA, Vassetzky NS (2011) Origin and evolution of SINEs in eukaryotic genomes. Heredity 107:487–495 10.1038/hdy.2011.43 PMC3242629 21673742

[CR101] Kunert N, Wagner E, Murawska M, Klinker H, Kremmer E, Brehm A (2009) dMec: a novel Mi-2 chromatin remodelling complex involved in transcriptional repression. EMBO J 28:533–544 19165147 10.1038/emboj.2009.3 PMC2657585

[CR102] Larouche J-D, Laumont CM, Trofimov A, Vincent K, Hesnard L, Brochu S, Côté C, Humeau JF, Bonneil É, Lanoix J et al (2024) Transposable elements regulate thymus development and function. eLife 12:RP91037 38635416 10.7554/eLife.91037 PMC11026094

[CR103] Laski FA, Rio DC, Rubin GM (1986) Tissue specificity of Drosophila P element transposition is regulated at the level of mRNA splicing. Cell 44:7–19 3000622 10.1016/0092-8674(86)90480-0

[CR104] Lawlor MA, Cao W, Ellison CE (2021) A transposon expression burst accompanies the activation of Y-chromosome fertility genes during Drosophila spermatogenesis. Nat Commun 12:6854 34824217 10.1038/s41467-021-27136-4 PMC8617248

[CR105] Lawlor MA, Ellison CE (2023) Evolutionary dynamics between transposable elements and their host genomes: mechanisms of suppression and escape. Curr Opin Genet Dev 82:102092 37517354 10.1016/j.gde.2023.102092 PMC10530431

[CR106] Le Rouzic A, Dupas S, Capy P (2007) Genome ecosystem and transposable elements species. Gene 390:214–220 17188821 10.1016/j.gene.2006.09.023

[CR107] Le Thomas A, Rogers AK, Webster A, Marinov GK, Liao SE, Perkins EM, Hur JK, Aravin AA, Toth KF (2013) Piwi induces piRNA-guided transcriptional silencing and establishment of a repressive chromatin state. Genes Dev 27:390–399 23392610 10.1101/gad.209841.112 PMC3589556

[CR108] Le Thomas A, Stuwe E, Li S, Du J, Marinov G, Rozhkov N, Chen Y-CA, Luo Y, Sachidanandam R, Toth KF et al (2014) Transgenerationally inherited piRNAs trigger piRNA biogenesis by changing the chromatin of piRNA clusters and inducing precursor processing. Genes Dev 28:1667–1680 25085419 10.1101/gad.245514.114 PMC4117942

[CR109] Lécher P, Bucheton A, Pélisson A (1997) Expression of the Drosophila retrovirus gypsy as ultrastructurally detectable particles in the ovaries of flies carrying a permissive flamenco allele. J Gen Virol 78(Pt 9):2379–2388 9292028 10.1099/0022-1317-78-9-2379

[CR110] Lee YCG, Langley CH (2012) Long-term and short-term evolutionary impacts of transposable elements on Drosophila. Genetics 192:1411–1432 22997235 10.1534/genetics.112.145714 PMC3512147

[CR111] Lehner PJ (2025) Silencing by the HUSH epigenetic transcriptional repressor complex. Annu Rev Biochem 94:361–38640540752 10.1146/annurev-biochem-020425-045352

[CR112] Li S, Shen X (2023) Long interspersed nuclear element 1 and B1/Alu repeats blueprint genome compartmentalization. Curr Opin Genet Dev 80:102049 37229928 10.1016/j.gde.2023.102049

[CR240] Li X, Liu N (2025) Advances in understanding LINE-1 regulation and function in the human genome. Trends Genet 41:577–589 10.1016/j.tig.2025.04.011 40382218

[CR113] Lim AK, Kai T (2007) Unique germ-line organelle, nuage, functions to repress selfish genetic elements in Drosophila melanogaster. Proc Natl Acad Sci USA 104:6714–6719 17428915 10.1073/pnas.0701920104 PMC1871851

[CR114] Lin Y, Suyama R, Kawaguchi S, Iki T, Kai T (2023) Tejas functions as a core component in nuage assembly and precursor processing in Drosophila piRNA biogenesis. J Cell Biol 222:e202303125 37555815 10.1083/jcb.202303125 PMC10412688

[CR115] Liu L, Qi H, Wang J, Lin H (2011) PAPI, a novel Tudor-domain protein, complexes with AGO3, Me31b and Tral in the nuage to silence transposition. Development 138:1863–1873 10.1242/dev.059287 PMC3074456 21447556

[CR116] Lokta AJ (1925) Elements of physical biology. Nature 116:461–461

[CR117] Lu J, Clark AG (2010) Population dynamics of PIWI-interacting RNAs (piRNAs) and their targets in Drosophila. Genome Res 20:212–227 19948818 10.1101/gr.095406.109 PMC2813477

[CR118] Malik HS, Henikoff S, Eickbush TH (2000) Poised for contagion: evolutionary origins of the infectious abilities of invertebrate retroviruses. Genome Res 10:1307–1318 10984449 10.1101/gr.145000

[CR119] Malone CD, Anderson AM, Motl JA, Rexer CH, Chalker DL (2005) Germ line transcripts are processed by a Dicer-like protein that is essential for developmentally programmed genome rearrangements of *Tetrahymena thermophila*. Mol Cell Biol 25:9151–9164 16199890 10.1128/MCB.25.20.9151-9164.2005 PMC1265777

[CR120] Malone CD, Brennecke J, Dus M, Stark A, McCombie WR, Sachidanandam R, Hannon GJ (2009) Specialized piRNA pathways act in germline and somatic tissues of the Drosophila ovary. Cell 137:522–535 19395010 10.1016/j.cell.2009.03.040 PMC2882632

[CR121] M’Angale PG, Lemieux A, Liu Y, Wang S, Zinter M, Alegre G, Simkin A, Budnik V, Kelch BA, Thomson T (2025) Capsid transfer of the retrotransposon Copia controls structural synaptic plasticity in Drosophila. PLoS Biol 23:e3002983 39964983 10.1371/journal.pbio.3002983 PMC11835246

[CR122] Mathavarajah S, Dellaire G (2024) LINE-1: an emerging initiator of cGAS-STING signalling and inflammation that is dysregulated in disease. Biochem Cell Biol 102:38–46 37643478 10.1139/bcb-2023-0134

[CR123] Mével-Ninio M, Pelisson A, Kinder J, Campos AR, Bucheton A (2007) The flamenco locus controls the gypsy and ZAM retroviruses and is required for Drosophila oogenesis. Genetics 175:1615–1624 17277359 10.1534/genetics.106.068106 PMC1855114

[CR124] Miesen P, Joosten J, van Rij RP (2016) PIWIs go viral: arbovirus-derived piRNAs in vector mosquitoes. PLoS Pathog 12:e1006017 28033427 10.1371/journal.ppat.1006017 PMC5198996

[CR125] Miró-Pina C, Charmant O, Kawaguchi T, Holoch D, Michaud A, Cohen I, Humbert A, Jaszczyszyn Y, Chevreux G, Del Maestro L et al (2022) Paramecium polycomb repressive complex 2 physically interacts with the small RNA-binding PIWI protein to repress transposable elements. Dev Cell 57:1037–1052.e8 35429435 10.1016/j.devcel.2022.03.014

[CR126] Mochizuki K, Fine NA, Fujisawa T, Gorovsky MA (2002) Analysis of a piwi-related gene implicates small RNAs in genome rearrangement in tetrahymena. Cell 110:689–699 12297043 10.1016/s0092-8674(02)00909-1

[CR127] Mochizuki K, Gorovsky MA (2005) A Dicer-like protein in Tetrahymena has distinct functions in genome rearrangement, chromosome segregation, and meiotic prophase. Genes Dev 19:77–89 15598983 10.1101/gad.1265105 PMC540227

[CR128] Mohn F, Sienski G, Handler D, Brennecke J (2014) The Rhino-Deadlock-Cutoff complex licenses noncanonical transcription of dual-strand piRNA clusters in Drosophila. Cell 157:1364–1379 24906153 10.1016/j.cell.2014.04.031

[CR129] Moon S, Cassani M, Lin YA, Wang L, Dou K, Zhang ZZ (2018) A robust transposon-endogenizing response from germline stem cells. Dev Cell 47:660–671 30393075 10.1016/j.devcel.2018.10.011 PMC6631374

[CR130] Moore RS, Kaletsky R, Lesnik C, Cota V, Blackman E, Parsons LR, Gitai Z, Murphy CT (2021) The role of the Cer1 transposon in horizontal transfer of transgenerational memory. Cell 184:4697–4712.e18 34363756 10.1016/j.cell.2021.07.022 PMC8812995

[CR131] Mugat B, Nicot S, Varela-Chavez C, Jourdan C, Sato K, Basyuk E, Juge F, Siomi MC, Pélisson A, Chambeyron S (2020) The Mi-2 nucleosome remodeler and the Rpd3 histone deacetylase are involved in piRNA-guided heterochromatin formation. Nat Commun 11:2818 32499524 10.1038/s41467-020-16635-5 PMC7272611

[CR132] Munafò M, Manelli V, Falconio FA, Sawle A, Kneuss E, Eastwood EL, Seah JWE, Czech B, Hannon GJ (2019) Daedalus and Gasz recruit Armitage to mitochondria, bringing piRNA precursors to the biogenesis machinery. Genes Dev 33:844–856 31123065 10.1101/gad.325662.119 PMC6601507

[CR133] Murano K, Iwasaki YW, Ishizu H, Mashiko A, Shibuya A, Kondo S, Adachi S, Suzuki S, Saito K, Natsume T et al (2019) Nuclear RNA export factor variant initiates piRNA-guided co-transcriptional silencing. EMBO J 38:e102870 31368590 10.15252/embj.2019102870 PMC6717896

[CR134] Namba Y, Iwasaki YW, Nishida KM, Nishihara H, Sumiyoshi T, Siomi MC (2022) Maelstrom functions in the production of Siwi-piRISC capable of regulating transposons in Bombyx germ cells. iScience 25:103914 35243263 10.1016/j.isci.2022.103914 PMC8881725

[CR135] Nelson JO, Slicko A, Yamashita YM (2023) The retrotransposon R2 maintains Drosophila ribosomal DNA repeats. Proc Natl Acad Sci USA 120:e2221613120 37252996 10.1073/pnas.2221613120 PMC10266012

[CR241] Nesmelova IV, Hackett PB (2010) DDE Transposases: Structural Similarity and Diversity. Adv Drug Deliv Rev 62:1187–1195 10.1016/j.addr.2010.06.006 PMC2991504 20615441

[CR136] Ninova M, Chen Y-CA, Godneeva B, Rogers AK, Luo Y, Fejes Tóth K, Aravin AA (2020) Su(var)2-10 and the SUMO pathway link piRNA-guided target recognition to chromatin silencing. Mol Cell 77:556–570.e6 31901446 10.1016/j.molcel.2019.11.012 PMC7007863

[CR137] Nishida KM, Iwasaki YW, Murota Y, Nagao A, Mannen T, Kato Y, Siomi H, Siomi MC (2015) Respective functions of two distinct Siwi complexes assembled during PIWI-interacting RNA biogenesis in Bombyx germ cells. Cell Rep 10:193–203 25558067 10.1016/j.celrep.2014.12.013

[CR138] Nishida KM, Okada TN, Kawamura T, Mituyama T, Kawamura Y, Inagaki S, Huang H, Chen D, Kodama T, Siomi H et al (2009) Functional involvement of Tudor and dPRMT5 in the piRNA processing pathway in Drosophila germlines. EMBO J 28:3820–3831 19959991 10.1038/emboj.2009.365 PMC2797066

[CR139] Nishida KM, Sakakibara K, Sumiyoshi T, Yamazaki H, Mannen T, Kawamura T, Kodama T, Siomi MC (2020) Siwi levels reversibly regulate secondary piRISC biogenesis by affecting Ago3 body morphology in *Bombyx mori*. EMBO J 39:e105130 32914505 10.15252/embj.2020105130 PMC7560202

[CR140] Nishimasu H, Ishizu H, Saito K, Fukuhara S, Kamatani MK, Bonnefond L, Matsumoto N, Nishizawa T, Nakanaga K, Aoki J et al (2012) Structure and function of Zucchini endoribonuclease in piRNA biogenesis. Nature 491:284–287 23064230 10.1038/nature11509

[CR141] Nishimura T, Nagamori I, Nakatani T, Izumi N, Tomari Y, Kuramochi-Miyagawa S, Nakano T (2018) PNLDC1, mouse pre-piRNA Trimmer, is required for meiotic and post-meiotic male germ cell development. EMBO Rep 19:e44957 29444933 10.15252/embr.201744957 PMC5836094

[CR142] Ohtani H, Iwasaki YW, Shibuya A, Siomi H, Siomi MC, Saito K (2013) DmGTSF1 is necessary for Piwi-piRISC-mediated transcriptional transposon silencing in the Drosophila ovary. Genes Dev 27:1656–1661 23913921 10.1101/gad.221515.113 PMC3744724

[CR143] Olivieri D, Senti K-A, Subramanian S, Sachidanandam R, Brennecke J (2012) The cochaperone shutdown defines a group of biogenesis factors essential for all piRNA populations in Drosophila. Mol Cell 47:954–969 22902557 10.1016/j.molcel.2012.07.021 PMC3463805

[CR144] Olivieri D, Sykora MM, Sachidanandam R, Mechtler K, Brennecke J (2010) An in vivo RNAi assay identifies major genetic and cellular requirements for primary piRNA biogenesis in Drosophila. EMBO J 29:3301–3317 20818334 10.1038/emboj.2010.212 PMC2957214

[CR145] Onishi R, Sato K, Murano K, Negishi L, Siomi H, Siomi MC (2020) Piwi suppresses transcription of Brahma-dependent transposons via Maelstrom in ovarian somatic cells. Sci Adv 6:eaaz7420 33310860 10.1126/sciadv.aaz7420 PMC7732180

[CR146] Osumi K, Sato K, Murano K, Siomi H, Siomi MC (2019) Essential roles of Windei and nuclear monoubiquitination of Eggless/SETDB1 in transposon silencing. EMBO Rep 20:e48296 31576653 10.15252/embr.201948296 PMC6893296

[CR147] Ozata DM, Gainetdinov I, Zoch A, O’Carroll D, Zamore PD (2019) PIWI-interacting RNAs: small RNAs with big functions. Nat Rev Genet 20:89–108 30446728 10.1038/s41576-018-0073-3

[CR148] Pane A, Wehr K, Schüpbach T (2007) Zucchini and squash encode two putative nucleases required for rasiRNA production in the Drosophila germline. Dev Cell 12:851–862 17543859 10.1016/j.devcel.2007.03.022 PMC1945814

[CR149] Parhad SS, Theurkauf WE (2019) Rapid evolution and conserved function of the piRNA pathway. Open Biol 9:180181 30958115 10.1098/rsob.180181 PMC6367137

[CR150] Pasyukova E, Nuzhdin S, Li W, Flavell AJ (1997) Germ line transposition of the copia retrotransposon in *Drosophila melanogaster* is restricted to males by tissue-specific control of copia RNA levels. Mol Gen Genet 255:115–124 9230904 10.1007/s004380050479

[CR151] Patil VS, Anand A, Chakrabarti A, Kai T (2014) The Tudor domain protein Tapas, a homolog of the vertebrate Tdrd7, functions in the piRNA pathway to regulate retrotransposons in germline of *Drosophila melanogaster*. BMC Biol 12:61 25287931 10.1186/s12915-014-0061-9 PMC4210518

[CR152] Patil VS, Kai T (2010) Repression of retroelements in Drosophila germline via piRNA pathway by the Tudor domain protein Tejas. Curr Biol CB 20:724–730 20362446 10.1016/j.cub.2010.02.046

[CR153] Payer LM, Burns KH (2019) Transposable elements in human genetic disease. Nat Rev Genet 20:760–772 31515540 10.1038/s41576-019-0165-8

[CR154] Pélisson A, Song SU, Prud’homme N, Smith PA, Bucheton A, Corces VG (1994) Gypsy transposition correlates with the production of a retroviral envelope-like protein under the tissue-specific control of the Drosophila flamenco gene. EMBO J 13:4401–4411 7925283 10.1002/j.1460-2075.1994.tb06760.x PMC395367

[CR155] Percharde M, Lin C-J, Yin Y, Guan J, Peixoto GA, Bulut-Karslioglu A, Biechele S, Huang B, Shen X, Ramalho-Santos M (2018) A LINE1-nucleolin partnership regulates early development and ESC identity. Cell 174:391–405.e19 29937225 10.1016/j.cell.2018.05.043 PMC6046266

[CR156] Pianezza R, Scarpa A, Haider A, Signor S, Kofler R (2025) Spatiotemporal tracking of three novel transposable element invasions in *Drosophila melanogaster* over the last 30 years. Mol Biol Evol 42:msaf143 40479505 10.1093/molbev/msaf143 PMC12230796

[CR157] Platt IIRN, Ray DA (2012) A non-LTR retroelement extinction in *Spermophilus tridecemlineatus*. Gene 500:47–53 22465530 10.1016/j.gene.2012.03.051

[CR158] Portell-Montserrat J, Tirian L, Yu C, Silvestri G, Hohmann U, Handler D, Duchek P, Fin L, Plaschka C, Brennecke J (2025) Target RNA recognition drives PIWI∗ complex assembly for transposon silencing. Mol Cell 85:3288–3305.e6 40912245 10.1016/j.molcel.2025.08.007

[CR242] Poulter RTM, Goodwin TJD (2005) DIRS-1 and the other tyrosine recombinase retrotransposons. Cytogenet Genome Res 110:575–588 10.1159/000084991 16093711

[CR159] Power M-A, Tam PPL (1993) Onset of gastrulation, morphogenesis and somitogenesis in mouse embryos displaying compensatory growth. Anat Embryol 187:493–504 10.1007/BF00174425 8342794

[CR160] Preall JB, Czech B, Guzzardo PM, Muerdter F, Hannon GJ (2012) Shutdown is a component of the Drosophila piRNA biogenesis machinery. RNA 18:1446–1457 22753781 10.1261/rna.034405.112 PMC3404366

[CR243] Pritham EJ, Putliwala T, Feschotte C (2007) Mavericks, a novel class of giant transposable elements widespread in eukaryotes and related to DNA viruses. Gene 390:3–17 10.1016/j.gene.2006.08.008 17034960

[CR161] Qi H, Watanabe T, Ku HY, Liu N, Zhong M, Lin H (2011) The Yb body, a major site for Piwi-associated RNA biogenesis and a gateway for Piwi expression and transport to the nucleus in somatic cells. J Biol Chem 286:3789–3797 21106531 10.1074/jbc.M110.193888 PMC3030380

[CR162] Rangan P, Malone CD, Navarro C, Newbold SP, Hayes PS, Sachidanandam R, Hannon GJ, Lehmann R (2011) piRNA production requires heterochromatin formation in Drosophila. Curr Biol 21:1373–1379 21820311 10.1016/j.cub.2011.06.057 PMC3205116

[CR163] Rivera AJ, Lee J-HR, Gupta S, Yang L, Goel RK, Zaia J, Lau NC (2025) Traffic Jam activates the Flamenco piRNA cluster locus and the Piwi pathway to ensure transposon silencing and Drosophila fertility. Cell Rep 44:115354 40209716 10.1016/j.celrep.2025.115354 PMC12094058

[CR164] Robillard É, Le Rouzic A, Zhang Z, Capy P, Hua-Van A (2016) Experimental evolution reveals hyperparasitic interactions among transposable elements. Proc Natl Acad Sci USA 113:14763–14768 27930288 10.1073/pnas.1524143113 PMC5187678

[CR165] Roche SE, Schiff M, Rio DC (1995) P-element repressor autoregulation involves germ-line transcriptional repression and reduction of third intron splicing. Genes Dev 9:1278–1288 7758951 10.1101/gad.9.10.1278

[CR166] Rohrmann GF, Karplus PA (2001) Relatedness of baculovirus and gypsy retrotransposon envelope proteins. BMC Evol Biol 1:1 11244578 10.1186/1471-2148-1-1 PMC29073

[CR167] Rosspopoff O, Trono D (2023) Take a walk on the KRAB side. Trends Genet TIG 39:844–857 37716846 10.1016/j.tig.2023.08.003

[CR168] Roth S (2001) Drosophila oogenesis: coordinating germ line and soma. Curr Biol 11:R779–R781 11591336 10.1016/s0960-9822(01)00469-9

[CR169] Rowe HM, Jakobsson J, Mesnard D, Rougemont J, Reynard S, Aktas T, Maillard PV, Layard-Liesching H, Verp S, Marquis J et al (2010) KAP1 controls endogenous retroviruses in embryonic stem cells. Nature 463:237–240 20075919 10.1038/nature08674

[CR170] Rozhkov NV, Hammell M, Hannon GJ (2013) Multiple roles for Piwi in silencing Drosophila transposons. Genes Dev 27:400–412 23392609 10.1101/gad.209767.112 PMC3589557

[CR171] Ryazansky SS, Kotov AA, Kibanov MV, Akulenko NV, Korbut AP, Lavrov SA, Gvozdev VA, Olenina LV (2016) RNA helicase Spn-E is required to maintain Aub and AGO3 protein levels for piRNA silencing in the germline of *Drosophila*. Eur J Cell Biol 95:311–322 27320195 10.1016/j.ejcb.2016.06.001

[CR172] Saito K, Inagaki S, Mituyama T, Kawamura Y, Ono Y, Sakota E, Kotani H, Asai K, Siomi H, Siomi MC (2009) A regulatory circuit for piwi by the large Maf gene traffic jam in Drosophila. Nature 461:1296–1299 19812547 10.1038/nature08501

[CR173] Saito K, Ishizu H, Komai M, Kotani H, Kawamura Y, Nishida KM, Siomi H, Siomi MC (2010) Roles for the Yb body components Armitage and Yb in primary piRNA biogenesis in Drosophila. Genes Dev 24:2493–2498 20966047 10.1101/gad.1989510 PMC2975925

[CR174] Saito K, Nishida KM, Mori T, Kawamura Y, Miyoshi K, Nagami T, Siomi H, Siomi MC (2006) Specific association of Piwi with rasiRNAs derived from retrotransposon and heterochromatic regions in the Drosophila genome. Genes Dev 20:2214–2222 16882972 10.1101/gad.1454806 PMC1553205

[CR175] Saito K, Sakaguchi Y, Suzuki T, Suzuki T, Siomi H, Siomi MC (2007) Pimet, the Drosophila homolog of HEN1, mediates 2′-O-methylation of Piwi- interacting RNAs at their 3′ ends. Genes Dev 21:1603–1608 17606638 10.1101/gad.1563607 PMC1899469

[CR176] Saito R, Ishizu H, Harigai R, Murano K, Namba Y, Siomi MC (2025) Role of ADMA-histones in dual-strand piRNA source loci recognition by Rhino. eLife 14:RP107358

[CR177] Sakashita A, Kitano T, Ishizu H, Guo Y, Masuda H, Ariura M, Murano K, Siomi H (2023) Transcription of MERVL retrotransposons is required for preimplantation embryo development. Nat Genet 55:484–495 36864102 10.1038/s41588-023-01324-y PMC10011141

[CR178] Sato K, Iwasaki YW, Shibuya A, Carninci P, Tsuchizawa Y, Ishizu H, Siomi MC, Siomi H (2015) Krimper enforces an antisense bias on piRNA pools by binding AGO3 in the *Drosophila* Germline. Mol Cell 59:553–563 26212455 10.1016/j.molcel.2015.06.024

[CR179] Sato K, Siomi MC (2018) Two distinct transcriptional controls triggered by nuclear Piwi-piRISCs in the Drosophila piRNA pathway. Curr Opin Struct Biol 53:69–76 29990672 10.1016/j.sbi.2018.06.005

[CR180] Schnabl J, Wang J, Hohmann U, Gehre M, Batki J, Andreev VI, Purkhauser K, Fasching N, Duchek P, Novatchkova M et al (2021) Molecular principles of Piwi-mediated cotranscriptional silencing through the dimeric SFiNX complex. Genes Dev 35:392–409 33574069 10.1101/gad.347989.120 PMC7919418

[CR181] Schneider L, Guo Y, Birch D, Sarkies P (2021) Network-based visualisation reveals new insights into transposable element diversity. Mol Syst Biol 17:e9600 34169647 10.15252/msb.20209600 PMC8226279

[CR182] Schoeberl UE, Kurth HM, Noto T, Mochizuki K (2012) Biased transcription and selective degradation of small RNAs shape the pattern of DNA elimination in Tetrahymena. Genes Dev 26:1729–1742 22855833 10.1101/gad.196493.112 PMC3418590

[CR183] Seczynska M, Lehner PJ (2023) The sound of silence: mechanisms and implications of HUSH complex function. Trends Genet TIG 39:251–267 36754727 10.1016/j.tig.2022.12.005

[CR184] Senti K-A, Rafanel B, Handler D, Kosiol C, Schlötterer C, Brennecke J (2025) Co-evolving infectivity and expression patterns drive the diversification of endogenous retroviruses. EMBO J 45:1–20 10.1038/s44318-025-00471-8 PMC12992720 40474005

[CR185] Sienski G, Batki J, Senti K-A, Dönertas D, Tirian L, Meixner K, Brennecke J (2015) Silencio/CG9754 connects the Piwi-piRNA complex to the cellular heterochromatin machinery. Genes Dev 29:2258–2271 26494711 10.1101/gad.271908.115 PMC4647559

[CR186] Sienski G, Donertas D, Brennecke J (2012) Transcriptional silencing of transposons by piwi and maelstrom and its impact on chromatin state and gene expression. Cell 151:964–980 23159368 10.1016/j.cell.2012.10.040 PMC3504300

[CR187] Sokolova OA, Ilyin AA, Poltavets AS, Nenasheva VV, Mikhaleva EA, Shevelyov YY, Klenov MS (2019) Yb body assembly on the *flamenco* piRNA precursor transcripts reduces genic piRNA production. Mol Biol Cell 30:1544–1554 10.1091/mbc.E17-10-0591 PMC6724695 30943101

[CR188] Son JH, Lawlor MA, Virani M, Cao W, Levine MT, Ellison C (2025) Convergence and conflict among telomere specialized transposons across 60 million years of Drosophilid evolution. Preprint at BioRxiv 10.1101/2025.06.09.658640 10.1101/gr.281112.125 PMC13534243 42386527

[CR189] Song SU, Kurkulos M, Boeke JD, Corces VG (1997) Infection of the germ line by retroviral particles produced in the follicle cells: a possible mechanism for the mobilization of the gypsy retroelement of Drosophila. Development 124:2789–2798 9226450 10.1242/dev.124.14.2789

[CR190] Spradling AC (2024) The ancient origin and function of germline cysts. Results Probl Cell Differ 71:3–21 37996670 10.1007/978-3-031-37936-9_1

[CR191] Spradling AC, Bellen HJ, Hoskins RA (2011) Drosophila P elements preferentially transpose to replication origins. Proc Natl Acad Sci USA 108:15948–15953 21896744 10.1073/pnas.1112960108 PMC3179094

[CR192] Sullivan W, Daily DR, Fogarty P, Yook KJ, Pimpinelli S (1993) Delays in anaphase initiation occur in individual nuclei of the syncytial Drosophila embryo. Mol Biol Cell 4:885–896 8257792 10.1091/mbc.4.9.885 PMC275719

[CR193] Sultana T, Zamborlini A, Cristofari G, Lesage P (2017) Integration site selection by retroviruses and transposable elements in eukaryotes. Nat Rev Genet 18:292–308 28286338 10.1038/nrg.2017.7

[CR194] Sumiyoshi T, Sato K, Yamamoto H, Iwasaki YW, Siomi H, Siomi MC (2016) Loss of l(3)mbt leads to acquisition of the ping-pong cycle in Drosophila ovarian somatic cells. Genes Dev 30:1617–1622 27474440 10.1101/gad.283929.116 PMC4973291

[CR195] Sundaram V, Wysocka J (2020) Transposable elements as a potent source of diverse cis-regulatory sequences in mammalian genomes. Philos Trans R Soc Lond B Biol Sci 375:20190347 32075564 10.1098/rstb.2019.0347 PMC7061989

[CR196] Szakmary A, Reedy M, Qi H, Lin H (2009) The Yb protein defines a novel organelle and regulates male germline stem cell self-renewal in *Drosophila melanogaster*. J Cell Biol 185:613–627 19433453 10.1083/jcb.200903034 PMC2711570

[CR197] Théron E, Maupetit-Mehouas S, Pouchin P, Baudet L, Brasset E, Vaury C (2018) The interplay between the argonaute proteins Piwi and Aub within Drosophila germarium is critical for oogenesis, piRNA biogenesis and TE silencing. Nucleic Acids Res 46:10052–10065 30113668 10.1093/nar/gky695 PMC6212714

[CR198] Vagin VV, Sigova A, Li C, Seitz H, Gvozdev V, Zamore PD (2006) A distinct small RNA pathway silences selfish genetic elements in the germline. Science 313:320–324 16809489 10.1126/science.1129333

[CR199] Vagin VV, Yu Y, Jankowska A, Luo Y, Wasik KA, Malone CD, Harrison E, Rosebrock A, Wakimoto BT, Fagegaltier D et al (2013) Minotaur is critical for primary piRNA biogenesis. RNA 19:1064–1077 23788724 10.1261/rna.039669.113 PMC3708527

[CR200] van Lopik J, Alizada A, Trapotsi M-A, Hannon GJ, Bornelöv S, Czech Nicholson B (2023) Unistrand piRNA clusters are an evolutionarily conserved mechanism to suppress endogenous retroviruses across the Drosophila genus. Nat Commun 14:7337 37957172 10.1038/s41467-023-42787-1 PMC10643416

[CR201] Varjak M, Leggewie M, Schnettler E (2018) The antiviral piRNA response in mosquitoes? J Gen Virol 99:1551–1562 30372405 10.1099/jgv.0.001157

[CR202] Varoqui M, Mohamed M, Mugat B, Gourion D, Lemoine M, Pélisson A, Grimaud C, Chambeyron S (2025) Temporal and spatial niche partitioning in a retrotransposon community of the *Drosophila melanogaster* genome. Nucleic Acids Res 53:gkaf516 40503684 10.1093/nar/gkaf516 PMC12159745

[CR203] Venner S, Feschotte C, Biémont C (2009) Dynamics of transposable elements: towards a community ecology of the genome. Trends Genet TIG 25:317–323 19540613 10.1016/j.tig.2009.05.003 PMC2945704

[CR204] Voichek M, Bernhard A, Novatchkova M, Handler D, Möseneder P, Rafanel B, Duchek P, Senti K-A, Brennecke J (2025) Direct cell-to-cell transmission of retrotransposons. Preprint at BioRxiv 10.1101/2025.03.14.642691 10.1016/j.cell.2026.08.047 42777706

[CR205] Voigt F, Reuter M, Kasaruho A, Schulz EC, Pillai RS, Barabas O (2012) Crystal structure of the primary piRNA biogenesis factor Zucchini reveals similarity to the bacterial PLD endonuclease Nuc. RNA 18:2128–2134 23086923 10.1261/rna.034967.112 PMC3504665

[CR206] Volterra V (1926) Fluctuations in the abundance of a species considered mathematically. Nature 118:558–560

[CR207] Voronina E, Seydoux G, Sassone-Corsi P, Nagamori I (2011) RNA granules in germ cells. Cold Spring Harb Perspect Biol 3:a002774 21768607 10.1101/cshperspect.a002774 PMC3225947

[CR208] Wang C, Lyv L, Solberg T, Zhang H, Wen Z, Gao F (2024) GTSF1 is required for transposon silencing in the unicellular eukaryote *Paramecium tetraurelia*. Nucleic Acids Res 52:13206–13223 39441077 10.1093/nar/gkae925 PMC11602119

[CR209] Wang H, Ma Z, Niu K, Xiao Y, Wu X, Pan C, Zhao Y, Wang K, Zhang Y, Liu N (2016) Antagonistic roles of Nibbler and Hen1 in modulating piRNA 3’ ends in Drosophila. Dev Camb Engl 143:530–539 10.1242/dev.128116 PMC4760310 26718004

[CR210] Wang L, Dou K, Moon S, Tan FJ, Zhang ZZ (2018) Hijacking oogenesis enables massive propagation of LINE and retroviral transposons. Cell 174:1082–1094.e12 30057117 10.1016/j.cell.2018.06.040 PMC6628338

[CR211] Wang L, Tracy L, Su W, Yang F, Feng Y, Silverman N, Zhang ZZZ (2022) Retrotransposon activation during Drosophila metamorphosis conditions adult antiviral responses. Nat Genet 54:1933–1945 10.1038/s41588-022-01214-9 PMC9795486 36396707

[CR212] Wang W, Han BW, Tipping C, Ge DT, Zhang Z, Weng Z, Zamore PD (2015) Slicing and binding by Ago3 or Aub trigger Piwi-bound piRNA production by distinct mechanisms. Mol Cell 59:819–830 26340424 10.1016/j.molcel.2015.08.007 PMC4560842

[CR213] Weick E-M, Miska EA (2014) piRNAs: from biogenesis to function. Development 141:3458–3471 25183868 10.1242/dev.094037

[CR244] Weiss RA (2006) The discovery of endogenous retroviruses. Retrovirology 3:1–11 10.1186/1742-4690-3-67 PMC1617120 17018135

[CR214] Wells JN, Feschotte C (2020) A field guide to eukaryotic transposable elements. Annu Rev Genet 54:539–561 32955944 10.1146/annurev-genet-040620-022145 PMC8293684

[CR215] Wicker T, Sabot F, Hua-Van A, Bennetzen JL, Capy P, Chalhoub B, Flavell A, Leroy P, Morgante M, Panaud O et al (2007) A unified classification system for eukaryotic transposable elements. Nat Rev Genet 8:973–982 17984973 10.1038/nrg2165

[CR216] Wu DC, Johnston LA (2009) Competition among stem cells gets sticky. Cell Stem Cell 5:459–460 19896435 10.1016/j.stem.2009.10.006 PMC2846513

[CR217] Xie W, Gai X, Zhu Y, Zappulla DC, Sternglanz R, Voytas DF (2001) Targeting of the yeast Ty5 retrotransposon to silent chromatin is mediated by interactions between integrase and Sir4p. Mol Cell Biol 21:6606–6614 11533248 10.1128/MCB.21.19.6606-6614.2001 PMC99806

[CR218] Xiol J, Spinelli P, Laussmann MA, Homolka D, Yang Z, Cora E, Couté Y, Conn S, Kadlec J, Sachidanandam R et al (2014) RNA clamping by vasa assembles a piRNA amplifier complex on transposon transcripts. Cell 157:1698–1711 24910301 10.1016/j.cell.2014.05.018

[CR219] Xu J, Zhao X, Mao F, Basrur V, Ueberheide B, Chait BT, Allis CD, Taverna SD, Gao S, Wang W et al (2021) A Polycomb repressive complex is required for RNAi-mediated heterochromatin formation and dynamic distribution of nuclear bodies. Nucleic Acids Res 49:5407–5425 33412588 10.1093/nar/gkaa1262 PMC8191774

[CR220] Yamamoto-Matsuda H, Miyoshi K, Moritoh M, Yoshitane H, Fukada Y, Saito K, Yamanaka S, Siomi MC (2022) Lint-O cooperates with L(3)mbt in target gene suppression to maintain homeostasis in fly ovary and brain. EMBO Rep 23:e53813 35993198 10.15252/embr.202153813 PMC9535798

[CR221] Yamashiro H, Negishi M, Kinoshita T, Ishizu H, Ohtani H, Siomi MC (2019) Armitage determines Piwi−piRISC processing from precursor formation and quality control to inter-organelle translocation. EMBO Rep 21:EMBR201948769 10.15252/embr.201948769 PMC7001504 31833223

[CR222] Yamashiro H, Negishi M, Kinoshita T, Ishizu H, Ohtani H, Siomi MC (2020) Armitage determines Piwi-piRISC processing from precursor formation and quality control to inter-organelle translocation. EMBO Rep 21:e48769 31833223 10.15252/embr.201948769 PMC7001504

[CR223] Yashiro R, Murota Y, Nishida KM, Yamashiro H, Fujii K, Ogai A, Yamanaka S, Negishi L, Siomi H, Siomi MC (2018) Piwi nuclear localization and its regulatory mechanism in Drosophila ovarian somatic cells. Cell Rep 23:3647–3657 29925005 10.1016/j.celrep.2018.05.051

[CR224] Ye J, Pérez-González CE, Eickbush DG, Eickbush TH (2005) Competition between R1 and R2 transposable elements in the 28S rRNA genes of insects. Cytogenet Genome Res 110:299–306 16093682 10.1159/000084962

[CR225] Yoth M, Maupetit-Méhouas S, Akkouche A, Gueguen N, Bertin B, Jensen S, Brasset E (2023) Reactivation of a somatic errantivirus and germline invasion in Drosophila ovaries. Nat Commun 14:6096 37773253 10.1038/s41467-023-41733-5 PMC10541861

[CR226] Yu Y, Gu J, Jin Y, Luo Y, Preall JB, Ma J, Czech B, Hannon GJ (2015) Panoramix enforces piRNA-dependent cotranscriptional silencing. Science 350:339–342 26472911 10.1126/science.aab0700 PMC4722808

[CR227] Zamparini AL, Davis MY, Malone CD, Vieira E, Zavadil J, Sachidanandam R, Hannon GJ, Lehmann R (2011) Vreteno, a gonad-specific protein, is essential for germline development and primary piRNA biogenesis in Drosophila. Development 138:4039–4050 21831924 10.1242/dev.069187 PMC3160098

[CR228] Zhang F, Wang J, Xu J, Zhang Z, Koppetsch BS, Schultz N, Vreven T, Meignin C, Davis I, Zamore PD et al (2012) UAP56 couples piRNA clusters to the perinuclear transposon silencing machinery. Cell 151:871–884 23141543 10.1016/j.cell.2012.09.040 PMC3499805

[CR229] Zhang G, Tu S, Yu T, Zhang X-O, Parhad SS, Weng Z, Theurkauf WE (2018) Co-dependent assembly of Drosophila piRNA precursor complexes and piRNA cluster heterochromatin. Cell Rep 24:3413–3422.e4 30257203 10.1016/j.celrep.2018.08.081 PMC6235161

[CR230] Zhang H-H, Peccoud J, Xu M-R-X, Zhang X-G, Gilbert C (2020) Horizontal transfer and evolution of transposable elements in vertebrates. Nat Commun 11:1–10 32170101 10.1038/s41467-020-15149-4 PMC7070016

[CR231] Zhang Y, Guo R, Cui Y, Zhu Z, Zhang Y, Wu H, Zheng B, Yue Q, Bai S, Zeng W et al (2017) An essential role for PNLDC1 in piRNA 3′ end trimming and male fertility in mice. Cell Res 27:1392–1396 28994417 10.1038/cr.2017.125 PMC5674159

[CR232] Zhang Z, Xu J, Koppetsch BS, Wang J, Tipping C, Ma S, Weng Z, Theurkauf WE, Zamore PD (2011) Heterotypic piRNA Ping-Pong requires qin, a protein with both E3 ligase and Tudor domains. Mol Cell 44:572–584 22099305 10.1016/j.molcel.2011.10.011 PMC3236501

[CR233] Zhao K, Cheng S, Miao N, Xu P, Lu X, Zhang Y, Wang M, Ouyang X, Yuan X, Liu W et al (2019) A Pandas complex adapted for piRNA-guided transcriptional silencing and heterochromatin formation. Nat Cell Biol 21:1261–1272 31570835 10.1038/s41556-019-0396-0

[CR234] Zou S, Voytas DF (1997) Silent chromatin determines target preference of the Saccharomyces retrotransposon Ty5. Proc Natl Acad Sci USA 94:7412–7416 9207105 10.1073/pnas.94.14.7412 PMC23835

