# Supplementary material for: Host-transposable element coexistence: a matter of resistance, tolerance and trade-off
Source: EMBO J. 2026 May 13;45(12):4014–33. doi: 10.1038/s44318-026-00795-z (PMC13269885; doi:10.1038/s44318-026-00795-z)
Supplement: Supplementary file 1 — Peer Review File [file 44318_2026_795_MOESM1_ESM.pdf]

THE EMBO JOURNAL

Host-transposable element coexistence: a matter of resistance, tolerance and trade-off

Severine Chambeyron, Alain Pelisson, Yuica Koga, Charlotte GRIMAUD, and Mikiko Siomi

Corresponding author(s): Mikiko Siomi (siomim@g.ecc.u-tokyo.ac.jp) , Severine Chambeyron (Severine.Chambeyron@igh.cnrs.fr), Charlotte GRIMAUD (charlotte.grimaud@igh.cnrs.fr)

Review Timeline:

Submission Date:	11th Aug 25
Editorial Decision:	22nd Sep 25
Revision Received:	10th Dec 25
Editorial Decision:	23rd Jan 26
Revision Received:	13th Feb 26
Accepted:	10th Apr 26

Editor: Cornelius Schneider

Transaction Report:

(Note: With the exception of the correction of typographical or spelling errors that could be a source of ambiguity, letters and reports are not edited. Depending on transfer agreements, referee reports obtained elsewhere may or may not be included in this compilation. Referee reports are anonymous unless the Referee chooses to sign their reports.)

Dear Dr. Chambeyron,

Thank you for submitting your manuscript for consideration by the EMBO Journal. It has now been seen by three referees whose comments are shown below.

As you can see from the reports all three referees think that the topic is interesting and timely and offer a range of suggestions which I find helpful and productive

Given the referees' positive recommendations, I would like to invite you to submit a revised version of the manuscript, addressing the comments of all three reviewers. I should add that it is EMBO Journal policy to allow only a single round of revision, and acceptance of your manuscript will therefore depend on the completeness of your responses in this revised version.

When preparing your letter of response to the referees' comments, please bear in mind that this will form part of the Review Process File, and will therefore be available online to the community. For more details on our Transparent Editorial Process, please visit our website: https://www.embopress.org/page/journal/14602075/authorguide#transparentprocess

We generally allow three months as standard revision time. As a matter of policy, competing manuscripts published during this period will not negatively impact on our assessment of the conceptual advance presented by your study. However, we request that you contact the editor as soon as possible upon publication of any related work, to discuss how to proceed. Should you foresee a problem in meeting this three-month deadline, please let us know in advance and we may be able to grant an extension.

Thank you for the opportunity to consider your work for publication. I look forward to your revision.

Yours sincerely,

Cornelius Schneider, PhD
Editor
The EMBO Journal
c.schneider@embojournal.org

Instructions for preparing your revised manuscript:

Please make sure you upload a letter of response to the referees' comments together with the revised manuscript.

Please also check that the title and abstract of the manuscript are brief, yet explicit, even to non-specialists.

When assembling figures, please refer to our figure preparation guideline in order to ensure proper formatting and readability in print as well as on screen:
https://bit.ly/EMBOPressFigurePreparationGuideline
See also guidelines for figure legends: https://www.embopress.org/page/journal/14602075/authorguide#figureformat

At EMBO Press we ask authors to provide source data for the main manuscript figures. You will receive a separate email with instructions for providing source data with your revised manuscript, including how to upload and organize the files.

IMPORTANT: When you send the revision we will require
- a point-by-point response to the referees' comments, with a detailed description of the changes made (as a word file).
- a word file of the manuscript text.
- individual production quality figure files (one file per figure)
- a complete author checklist, which you can download from our author guidelines (https://www.embopress.org/page/journal/14602075/authorguide).
- Expanded View files (replacing Supplementary Information)
Please see out instructions to authors
https://www.embopress.org/page/journal/14602075/authorguide#expandedview
- a Reagents and Tools Table as part of the Methods section, which can be downloaded from our author guidelines (https://www.embopress.org/page/journal/14602075/authorguide#structuredmethods)

Please remember: Digital image enhancement is acceptable practice, as long as it accurately represents the original data and conforms to community standards. If a figure has been subjected to significant electronic manipulation, this must be noted in the figure legend or in the 'Materials and Methods' section. The editors reserve the right to request original versions of figures and the original images that were used to assemble the figure.

Further information is available in our Guide For Authors: https://www.embopress.org/page/journal/14602075/authorguide

We realize that it is difficult to revise to a specific deadline. In the interest of protecting the conceptual advance provided by the work, we recommend a revision within 3 months (21st Dec 2025). Please discuss the revision progress ahead of this time with the editor if you require more time to complete the revisions. Use the link below to submit your revision:

Referee #1:

Review of "News from the Battlefield: Adaptive strategies ensuring peaceful coexistence between transposable elements and their hosts"
This review addresses a relevant and timely topic-the interplay between transposable elements (TEs) and host defense pathways, with an emphasis on the animal piRNA pathway. Given the rapid pace of discovery in the field of TE-host co-evolution, a clear and well-structured synthesis would be valuable for the community. Unfortunately, I feel that in its current form, the manuscript does not yet achieve this goal. Below I outline my main concerns and suggestions for improvement.

1. Scope and Framing
The review promises to cover adaptive strategies that ensure coexistence between TEs and their hosts. However, a considerable portion of the manuscript is focused on aspects of the piRNA pathway that seem only loosely connected to the overall theme of the review. Often, Drosophila-specific details are being discussed without sufficient contextualization. At the same time, important domains on the host side such as KRAB-ZFP biology, ciliate small RNAs, or recombination-based TE removal are treated superficially. This imbalance makes it difficult for the reader to grasp a unifying conceptual framework.

I recommend that the authors carefully reframe the first section of the review (host side) to establish and work out overarching themes. Such conceptual frameworks would help readers navigate the complexity rather than feel overwhelmed by it.

2. Clarity and Synthesis
Several sections read as a list of findings rather than as a synthesis that draws connections or identifies common principles, whch would be the ideal goal of a high-rofile review article. For example:

• piRNA cluster transcription (1.1.2.): The section begins abruptly with a Drosophila-specific case (dual-strand piRNA clusters). A broader introduction and a more integrative perspective are needed. What is the challenge on the host side? How can adaptive processes in different species with different piRNA cluster biology be understood or conceptualized?
• Protein-focused subsections (Yb, Gtsf1, Mael, Vreteno, Zucchini, etc.): These sections lack a comparative discussion and fail to connect individual findings into a coherent picture with conceptual value. They also seem rather diconnected from the overall topic pf the review and it is unclear what they add in terms of value. Some of the mentioned aspects (e.g., phase separation in Yb bodies, evolutionary scenarios for Zucchini, Vreteno/TDRD1 divergence) need a more balanced, critical treatment. Why could an endonuclease not have been the original enzyme rather than a phospho-lipase? Comparing Vreteno alone against TDRD1 is challenging in my opinion given that there seem to be three TDRD1 homologs in Drosophila melanogaster.
• Sections 1.1.2. and 1.1.5 are unnecessarily split as they thematically overlap.
• Sections 1.1.6. (ciliate biology) and 1.2. (KRAB-ZFPs) are too brief in their current form to be useful beyond listing some recent findings; if the authors want to add tehse sections, a conceptual linking to the piRNA pathway by discussing the differences/commonalities/ evolutionary dynmaics would be beneficial.
• At the end of the piRNA-specific chapters, the authors state: "In summary, the piRNA pathway exemplifies both evolutionary conservation and divergence: while its core architecture is preserved across species, specialized adaptations have evolved to meet unique environmental and genomic challenges." [While this is certainly an interesting point, one does wonder whether this is not also true for many other cellular pathways. Is the piRNA pathway special?]
• Throughout, the manuscript would benefit from stepping back to emphasize the bigger picture: e.g. what are the new principles have we learned from the diverse phost defense pathways? How do they relate to the challenges posed by the diverse set of TEs and their molecular and adaptive strategies?

3. Missing References and Imbalance in Coverage
• Some important citations are missing (e.g., PMID: 40209716 for tj in flamenco transcription; PMID: 39054057 on DNA damage upon P-element activation).
• The discussion of mammalian KRAB-ZFPs and their massive lineage-specific expansion is underdeveloped relative to its importance. Moreover, KRAB-ZFPs have radiated in tetrapods and not only in mammals.

• Conversely, subtle mechanistic details from Drosophila piRNA biology are treated at disproportionate length. A more balanced coverage would align better with the stated theme of host-TE coexistence.

4. Conceptual Connections
At several points, the manuscript introduces interesting but loosely connected topics (e.g., germline cell elimination, rDNA as "safe havens," insertion timing). I find the evolutionary arguments underlying these sections rather challenging, especially when speaking about adaptive processes, a very challenging topic in evolutionary theory. I suggest the authors to frame bigger-level questions and then use the individual examples as possible scenarios with a more careful discussion of their evolutionary dynamics. Can the rDNA unit really be considered a safe haven? Is germline cell eliminaion in response to TE activity an adaptive process?

Other comments:
• The claim that the piRNA pathway exemplifies adaptation to "environmental challenges" is not convincingly substantiated. What are concrete examples of such adaptations?
• Horizontal transfer of TEs (2.3.2) is an important topic that deserves a more prominent and integrated discussion rather than a passing mention as horozontal TE transfer likely plays a major role in the diversification of TEs.

5. Style and Accessibility
The review currently assumes a high level of prior knowledge in the individual sections. For readers outside the immediate field, the lack of introductory framing, comparative summaries, and integrative schematics will make it difficult to follow.

6. Other Points
A). Grammar, phrasing, and accuracy of the written word need attention in several places. Acronyms such as URI (section 2.2.2) should be defined upon first use.
Some other concrete examples:
• Title: "News from the Battlefield: Adaptive strategies ensuring peaceful coexistence between transposable elements and their hosts".I suggest that in times of several active wars, the rhetoric might require a second thought.

• "Transposable elements (TEs) are ubiquitous, repetitive and foreign DNA sequences." Tranpsosons per se are rarely repetitive. It is their multiple insertions in host genomes that underlies this statement. I am also not sure whether they should be called 'foreign'. In the end, they sit in our DNA.

• "To survive mutational decay". I am not sure whether the personification of TEs (survive) is adequate.

• "must insert new copies of themselves into the host germline, a process that can compromise host fertility by disrupting coding regions, regulatory sequences and/or remodeling chromosomal architecture."
the new copies need to be in the germline genome, not the germline.

• "In response, hosts deploy multiple layers of genetic and epigenetic regulation to suppress TE activity and reduce their fitness." Is the phrase of genetic and epigenetic ideal in this context? What is genetic regulation of TEs? And how about epigenetic regulation? I think these terms are not helpful to understand what is really happening.

• "Like the original donor copies, these new insertions can replicate and are therefore subsequently inherited by the progeny." Logic? The new copies are immediately inherited to the progeny, not only upon their further replication.

• "In other cases, the host's biology has become more or less dependent on ongoing TE activity, despite the damages potentially caused by new insertions." [needs references]

• "in which piRNAs originating mostly from specialized genomic piRNA clusters, guide PIWI proteins to recognize and silence TEs in the germline". [piRNA biology is not restricted to germline cells in many organisms].

• Both small RNAs trigger RNA silencing, i.e., downregulation of target gene expression through RNA-RNA base-pairing with complementary transcripts, by associating with members of the Argonaute family in RNA-Induced Silencing Complexes called piRISC and siRISC, respectively (Ghildiyal and Zamore, 2009; Liu and Paroo, 2010). However, they have many distinct features. [grammar; very long sentence].

B). Other comments.

• Section 1.1.1.
It is unclear to me what the over-arching goal here is? To give an update on recent piRNA biology? If yes, then the connection to the title seems rather thin and the examples are very much Drosophila melanogaster centric. If the goal is to use the flexibility in the piRNA hardware as an argument for host adaptation or similar theories, then a better framing of the entire section would be important.

For the Yb section: I recommend starting with some general established facts on the function of Yb before going into the more controversial/speculative thesis of phase-separated condensates and their role in piRNA biogenesis.
• Section 1.1.2. piRNA cluster transcription.
Before attempting any broader introduction into the topic, the first paragraph jumps into a highly specialized case in Drosophila without setting the frame. For the outsider this is not helpful and not understandable. This entire section lacks an attempt to synthesize a bigger picture. The extreme focus on Drosophila seems counter to the theme of the review. Also, section 1.1.5 seems to thematically fit much better together with section 1.1.2.
• Section 1.1.3.
This entire section reads a bit like a laundry list of findings from the literature without the attempt to synthesize these findings into a bigger picture or to bring disparate observations together into a clearer picture (expected from a review).
For both, Gtsf1 and Mael, the authors could consider updating their discussion about a common molecular function by incorporating recent findings that came out while this review was written (PMID: 40912244 and PMID: 40912245).
• Section 1.2.2.
This chapter seems detached from the rest as it is not discussed whether recombination-mediated loss of TE sequences (e.g. by LTR-LTR recombination) is an active, promoted process or a passive process.
• Section 1.3.
While an interesting overall topic, connecting evolutionary arguments to a limited level of understanding how the environment impacts TE biology and host defense biology, this chapter risks at being too simplistic in my opinion.
• Section 2.1.2.
This is an overall interesting chapter that might further benefit from a more structured intro where it could be pointed out that most TEs must be expressed in germline cells as they cannot cross cell boundaries. And then explain two known scenarios how this paradigm can be altered. First, by evolving infectivity modules, second by exploiting the syncytial nature of germline development known from many organisms.
• Section 2.1.3.
This is another chapter where I find it exceedingly challenging to make strong conclusions. Whether the rDNA units can be considered safe havens is a very difficult topic in my opinion. And to then combine this topic of safe heavens with insertion site preferences for the P-element is also a bit of a stretch. Surely, such promoter regions cannot be considered as safe havens, which is the title of the section.
• Section 2.1.4.
This section reads rather simplistic to me. Considering timing of integration, certain hard-wired features are important to keep in mind. For example, DNA transposons can only amplify in replicating cells due to their cut & paste mechanism. LTR retrotransposons on the other hand can be expressed at later stages of gametogenesis that lack mitotic activity. I suggest framing the arguments under the overall umbrella of molecular realities that different TEs or the host faces.

Conclusion
In summary, while the manuscript covers an important and timely topic, it would benefit from a substantial revision before it can serve as a useful resource for the community. The authors should reframe the review around unifying conceptual themes, provide more balanced coverage across pathways and organisms, and synthesize findings into a coherent narrative. With these changes, the article has the potential to make a valuable contribution.

Referee #2:

This review provides a clear and well-structured synthesis of knowledge on dynamic interactions among transposable elements (TEs) and host genomes. Describing as an extended evolutionary "arms race" tempered by mutual adaptation, the authors highlight both the antagonistic and cooperative nature of the relationship. In particular, the authors have emphasized the roles of piRNA pathways as primary defenders of germline genome integrity, and discussed how TE silencing operates on both transcriptional and post-transcriptional levels. The authors have also argued that host-TE interactions barely ever lead to the definitive extinction of either partner; instead, they settle in dynamic equilibria in which TEs are kept in niches that inflict least damage and hosts build layers of protection. A particular strength of this review article is the use of ecological metaphors, in considering the genome as an "ecosystem" in which different TE families subdivide space and resources. This perspective, in addition to placing diverse observation into a coherent scheme, also opens new avenues toward quantitative and systems-wide work. In sum, the review is timely, well-focused, and amply supported by a broad range of literature. The subsequent comments are intended to offer suggestions for further improvement.
Major points:
1） Since the authors state in section 1.1 and Figure 1 that TE repression is mediated by host small RNAs, including both piRNAs and endo-siRNAs, the manuscript would benefit from a brief discussion of endo-siRNAs alongside piRNAs. While it is reasonable to emphasize piRNAs under the theme of TE regulation, a minimal acknowledgment of endo-siRNAs would provide a more balanced perspective.
2） As this manuscript mainly focuses on the adaptive strategies ensuring peaceful coexistence between transposable elements and their hosts from the perspective of ecological frameworks adopted from community ecology, it would be beneficial to elaborate more explicitly on these concepts in the relevant sections.

3） For the clarity to a broader readership, it would be helpful if the conserved piRNA pathway factors listed in Table 2 were annotated with their mammalian homologs. This would facilitate understanding for researchers from fields beyond insect biology.
4） In section 1.1.2, given that dual-strand clusters lack canonical promoter regions, the authors should better discuss the molecular signals that recruit the RDC complex to these loci.
5） In section 1.1.2, the discussion is framed as addressing species divergence in piRNA cluster transcription; however, the description focuses exclusively on uni-strand and dual-strand clusters in Drosophila. This narrow focus does not fully reflect the stated theme of species divergence and could be broadened with examples from other taxa.
6） Figure 4 provides a schematic overview of strategies employed by TEs for long-term propagation. To enhance the impact and scientific rigor, it would be beneficial to add some examples from published studies in the Figure.
Minor points:
1） The factor Armi should be included in Table 2.
2） In section 1.2.1, the statement "High expression of KRAB-ZFPs is frequently observed in early embryonic cells and the germline, developmental windows in which epigenetic reprogramming renders the genome particularly vulnerable to TE activation" requires appropriate references.
3） In section 2.1.1, the nomenclature of "MERVL" and "HERV-K" should be standardized for consistency.
4） In section 2.2.3, the claim that "Since HERV-K activation is OCT4-dependent and occurs under genome-wide hypomethylation, it has been proposed that primordial germ cells (PGCs) may similarly harness HERV-K to fend off viral threats." requires specific supporting citations.
5） In section 2.2.3, the second paragraph could be merged with the first, as both address the influence of transposable elements on innate immune responses.

Referee #3:

In this review the authors discuss host-TE co-evolution. This is a huge topic but the authors do quite a good job of organising the large amount of information into two major aspects: evolution of host factors that control TEs and evolution of the TEs themselves such that they evade host defences.
I enjoyed reading this review but have a few important comments about the content, mostly regarding the first half of the review on the evolution of TE-controlling factors. Crucially, the topic of the review is TE/genome coexistence. Much of the discussion in the first half is about the evolution of piRNAs, focussing on some specific proteins. It is not made explicit in the article how this actually relates to the topic. Whilst of course it is plausible that the reason that piRNAs evolve fast is the TE/host arrms race, this is still a conjecture. Plenty of other molecular pathways evolve fast; moreover the 'fast evolution' idea is quite difficult to actually quantify; how fast would you expect a pathway to evolve? Do piRNA factors evolve faster than the norm (probably, but this hasn't really been directly demonstrated very often)? Even if they do, is this because of their function or simply because of the type of proteins that regulate the piRNA pathway (which might simply be robust to mutations so can accumulate diversity rapidly)? And finally, it's important to note that piRNAs in many arthopods have an antiviral role, rather than just TEs, which might also lead to fast evolution. These might seem far-fetched but they are important caveats to the argument. It's important that the authors discuss this before listing their examples. Importantly none of the examples actually directly link to TEs, so it's vital that the link is made clear so that the information is relevant to the overall questions. Below, there are some more specific comments about these sections.

Another crucial point that would be worth adding to this review is the interesting point that piRNAs can act as a 'double-edged sword' when it comes to TE control. piRNAs suppress TEs, but paradoxically this enables the genome to tolerate more TE copies. It's possible that loss of the piRNA pathway might actually be ultimately beneficial in meaning that purifying selection eliminates TEs (thus enabling ultimately a higher fitness to be reached, albeit traversing through a region of the fitness landscape with reduced fitness due to the loss of the piRNA-mediated repression). This model was first described computationally by Lu and Clark (https://genome.cshlp.org/content/20/2/212). There is also some empirical evidence for it (10.15252/msb.20209600).

Specific comments
Section 1
The examples here are drawn heavily from Drosophila, with some information from mammals; it is fine but it's worth specifying upfront that there are large groups of organisms that are not considered- e.g. other arthropods (see also another comment below), nematodes, for which quite a lot of mechanistic information about the piRNA pathway and its relationship to TEs is known.
1.1.1 Only 2 egs are chosen here and this isn't really explained properly- could the specific relevance of these be expanded on slightly? Is it just because they are very recent? Perhaps it's better if this is rewritten to point out some general aspects of piRNA pathway divergence, using these two as specific examples, rather than just picking them, which implies that each protein evolves idiosyncratically and the general relevance to TE silencing evolution is not then clear.
1.1.2 Again this needs to be more clearly linked to TEs, which aren't mentioned at all in this section
1.1.3 This is not really relevant to TE silencing, unless the point is that the fast evolution is somehow facilitated by the fact that piRNA pathways can incorporate other proteins easily thus enabling them to evolve fast; if this is the argument then it should be stated explicitly.

1.1.4 The link to TEs is again absent here.
1.1.5 This doesn't seem relevant to the review but I think that it could be made much more on-topic by including a discussion of a key issue in piRNA evolution which is not touched on: why it has become germline-restricted so often in evolution. Taking the examples of arthropods, the ancestral arthropod piRNA pathway was almost certainly somatic as well as germline. However, Drosophila evolved germline-restricted piRNAs, which of course links directly to the transcriptional control (gain of sites for a germline-restricted TF for piRNA factors). It's very likely that the somatic gonad piRNA pathway was then a secondary innovation after germline restriction evolved, to resist a new TE family. This is a really good example of TE/host arms race, but it still begs the question of why piRNAs ever become germline restricted. Perhaps there is a cost associated with TE silencing that is not 'worth it' in the soma (maybe targeting other genes by mistake)? In any case, this is an important discussion to have at this point in the review.
1.1.6. This is interesting, but seems out of place given the preceding sections which are about evolution of piRNA pathways, whereas this is a distinct piRNA function. Could the authors do a bit better at linking this in with the previous sections, perhaps by emphasizing that it is an example of how the entire mechanism of the piRNA pathway can evolve in completely distinct directions in response to the unusual biology of the ciliates.
1.2.2 This section is quite misleading at the moment. All the other points discussed are 'active' functions of control mechanisms, but here the argument is about mutations that presumably occur randomly but can lead to loss of a TE, which may then be acted on by selection and go to fixation. This isn't really on topic and should be removed, especially as the way it's currently written implies that there's some kind of active mutagenesis going on here akin to the ciliate programmed genome elimination, which as far as we know is not the case here.
1.3 This would be the place to incorporate the 'double edged sword' idea, maybe touching on the fact that some species have indeed lost piRNAs altogether (many nematodes, parasitic flatworms, dust mites).
2.2 I may have missed it but I didn't see anything here about the famous Rag tranposase in vertebrate immune system, which, although hackneyed, is worth including here (I think many readers will be expecting it).

Referee #1

1. Scope and Framing

The review promises to cover adaptive strategies that ensure coexistence between TEs and their hosts. However, a considerable portion of the manuscript is focused on aspects of the piRNA pathway that seem only loosely connected to the overall theme of the review. Often, Drosophila-specific details are being discussed without sufficient contextualization. At the same time, important domains on the host side such as KRAB-ZFP biology, ciliate small RNAs, or recombination-based TE removal are treated superficially. This imbalance makes it difficult for the reader to grasp a unifying conceptual framework. I recommend that the authors carefully reframe the first section of the review (host side) to establish and work out overarching themes. Such conceptual frameworks would help readers navigate the complexity rather than feel overwhelmed by it.

We appreciate the reviewer's thoughtful and constructive comments. In response to feedback from this reviewer and the other two reviewers, we have substantially restructured Section 1, now entitled "*Host-centered strategies for host–TE coexistence*."

In the revised version, Section 1 (page 4 to 14) begins by outlining the basic framework of the piRNA pathway. We then provide an overview of the *Drosophila* piRNA pathway, followed by a discussion of cell type-specific diversification of piRNAs in two ovarian cell types, OSCs and OGCs, addressing both their shared framework and distinct features. Although these two cell types coexist within the same ovarian tissue, their piRNA mechanisms differ, and we now describe this contrast explicitly as a unifying example of host adaptation to distinct TE challenges.

Our rationale is as follows: TEs integrate into host genomes, and their transposition can damage host genes. Consequently, hosts repress TEs via the piRNA pathway. Paradoxically, hosts that fail to restrain TEs lose reproductive capacity owing to excessive transposition, thereby eliminating the genomic niche that sustains TEs. Thus, the piRNA pathway functions not only as a defense system against TEs but also as an essential mechanism enabling stable host–TE coexistence. We therefore consider it reasonable that the host-focused section begins with piRNA-mediated TE silencing as its central framework.

In the course of this restructuring, we have not responded to each of the reviewer's questions individually (for which we sincerely apologize), but we hope that this reviewer, as well as the other two reviewers, will find the revised structure of Section 1 more clearly framed around overarching themes and better aligned with the main topic of host–TE coexistence.

2. Clarity and Synthesis

Several sections read as a list of findings rather than as a synthesis that draws connections or identifies common principles, which would be the ideal goal of a high-profile review article. For example:

• piRNA cluster transcription (1.1.2.): The section begins abruptly with a Drosophila-specific case (dual-strand piRNA clusters). A broader introduction and a more integrative perspective are needed. What is the challenge on the host side? How can adaptive processes in different species with different piRNA cluster biology be understood or conceptualized?

We appreciate the reviewer's insightful comment. In revised Section 1, we have added new subsections, "*piRNA-mediated TE repression: basic framework*" (page 4) and "*The piRNA mechanism in the Drosophila ovary: overview*" (page 5), as broader introductions for readers.

This now precedes "*Cell type-specific diversification within one host: OSC vs OGC*." Here, we first outline the fundamental framework of the piRNA mechanism in OSCs and OGCs and then summarize how these adjacent cell types deploy distinct strategies against TEs within the same ovarian tissue. We also explain that these cell-specific strategies arise from differences in TE behavior and routes of invasion in each cell type, thereby making the host-side challenges more explicit and offering a more integrative perspective on piRNA cluster transcription.

As noted above, we have substantially restructured Section 1. In the course of this restructuring, we have not responded to each of the reviewer's questions individually (for which we sincerely apologize), but we hope that this reviewer, as well as the other two reviewers, will find the revised Section 1 more clearly framed around overarching themes and better aligned with the main topic of host–TE coexistence.

• Protein-focused subsections (Yb, Gtsf1, Mael, Vreteno, Zucchini, etc.): These sections lack a comparative discussion and fail to connect individual findings into a coherent picture with conceptual value. They also seem rather diconnected from the overall topic pf the review and it is unclear what they add in terms of value. Some of the mentioned aspects (e.g., phase separation in Yb bodies, evolutionary scenarios for Zucchini, Vreteno/TDRD1 divergence) need a more balanced, critical treatment. Why could an endonuclease not have been the original enzyme rather than a phospho-lipase? Comparing Vreteno alone against TDRD1 is challenging in my opinion given that there seem to be three TDRD1 homologs in Drosophila melanogaster.

We appreciate the reviewer's thoughtful comment. As noted above, we have substantially reworked Section 1 based on feedback from this reviewer and the other two reviewers. In line with this and in consideration of the overall length, we have removed the protein-focused subsections (Yb, Gtsf1, Mael, Vreteno, Zucchini, etc). We hope the reviewer agrees that these changes are appropriate and satisfactorily address the concerns raised.

• *Sections 1.1.2. and 1.1.5 are unnecessarily split as they thematically overlap.*

Section 1 has been substantially restructured, leading to major revisions of the materials formerly in 1.1.2 and 1.1.5. We hope the reviewer agrees that these changes are appropriate and satisfactorily address the concerns raised.

• *Sections 1.1.6. (ciliate biology) and 1.2. (KRAB-ZFPs) are too brief in their current form to be useful beyond listing some recent findings; if the authors want to add tehse sections, a conceptual linking to the piRNA pathway by discussing the differences/commonalities/ evolutionary dynmaics would be beneficial.*

We agree that the original Sections 1.1.6 (ciliate biology) and 1.2 (KRAB-ZFPs) were too concise. In the revised manuscript, we now mention KRAB-ZFPs only briefly in the Introduction (page 2), highlighting their presence and explaining how they differ from the piRNA pathway. The discussion of ciliate biology has been expanded and is now entitled "*1.2) Programmed DNA elimination as an extreme mode of host–TE coexistence*" (page 11). We hope that these changes address the reviewer's concerns and improve the overall coherence of the review.

• *At the end of the piRNA-specific chapters, the authors state: "In summary, the piRNA pathway exemplifies both evolutionary conservation and divergence: while its core architecture is preserved across species, specialized adaptations have evolved to meet unique environmental and genomic challenges." [While this is certainly an interesting point, one does wonder whether this is not also true for many other cellular pathways. Is the piRNA pathway special?]*

We appreciate the reviewer's comment. In light of this concern, we have removed the concluding sentence of the piRNA section. We agree that the broader issue—that the piRNA pathway combines deep evolutionary conservation with pronounced lineage-specific diversification—merits a more thorough, dedicated discussion. To maintain a clear scope in the present review, we do not develop this point further here, but we plan to address it in a future manuscript.

• Throughout, the manuscript would benefit from stepping back to emphasize the bigger picture: e.g. what are the new principles have we learned from the diverse phost defense pathways? How do they relate to the challenges posed by the diverse set of TEs and their molecular and adaptive strategies?

We appreciate this reviewer's comment. We have substantially revised Section 1 based on comments from this reviewer and the other two reviewers.

We now begin by outlining the basic framework of the piRNA pathway, followed by a description of the piRNA mechanism in *Drosophila*. We then discuss the physiological significance and mechanisms of the distinct piRNA systems in *Drosophila* OSCs and OGCs in TE repression, explicitly highlighting the differences between them.

The divergence between OSCs and OGCs reflects the distinct modes of TE repression required in each cell type. OSCs must intercept exogenous, horizontally entering TEs (errantiviruses) before particle formation and germline invasion; accordingly, they deploy the *Flam*–Yb–Piwi axis to enforce rapid transcriptional shutdown. Because rare escapees can traverse from OSCs to OGCs, the OGC system must also contend with such inputs; more broadly, however, OGCs face reactivation of endogenous genomic copies during development and therefore employ a two-tier strategy—Piwi-dependent TGS plus Aub-dependent PTGS—well suited to repressing dispersed TE copies across the genome. By framing these contrasting strategies as an example of how host defense pathways adapt to distinct TE challenges, we aim to step back to a clearer "big picture." We believe these revisions address the reviewer's concerns.

3. Missing References and Imbalance in Coverage

• Some important citations are missing (e.g., PMID: 40209716 for tj in flamenco transcription; PMID: 39054057 on DNA damage upon P element activation).

We cited both references in the revision (page 8 for *PMID: 40209716 and pages 13 and 18 for PMID: 39054057*).

• The discussion of mammalian KRAB-ZFPs and their massive lineage-specific expansion is underdeveloped relative to its importance. Moreover, KRAB-ZFPs have radiated in tetrapods and not only in mammals.

We appreciate the reviewer's comment. In the revised manuscript, we have chosen to keep the main focus on piRNA-based mechanisms (as host-centered TE defense mechanism) and therefore confine our discussion of KRAB-ZFPs to a brief note in the Introduction (page 2). We

now highlight their lineage-specific expansion in tetrapods, their role in TE repression, and how this pathway conceptually differs from the piRNA system. We feel that this level of treatment is more consistent with the scope and length constraints of the present review while still acknowledging the importance of KRAB-ZFP-mediated TE control.

• *Conversely, subtle mechanistic details from Drosophila piRNA biology are treated at disproportionate length. A more balanced coverage would align better with the stated theme of host-TE coexistence.*

We appreciate the reviewer's comment. In the revised manuscript, we have reduced the amount of subtle mechanistic detail in *Drosophila* piRNA biology. We hope that this, together with other revisions made, helps to better balance the coverage and address the reviewer's concern.

4. Conceptual Connections

At several points, the manuscript introduces interesting but loosely connected topics (e.g., germline cell elimination, rDNA as "safe havens," insertion timing). I find the evolutionary arguments underlying these sections rather challenging, especially when speaking about adaptive processes, a very challenging topic in evolutionary theory. I suggest the authors to frame bigger-level questions and then use the individual examples as possible scenarios with a more careful discussion of their evolutionary dynamics.

We agree that our initial questions were too challenging in terms of evolutionary theory. Accordingly, instead of paying much attention to the selective forces that putatively drove mutual adaptations, we have now reframed our review to a series of host and TE features that might have contributed to present-day TE-host coexistence. As suggested by the referee, we introduced the topics of specific insertions niches as follows: "*Many TEs display distinct insertion site preferences within host genomes. These preferences illustrate that certain genomic regions can tolerate transposition without causing major deleterious effects on host fitness. Such regions may represent insertion niches where the negative consequences of integration are effectively buffered (Sultana et al., 2017). However, to which extent these sites qualify as true "safe havens" remains debated. Below, we provide examples illustrating how different TEs accommodate their integration to specific niches within their host genomes*." (see page 17).

Can the rDNA unit really be considered a safe haven?

To explain more precisely why rDNA locus could be considered as a "safe haven" as already suggested by others (Sultana et al., 2017), we modified this paragraph as follows:

"*In a wide range of organisms, the R2 LINE specifically targets the ribosomal DNA (rDNA) repeats (Kojima et al., 2016). To avoid collision between transcription and replication machineries, cells have adopted a compartmentalization of the multiple rDNA copies into those that are actively transcribed and those that initiate replication. They therefore must carry more rDNA copy number than minimally required for sufficient rRNA cell production (Nelson 2023). This could also explain why disruption of a few rDNA repeats by TEs does not impair the ribosomal function, thereby minimizing harmful effects on host fitness*." (see page 17).

Is germline cell elimination in response to TE activity an adaptive process?

We agree with the reviewer that germline cell elimination is a natural process that gets rid of all types of defective cells, irrespectively of the threat. Although TE activity by inducing DSB can trigger this elimination, this may not be considered as an adaptive process developed by the host against TEs. Accordingly, we removed this section.

Other comments:

• The claim that the piRNA pathway exemplifies adaptation to "environmental challenges" is not convincingly substantiated. What are concrete examples of such adaptations?

We appreciate this reviewer's helpful comment. We agree that, in the original manuscript, examples of how the piRNA pathway adapts to the environmental challenges caused by repeated TE invasions were not highlighted clearly enough. In the revised version, we now emphasize the divergent strategies employed by the piRNA pathway in two adjacent ovarian cell lineages, OSCs and OGCs, as an example of adaptation to distinct modes of TE invasion. In addition, we have incorporated a discussion of adaptation via cluster capture and *de novo* piRNA production. We hope that these examples more convincingly substantiate our claims and address the reviewer's concern.

• Horizontal transfer of TEs (2.3.2) is an important topic that deserves a more prominent and integrated discussion rather than a passing mention as horizontal TE transfer likely plays a major role in the diversification of TEs.

As suggested by the reviewer, we have added a paragraph in the Introduction of Section 2.3 (page 22) to highlight the importance of horizontal transfers in TE diversification.

5. Style and Accessibility

The review currently assumes a high level of prior knowledge in the individual sections. For readers outside the immediate field, the lack of introductory framing, comparative summaries, and integrative schematics will make it difficult to follow.

We appreciate the reviewer's insightful comment. In the revised manuscript, we have prioritized improving accessibility for readers outside the immediate field by (i) providing a clearer introductory framework, (ii) adding concise comparative summaries where appropriate, and (iii) including an integrated schematic figure that visually organizes the key concepts. We believe these changes enhance readability and address the reviewer's concerns.

6. Other Points

A). Grammar, phrasing, and accuracy of the written word need attention in several places. Acronyms such as URI (section 2.2.2) should be defined upon first use.

This acronym has been removed and replaced by the complete name of the protein.

Some other concrete examples:

• Title: "News from the Battlefield: Adaptive strategies ensuring peaceful coexistence between transposable elements and their hosts". I suggest that in times of several active wars, the rhetoric might require a second thought.

We agree with the reviewer and decided to change the tittle as follows: "Host–transposable element coexistence: a matter of resistance, tolerance, and trade-off."

• "Transposable elements (TEs) are ubiquitous, repetitive and foreign DNA sequences." Tranpsosons per se are rarely repetitive. It is their multiple insertions in host genomes that underlies this statement.

I am also not sure whether they should be called 'foreign'. In the end, they sit in our DNA.

"To survive mutational decay". I am not sure whether the personification of TEs (survive) is adequate.

• "must insert new copies of themselves into the host germline, a process that can compromise host fertility by disrupting coding regions, regulatory sequences and/or remodeling chromosomal architecture." the new copies need to be in the germline genome, not the germline.

We have addressed all comments regarding our Abstract. We removed the word "foreign" and replaced “repetitive” with “as multiple copies.” Instead of using "survive," we opted for “persist,” and we also revised the wording of the final point accordingly.

The revised abstract is:" *Transposable elements (TEs) are ubiquitous, mobile DNA elements that often exist as multiple copies within host genomes. To persist despite ongoing mutational decay, these genomic parasites must continuously generate new insertions into the germline genome, a process that risks compromising host reproductive capacity by disrupting coding regions and regulatory sequences and by reshaping chromosomal architecture. Accordingly, hosts have evolved mechanisms to repress TE activity and reduce its fitness costs. In principle, however, such antagonism could lead to lineage-specific TE extinction, which may be suboptimal for hosts, as TEs possess aspects that confer beneficial functions to them. Therefore, hosts not only resist but also tolerate and even exploit the presence of TEs. In parallel, TEs themselves have acquired and deployed unique strategies that promote their own persistence while limiting harm to the host. This review explores how “peaceful” coexistence between hosts and TEs is achieved, focusing on resistance, tolerance, and the associated trade-offs from both host and TE perspectives*.” (page 1).

• *"In response, hosts deploy multiple layers of genetic and epigenetic regulation to suppress TE activity and reduce their fitness." Is the phrase of genetic and epigenetic ideal in this context? What is genetic regulation of TEs? And how about epigenetic regulation? I think these terms are not helpful to understand what is really happening.*

We have modified this sentence in the Abstract. “*Accordingly, hosts have evolved mechanisms to repress TE activity and reduce its fitness costs”.* (page 1).

• *"Like the original donor copies, these new insertions can replicate and are therefore subsequently inherited by the progeny." Logic? The new copies are immediately inherited to the progeny, not only upon their further replication.*

For clarity, we have removed this sentence and modified the Introduction section as follows: “*Like their hosts, TEs encode genes whose ultimate goal is to ensure their own survival and propagation across generations. To this end, new TE copies become inserted elsewhere in the host genome, which frequently generates a wide spectrum of host-genome mutations, including insertions, duplications, translocations, deletions, and inversions, with the potential to disrupt gene expression and function (Payer and Burns, 2019) and even to remodel chromosomal architecture (Choudhary et al., 2023; Haws et al., 2022; Li and Shen, 2023)”.* (see page 2).

• "In other cases, the host's biology has become more or less dependent on ongoing TE activity, despite the damages potentially caused by new insertions." [needs references]

We have added a reference (Chang et al., 2025) to illustrate our sentence (page 3).

• "in which piRNAs originating mostly from specialized genomic piRNA clusters, guide PIWI proteins to recognize and silence TEs in the germline". [piRNA biology is not restricted to germline cells in many organisms].

To illustrate that piRNA biology extends beyond the germline in many organisms, we added a sentence in the section *piRNA-mediated TE repression: basic framework*: "*Moreover, in some organisms, such as mosquitoes, piRNAs are not restricted to the germline and have well-established antiviral roles* (Haase et al., 2024; Miesen et al., 2016; Varjak et al., 2018)." (pages 4-5).

• Both small RNAs trigger RNA silencing, i.e., downregulation of target gene expression through RNA-RNA base-pairing with complementary transcripts, by associating with members of the Argonaute family in RNA-Induced Silencing Complexes called piRISC and siRISC, respectively (Ghildiyal and Zamore, 2009; Liu and Paroo, 2010). However, they have many distinct features. [grammar; very long sentence]

Regarding endo-siRNAs, we now only briefly mentioned their existence in the revised Introduction (page 2), along with a brief comparison of their similarities and differences with piRNAs. We hope that these revisions address the reviewer's concerns. We have made a concerted effort to shorten sentences and improve readability throughout.

B). Other comments.

• Section 1.1.1.

It is unclear to me what the over-arching goal here is? To give an update on recent piRNA biology? If yes, then the connection to the title seems rather thin and the examples are very much Drosophila melanogaster centric. If the goal is to use the flexibility in the piRNA hardware as an argument for host adaptation or similar theories, then a better framing of the entire section would be important.

We appreciate the reviewer's thoughtful comments. As noted above, in response to feedback from this reviewer and the other two reviewers, we have substantially restructured Section 1. The revised version is now more clearly organized around overarching themes and better

aligned with the main topic of host–TE coexistence. We hope that this reviewer, as well as the other two reviewers, will find the new structure clearer and more closely connected to the central theme of the review.

For the Yb section: I recommend starting with some general established facts on the function of Yb before going into the more controversial/speculative thesis of phase-separated condensates and their role in piRNA biogenesis.

We appreciate the reviewer's thoughtful comment. With regard to Yb's role in the somatic piRNA pathway through condensate formation, studies provide experimental support for this function. We have therefore retained this description in the section explaining the OSC piRNA mechanism.

• Section 1.1.2. piRNA cluster transcription.

Before attempting any broader introduction into the topic, the first paragraph jumps into a highly specialized case in Drosophila without setting the frame. For the outsider this is not helpful and not understandable. This entire section lacks an attempt to synthesize a bigger picture. The extreme focus on Drosophila seems counter to the theme of the review. Also, section 1.1.5 seems to thematically fit much better together with section 1.1.2.

We appreciate the reviewer's thoughtful comments. As noted above, we have substantially reworked Section 1, including Sections 1.1.2 and 1.1.5. We now provide a concise overview of the piRNA pathway to establish a broader conceptual framework for readers who are less familiar with the field. We use *Drosophila* as a representative model to illustrate these principles and explicitly state that this focus is intentional and consistent with the overall scope of the review. Regarding pathways in other species, coverage remains necessarily limited due to space constraints; however, we now briefly highlight them at the end of the Introduction and in the section outlining the basic framework of piRNA-mediated TE repression (page 4). We believe these changes improve readability and sharpen the overall narrative of the review, thereby addressing the reviewer's concerns.

• Section 1.1.3.

This entire section reads a bit like a laundry list of findings from the literature without the attempt to synthesize these findings into a bigger picture or to bring disparate observations together into a clearer picture (expected from a review).

For both, Gtsf1 and Mael, the authors could consider updating their discussion about a common molecular function by incorporating recent findings that came out while this review was written (PMID: 40912244 and PMID: 40912245).

We appreciate the reviewer's thoughtful comment. In the revised manuscript, we have removed the subsections that previously focused on Gtsf1 and Mael, rather than retaining them as a list of individual examples. Thanks to the reviewer, we have incorporated the recently discovered proofreading mechanism that depends on piRISC binding to its target (page 7). We believe that these changes, together with the overall restructuring of Section 1, address the reviewer's concern about synthesis and improves the coherence of the review.

• *Section 1.2.2.*

This chapter seems detached from the rest as it is not discussed whether recombination-mediated loss of TE sequences (e.g. by LTR-LTR recombination) is an active, promoted process or a passive process.

We appreciate the reviewer's comment. As similar concerns were also raised by the other reviewers, we have removed this section from the revised manuscript. We believe this change improves the overall focus and coherence of the review.

• *Section 1.3.*

While an interesting overall topic, connecting evolutionary arguments to a limited level of understanding how the environment impacts TE biology and host defense biology, this chapter risks at being too simplistic in my opinion.

Indeed, as mentioned above, we no longer pretend to build any evolutionary theory. We just would like to collect some examples illustrating how (and hopefully why) some hosts (especially, *Drosophila*) tolerate their present-day TE inhabitants. We hope these focal changes address the reviewer's concerns.

• *Section 2.1.2.*

This is an overall interesting chapter that might further benefit from a more structured intro where it could be pointed out that most TEs must be expressed in germline cells as they cannot cross cell boundaries. And then explain two known scenarios how this paradigm can be altered. First, by evolving infectivity modules, second by exploiting the syncytial nature of germline development known from many organisms.

Thanks to this comment, we have added a useful introductory paragraph to this section (page 16).

• Section 2.1.3.

This is another chapter where I find it exceedingly challenging to make strong conclusions. Whether the rDNA units can be considered safe havens is a very difficult topic in my opinion. And to then combine this topic of safe heavens with insertion site preferences for the P-element is also a bit of a stretch. Surely, such promoter regions cannot be considered as safe havens, which is the title of the section.

We have already addressed this question (point 4), especially whether rDNA repeats may be considered as safe havens for TE insertion. Nevertheless, for the sake of clarity, we have changed the tittle of this section "*Preference for integration into specific insertional niches*" ("*genomic "safe havens*" has been preplaced by "*specific insertional niches*"). (page 17).

This section starts with a general paragraph describing how preferential insertion sites can be either harmless for the host (safe havens) or beneficial to TE. The latter case may be illustrated by P elements that preferentially insert close to replication origins to promote their amplification into the genome, bypassing their "cut and paste" strategy of integration. Since most of the replication origins are close to promoters, and following this comment, we also added the notion that recessive mutations likely caused by these insertions can still be tolerated by the host if the second allele of the targeted locus is functional.

• Section 2.1.4.

This section reads rather simplistic to me. Considering timing of integration, certain hard-wired features are important to keep in mind. For example, DNA transposons can only amplify in replicating cells due to their cut & paste mechanism. LTR retrotransposons on the other hand can be expressed at later stages of gametogenesis that lack mitotic activity. I suggest framing the arguments under the overall umbrella of molecular realities that different TEs or the host faces.

Our main point in the section 2.1.4 was to discuss timing of integration during germline development. We changed the tittle of this section accordingly. "*2.1.4. Timing of integration during germline development*" (page 18) and added an Introduction about the constraints that different classes of transposable elements (DNA transposons and LTR -retrotransposons) have to face (page 18).

Conclusion

In summary, while the manuscript covers an important and timely topic, it would benefit from a substantial revision before it can serve as a useful resource for the community. The authors should reframe the review around unifying conceptual themes, provide more balanced coverage across pathways and organisms, and synthesize findings into a coherent narrative. With these changes, the article has the potential to make a valuable contribution.

We sincerely appreciate the reviewers' valuable comments. In line with these suggestions, we have substantially revised the manuscript by restructuring it around a unified conceptual theme: host-TE coexistence. We have also reorganized the individual sections to better integrate previously published findings into a coherent narrative. We believe that these revisions now address the reviewers' concerns and strengthen the manuscript as a useful resource for the research community.

We regret that, given constraints on the overall length of the present review, we were unable to provide more balanced coverage across pathways and organisms. We fully acknowledge the importance of a more comprehensive review in the future.

Referee #2

Major points:

1）Since the authors state in section 1.1 and Figure 1 that TE repression is mediated by host small RNAs, including both piRNAs and endo-siRNAs, the manuscript would benefit from a brief discussion of endo-siRNAs alongside piRNAs. While it is reasonable to emphasize piRNAs under the theme of TE regulation, a minimal acknowledgment of endo-siRNAs would provide a more balanced perspective.

We appreciate the reviewer's helpful comment. In revising the manuscript, we have incorporated many of the valuable suggestions from all reviewers while keeping the overall length appropriate for a review article. Because of this, regarding endo-siRNAs, we limited ourselves to briefly introducing their presence and role in repressing TEs in the Introduction (page 2).

2）As this manuscript mainly focuses on the adaptive strategies ensuring peaceful coexistence between transposable elements and their hosts from the perspective of ecological frameworks adopted from community ecology, it would be beneficial to elaborate more explicitly on these concepts in the relevant sections.

We appreciate the reviewer's insightful comment. In the revised manuscript, we more explicitly develop the ecological framework adopted from community ecology and clarify how it applies to adaptive strategies that enable peaceful coexistence between TEs and hosts, with particular emphasis on *Drosophila* (particularly, Section 1). We believe that these revisions address the reviewer's concerns.

3）For the clarity to a broader readership, it would be helpful if the conserved piRNA pathway factors listed in Table 2 were annotated with their mammalian homologs. This would facilitate understanding for researchers from fields beyond insect biology.

We appreciate the reviewer's helpful suggestion. Because the revised manuscript now places greater emphasis on *Drosophila* mechanisms (particularly, Section 1), we have kept Table 2 without corresponding mammalian homologs. Regarding pathways in other species, coverage remains necessarily limited due to space constraints; however, we now briefly highlight them at the end of the Introduction and in the section outlining the basic framework of piRNA-mediated TE repression (page 4). We believe these changes improve readability and sharpen the overall narrative of the review, thereby addressing the reviewer's concerns.

4) In section 1.1.2, given that dual-strand clusters lack canonical promoter regions, the authors should better discuss the molecular signals that recruit the RDC complex to these loci.

We appreciate the reviewer's helpful suggestion. As recommended, we have added a description of the RDC complex in revised section "*Divergence between OSC and OGC piRNA pathways*" (page 9). "*OGCs: Germ cells primarily use dual-strand clusters, which are transcribed bidirectionally (Brennecke et al., 2007; Malone et al., 2009; Mohn et al., 2014). Uniquely, these loci lack canonical promoters; instead, transcription depends on a germline-specialized apparatus comprising Rhino (Rhi), an HP1-family protein; Moonshiner (Moon), a TFIIA-L homolog; Deadlock (Del) and Cutoff (Cuff), which together with Rhi form the RDC complex (Andersen et al., 2017; Chen et al., 2016; Klattenhoff et al., 2009; Mohn et al., 2014) (Table 2). This machinery, together with nuclear export factors for precursors, appears to be Drosophila-restricted, likely co-evolving with dual-strand clusters. Dual-strand clusters are maintained in a Rhi-dependent heterochromatic state, helping insulate cluster transcription from conventional gene expression. Dual-strand clusters may differ from each other by the specificity factors that are required for their targeting by the RDC complex (Baumgartner et al., 2022; Saito et al., 2025). OSCs do not express the factors required to activate these dual-strand clusters and therefore do not produce piRNAs from them (Table 2).*"

5）In section 1.1.2, the discussion is framed as addressing species divergence in piRNA cluster transcription; however, the description focuses exclusively on uni-strand and dual-strand clusters in Drosophila. This narrow focus does not fully reflect the stated theme of species divergence and could be broadened with examples from other taxa.

We appreciate the reviewer's thoughtful comments. In response to feedback from this reviewer and the other two reviewers, we have substantially restructured Section 1. To appreciate to what extent this section has been reframed, please refer to our response to the very first comment of reviewer #1. Because of this decision, the table of contents of the original Section 1 has been extensively revised and the term "species divergence" no longer appears in the titles of Section 1. We hope the reviewer will understand that this editorial choice also reflects the space constraints of the review.

6）Figure 4 provides a schematic overview of strategies employed by TEs for long-term propagation. To enhance the impact and scientific rigor, it would be beneficial to add some examples from published studies in the Figure.

As suggested by the reviewer, we have added the requested references in Figure 4 which is now Figure 5 legends as follows:

"Figure 5. Resolution of the genetic conflicts from the transposable element (TE) side.

Schematic representation of three different types of strategies employed by TEs. Each type of strategy involves different niches of expression and integration that TEs modulate in specific ways.

***Commensalism**: Spatial & temporal restriction: expression/integration (Carter et al., 2022; Cheung et al., 2016; Ghanim et al., 2020; Ke, 1997; Kirchner et al., 1995; Kojima et al., 2016; Lawlor et al., 2021; Sakashita et al., 2023; Sultana et al., 2017; Xie et al., 2001; Zou and Voytas, 1997). Spatial & temporal decoupling: expression/integration (Senti et al., 2025; Varoqui et al., 2025; Voichek et al., 2025; Wang et al., 2018).*

***Mutualism:** Genome structure: Integration (Casacuberta, 2017; Chabot et al., 2024; Kojima et al., 2016; Nelson et al., 2023). Development & immunity: Expression (Brégnard et al., 2016; Chang et al., 2025; Grow et al., 2015; Jachowicz et al., 2017; Larouche et al., 2024; M'Angale et*

al., 2025; Mathavarajah and Dellaire, 2024; Moore et al., 2021; Percharde et al., 2018; Sakashita et al., 2023; Wang et al., 2022).

***Reducing TE-TE competition**: Niche partitioning: Expression/integration (Klumpe et al., 2025; Senti et al., 2025; Son et al., 2025; Varoqui et al., 2025; Ye et al., 2005).”*

Minor points:

1）The factor Armi should be included in Table 2.

We have added Armi in revised Table 2. Thank you.

2）In section 1.2.1, the statement "High expression of KRAB-ZFPs is frequently observed in early embryonic cells and the germline, developmental windows in which epigenetic reprogramming renders the genome particularly vulnerable to TE activation" requires appropriate references.

We appreciate the reviewer’s comment. In the revised manuscript, we now confine our discussion of KRAB-ZFPs to a brief mention in the Introduction (page 2), where we note their presence in mammals/tetrapods and highlight how they differ from the piRNA pathway. In this context, we have added appropriate references to support these statements. We believe this revision addresses the reviewer’s concern.

3）In section 2.1.1, the nomenclature of "MERVL" and "HERV-K" should be standardized for consistency.

As suggested by the reviewer, we have standardized the nomenclature.

4）In section 2.2.3, the claim that "Since HERV-K activation is OCT4-dependent and occurs under genome-wide hypomethylation, it has been proposed that primordial germ cells (PGCs) may similarly harness HERV-K to fend off viral threats." requires specific supporting citations.

We added a reference (Grow et al., 2015) (page 21).

5）In section 2.2.3, the second paragraph could be merged with the first, as both address the influence of transposable elements on innate immune responses.

We have done the modification as suggested.

Referee #3

I enjoyed reading this review but have a few important comments about the content, mostly regarding the first half of the review on the evolution of TE-controlling factors. Crucially, the topic of the review is TE/genome coexistence. Much of the discussion in the first half is about the evolution of piRNAs, focussing on some specific proteins. It is not made explicit in the article how this actually relates to the topic. Whilst of course it is plausible that the reason that piRNAs evolve fast is the TE/host arrms race, this is still a conjecture. Plenty of other molecular pathways evolve fast; moreover the 'fast evolution' idea is quite difficult to actually quantify; how fast would you expect a pathway to evolve? Do piRNA factors evolve faster than the norm (probably, but this hasn't really been directly demonstrated very often)? Even if they do, is this because of their function or simply because of the type of proteins that regulate the piRNA pathway (which might simply be robust to mutations so can accumulate diversity rapidly)? And finally, it's important to note that piRNAs in many arthopods have an antiviral role, rather than just TEs, which might also lead to fast evolution. These might seem far-fetched but they are important caveats to the argument. It's important that the authors discuss this before listing their examples. Importantly none of the examples actually directly link to TEs, so it's vital that the link is made clear so that the information is relevant to the overall questions.

We appreciate the reviewer's thoughtful comments. In response to feedback from this reviewer and the other two reviewers, we have substantially restructured Section 1, now entitled "*Host-centered strategies for host–TE coexistence*."

In the revised version, Section 1 begins by outlining the basic framework of the piRNA pathway. We then provide an overview of the *Drosophila* piRNA pathway, followed by a discussion of cell type-specific diversification of piRNAs in two ovarian cell types, OSCs and OGCs, addressing both their shared framework and distinct features. Although these two cell types coexist within the same ovarian tissue, their piRNA mechanisms differ, and we now describe this contrast explicitly as a unifying example of host adaptation to distinct TE challenges.

Our rationale is as follows: TEs integrate into host genomes, and their transposition can damage host genes. Consequently, hosts repress TEs via the piRNA pathway. Paradoxically, hosts that fail to restrain TEs lose reproductive capacity owing to excessive transposition, thereby eliminating the genomic niche that sustains TEs. Thus, the piRNA pathway functions not only as a defense system against TEs but also as an essential mechanism enabling stable host–TE coexistence. We therefore consider it reasonable that the host-focused section begins with piRNA-mediated TE silencing as its central framework. Because of this restructuring, we have removed the rapid evolution of piRNA factors. However, since this topic remains important, we plan to revisit it in future reviews if the opportunity arises.

We have not responded to each of the reviewer’s questions individually (for which we sincerely apologize), but we hope that this reviewer, as well as the other two reviewers, will find the revised structure of Section 1 more clearly framed around overarching themes and better aligned with the main topic of host–TE coexistence.

Regarding the antiviral effects of piRNAs, we have added the following sentence to the revised manuscript (page 4-5), “*Moreover, in some organisms, such as mosquitoes, piRNAs are not restricted to the germline and have well-established antiviral roles (Haase et al., 2024; Miesen et al., 2016; Varjak et al., 2018).*”

Another crucial point that would be worth adding to this review is the interesting point that piRNAs can act as a 'double-edged sword' when it comes to TE control. piRNAs suppress TEs, but paradoxically this enables the genome to tolerate more TE copies. It's possible that loss of the piRNA pathway might actually be ultimately beneficial in meaning that purifying selection eliminates TEs (thus enabling ultimately a higher fitness to be reached, albeit traversing through a region of the fitness landscape with reduced fitness due to the loss of the piRNA-mediated repression). This model was first described computationally by Lu and Clark (https://genome.cshlp.org/content/20/2/212). There is also some empirical evidence for it (10.15252/msb.20209600).

We appreciate the reviewer’s thoughtful comment. Regarding the intriguing idea that piRNAs may act as a double-edged sword in TE regulation, we have incorporated this perspective into the revised paragraph 1.3 untitled *“Host tolerance of TEs: biological relevance and underlying strategies* “(page 12).

“*However, TE repression may also participate in the success of TE invasions (the double-edged sword paradox) by attenuating the deleterious effects of mobilization and therefore reducing the risk of TE elimination by selection (Lu and Clark, 2010). Generalization of this paradoxical observation to a wide survey of metazoans, confirmed that species with piRNAs do tend to contain a larger number of TE families, each of which having fewer copies in their genome (Schneider et al., 2021). Since TEs can, in the long term, fuel host regulatory innovations (Bourque et al., 2018; Fueyo et al., 2022), such “tolerant” TE-rich hosts are more likely to have adapted to new environments. That is probably why TE repression has been conserved in present-day host-TE interactions, although not always at optimal levels: permitting limited transposition would preserve essential functions while enabling long-term adaptation. Accordingly, piRNAs operate less as a blunt instrument of resistance than as gatekeepers of managed coexistence, tolerating controlled TE activity under realistic constraints while*

safeguarding genome integrity. In this tolerance model, particularly under stress, hosts would exploit manageable transposition rates and the resulting genetic diversity instead of striving for absolute repression.”

We believe it adds further depth to the discussion.

Specific comments

Section 1

The examples here are drawn heavily from Drosophila, with some information from mammals; it is fine but it's worth specifying upfront that there are large groups of organisms that are not considered- e.g. other arthropods (see also another comment below), nematodes, for which quite a lot of mechanistic information about the piRNA pathway and its relationship to TEs is known.

We appreciate the reviewer’s comment and fully share these concerns. In response, we have added the following sentences to the revised text (page 3): “*The piRNA pathway is highly conserved across diverse animal lineages, and knowledge of how hosts and TEs interact through this mechanism has grown substantially. In this review, however, we focus primarily on Drosophila and do not cover in detail other taxa such as nematodes and non-drosophilid arthropods. Readers are referred to several excellent reviews for broader, comparative perspectives on piRNA pathways across animals (Juliano et al., 2011; Weick and Miska, 2014)*.”

1.1.1 Only 2 egs are chosen here and this isn't really explained properly- could the specific relevance of these be expanded on slightly? Is it just because they are very recent? Perhaps it's better if this is rewritten to point out some general aspects of piRNA pathway divergence, using these two as specific examples, rather than just picking them, which implies that each protein evolves idiosyncratically and the general relevance to TE silencing evolution is not then clear.

We appreciate the reviewer’s comments. To address the comments and concerns raised by this reviewer and the other two reviewers, we have substantially restructured Section 1. In parallel, the content of the original Section 1.1.1 has been extensively revised. We believe that these changes adequately address the reviewer’s concerns.

1.1.2 Again this needs to be more clearly linked to TEs, which aren't mentioned at all in this section.

We appreciate the reviewer’s comments. As described in our response to this reviewer’s first comment, we have substantially restructured Section 1 and revised the material that previously

appeared in Section 1.1.2 so that its relevance to TE biology and host–TE coexistence is now made explicit. We would be grateful if the reviewer could refer to our response to the first comment for further details.

1.1.3 This is not really relevant to TE silencing, unless the point is that the fast evolution is somehow facilitated by the fact that piRNA pathways can incorporate other proteins easily thus enabling them to evolve fast; if this is the argument then it should be stated explicitly.

We appreciate the reviewer's comments. As outlined in our response to this reviewer's first comment, we have substantially restructured Section 1 and have removed the original Section 1.1.3 as a stand-alone discussion of rapid piRNA pathway evolution. In the revised manuscript, we no longer present this topic independently, so as to keep the focus on mechanisms that are more directly linked to TE silencing and host–TE coexistence. We fully agree that the rapid evolution of the piRNA pathway is an intriguing issue, and we hope to prepare a dedicated review on this subject in the future.

1.1.4 The link to TEs is again absent here.

We appreciate the reviewer's comments. As described in our response to this reviewer's first comment, we have substantially restructured Section 1 and revised the material that previously appeared in Section 1.1.4 so that its relevance to TE biology and host–TE coexistence is now made explicit. We would be grateful if the reviewer could refer to our response to the first comment for further details.

1.1.5 This doesn't seem relevant to the review but I think that it could be made much more on-topic by including a discussion of a key issue in piRNA evolution which is not touched on: why it has become germline-restricted so often in evolution. Taking the examples of arthropods, the ancestral arthropod piRNA pathway was almost certainly somatic as well as germline. However, Drosophila evolved germline-restricted piRNAs, which of course links directly to the transcriptional control (gain of sites for a germline-restricted TF for piRNA factors). It's very likely that the somatic gonad piRNA pathway was then a secondary innovation after germline restriction evolved, to resist a new TE family. This is a really good example of TE/host arms race, but it still begs the question of why piRNAs ever become germline restricted. Perhaps there is a cost associated with TE silencing that is not 'worth it' in the soma (maybe targeting other genes by mistake)? In any case, this is an important discussion to have at this point in the review.

We appreciate the reviewer's thoughtful comments. We fully agree that understanding why piRNA pathways have so often become germline-restricted during evolution is an important and fascinating issue. However, in response to comments from this reviewer and the other two reviewers, we have substantially restructured Section 1 to focus more tightly on host–TE coexistence (please refer to our response to this reviewer's initial comment). Within this revised framework, there is now limited scope to develop a detailed discussion of the evolutionary causes of germline restriction, and we have therefore chosen to omit this topic from the present review. We nonetheless recognize its significance and regard it as a key subject for a future, dedicated review on piRNA evolution.

1.1.6. This is interesting, but seems out of place given the preceding sections which are about evolution of piRNA pathways, whereas this is a distinct piRNA function. Could the authors do a bit better at linking this in with the previous sections, perhaps by emphasizing that it is an example of how the entire mechanism of the piRNA pathway can evolve in completely distinct directions in response to the unusual biology of the ciliates.

We appreciate the reviewer's comments. In the revised manuscript, we now provide a more comprehensive treatment of ciliate biology in" *1.2) Programmed DNA elimination as an extreme mode of host–TE coexistence" (page 11)*. There, we explicitly frame ciliates as an example of how piRNA-related pathways can diverge in a distinct direction in response to their unusual biology, thereby linking this topic more clearly to the overall theme of piRNA pathway evolution and host–TE coexistence. We hope that the amendments we have made satisfactorily address this reviewer's concerns.

1.2.2 This section is quite misleading at the moment. All the other points discussed are 'active' functions of control mechanisms, but here the argument is about mutations that presumably occur randomly but can lead to loss of a TE, which may then be acted on by selection and go to fixation. This isn't really on topic and should be removed, especially as the way it's currently written implies that there's some kind of active mutagenesis going on here akin to the ciliate programmed genome elimination, which as far as we know is not the case here.

We appreciate the reviewer's comments and fully agree with this assessment. Accordingly, former Section 1.2.2 has been removed from the revised manuscript.

1.3 This would be the place to incorporate the 'double edged sword' idea, maybe touching on the fact that some species have indeed lost piRNAs altogether (many nematodes, parasitic flatworms, dust mites).

We appreciate the reviewer’s insightful suggestion. In line with this comment, we have now incorporated the “double-edged sword” concept into a new subsection entitled “*1.3) Host tolerance of TEs: biological relevance and underlying strategies”* (page 12). In this section, we discuss how stringent TE silencing can impose fitness costs on the host and briefly highlight the apparent paradox that lineages in which the piRNA pathway has been lost (e.g., certain nematodes, parasitic flatworms, and dust mites) tend to be TE poor. We believe that these additions enrich Section 1.3 and add further depth to the discussion.

2.2 I may have missed it but I didn't see anything here about the famous Rag tranposase in vertebrate immune system, which, although hackneyed, is worth including here (I think many readers will be expecting it).

In the section 2.2.1, we aim to describe how active TEs coexist with their host while contributing to its genomic structure. We agree with the reviewer that Rag is a textbook example for describing the impact of TEs on genome structure but (and it is the reason why we did not include into this section) Rag transposase results from TE remnants instead of still active TEs.

Dear Dr. Siomi,

Thank you for submitting your manuscript for consideration by the EMBO Journal. It has now been seen by two of the original referees whose comments are enclosed. As you will see, both referees find the review proposal improved and are in favor of publication, pending satisfactory revision to address the remaining minor concerns of referee #1.
In addition there are a small number of editorial points that we would ask you to already incorporate into the revised manuscript:

(1) Please rename the Conflict of Interest section into "Disclosure and Competing Interests Statement", in accordance with our updated Guide to Authors (https://link.springer.com/partners/embo-press/editorial-policies#Competing%20interest%20disclosures)

(2) Please provide all figure files in a tiff, eps or pdf format.

(3) Please adjust the format of the reference list and of the in-text citations according to EMBO Journal format (alphabetical order, author name et al + year.../up to 10 author names in the reference list before et al / please refer to our Guide to Authors for additional information on EMBO J reference format)

Thank you for the opportunity to consider your work for publication. I look forward to your revision.

Yours sincerely,

Cornelius Schneider, PhD
Editor
The EMBO Journal
c.schneider@embojournal.org

Instructions for preparing your revised manuscript:

Read our guidance for manuscript revisions and related editorial policies: https://link.springer.com/journal/44318/submission-guidelines#cms-Revised-submissions

----

Referee #1:

The revised version of the review "Host-transposable element coexistence: a matter of resistance, tolerance and trade-off" is overall improved, and I appreciate the effort that the authors have made to make it more complete and more accessible while sharpening the focus.

All in all, this review does contribute adequately to the current state of the art in the field. Some aspects nevertheless do require revisions as detailed below.

- Line 36: the listed defense strategies are exclusive to animals. It therefore would make sense to explicitly restrict the scope of the review to animals.

- I am not convinced that the nomenclature of OSC and OGC is useful. OSCs is an established term in the field for a cultured cell line derived from ovarian somatic stem cells. This name is therefore not suitable to describe the diverse cell types in the ovarian soma. Similarly, the name OGC for the different germline cell types is in my opinion not helpful as it is a new name bearing no real justification. In my opinion, abbreviations are rarely helpful as they make the text less accessible. I see no reason to abandon the usage of ovarian soma and ovarian germline throughout the review.

- Line 129
"Throughout most of Drosophila oogenesis, OGCs are encapsulated and protected by OSCs. This tissue architecture suggests that TEs first invade the OSC genome"
What is the basis for this statement?

- Line 135
"In OSCs, TE control bypasses piRNA-dependent PTGS, which may represent a cost-saving strategy."
I am not sure whether I understand the meaning of this sentence.

- Line 138:
"OGCs must contend not only with TEs that have been horizontally transmitted from OSCs"
Is this the right use of the term 'horizontal transmission'??

- Line 288
"These unicellular eukaryotes contain two nuclear compartments with a shared cytoplasm: a transcriptionally silent germline micronucleus (MIC) and a transcriptionally active somatic
macronucleus (MAC)."
These are rather two distinct nuclei, not nuclear compartments.

- On horizontal transfer of TEs: The recent rather rich evidence for horizontal transfer among TEs in Drosophilids by Robert Kofler might be worth mentioning/citing.

- Citations: I appreciate the challenge when it comes to citing the literature in a consistent, fair, but mostly useful (for the reader) manner. Nevertheless, this manuscript does require quite some effort in making sure that the citations are adequate. Here are some examples:
o Overall, I suggest improving the citations by carefully checking whether individual statements are rooted in the right citations. The biology of uni/dual strand clusters for example refers to Brennecke et al 2007, but there are more important citations in later papers in my opinion.
o For several proteins in the piRNA pathway, it seems that the authors tend to cite later papers but not the original papers that have first linked them to transposon control or piRNA biology.
o Line 169:
PICTS/PPNP/SFiNX/Pandas complex: one citation is missing.
o Citations in the ciliate chapter are on the thin side.
o Line 372: citations for the P-element part are almost entirely missing. There is for example a great review on the P-element by Teixera, Rio and colleagues.
o Line 402: the authors describe somatic expression of endogenous retroviruses. This chapter neglects the early, pioneering studies that discovered this mode of transposition (work from Vaury and Bucheton/Pelisson).
o Line 533:
entire paragraph without citations

- Figure 4:
o one factor in silencing is mis-spelled: Cut up, not Cup up.
o For the section on dual-strand cluster biology, Nxt1 is mentioned, but Nxf3 (more important) is not.

- Nxf3 is missing in Table 2.

Referee #3:

I am happy that the authors have responded to all my queries adequately and that the manuscript should be published.

Responses to the Referee #1's comments:

Referee #1:

The revised version of the review "Host-transposable element coexistence: a matter of resistance, tolerance and trade-off" is overall improved, and I appreciate the effort that the authors have made to make it more complete and more accessible while sharpening the focus. All in all, this review does contribute adequately to the current state of the art in the field.

We thank the referee for his/her positive comments.

Some aspects nevertheless do require revisions as detailed below.

- Line 36: the listed defense strategies are exclusive to animals. It therefore would make sense to explicitly restrict the scope of the review to animals.

We thank the reviewer for this comment. We have removed from the Introduction the sentence stating, "*Indeed, the highly reduced genomes of apicomplexan parasites are completely devoid of TEs, a state thought to have arisen through such processes (Kissinger and DeBeBarry, 2011)*." Please see around lines 50-51 of the revised text (page 2). We believe this revision addresses the reviewer's concern. If not, we would appreciate further guidance on what aspect remains insufficient or unclear.

- I am not convinced that the nomenclature of OSC and OGC is useful. OSCs is an established term in the field for a cultured cell line derived from ovarian somatic stem cells. This name is therefore not suitable to describe the diverse cell types in the ovarian soma. Similarly, the name OGC for the different germline cell types is in my opinion not helpful as it is a new name bearing no real justification. In my opinion, abbreviations are rarely helpful as they make the text less accessible. I see no reason to abandon the usage of ovarian soma and ovarian germline throughout the review.

We thank the reviewer for this comment. Throughout the manuscript, we replaced "OSCs" with "ovarian somatic cells" and "OGCs" with "ovarian germ cells". Where a direct substitution would have resulted in awkward phrasing, we adjusted the wording accordingly (e.g., line 140, "OSCs and OGCs" were revised to "ovarian somatic and germ cells". We also add a note stating (page 6), "*Studies of the piRNA pathway in ovarian somatic cells have been advanced by the use of cultured OSC lines (Saito et al., 2016). We note, however, that it is difficult—and often impractical—to draw a strict boundary between insights gained from these in vitro systems and those obtained from analyses of somatic cells within the ovary*." We believe this revision addresses the reviewer's

concern.

- Line 129

"Throughout most of Drosophila oogenesis, OGCs are encapsulated and protected by OSCs. This tissue architecture suggests that TEs first invade the OSC genome"

What is the basis for this statement?

We thank the reviewer for this comment. We have now cited two relevant papers (page 5), which we believe addresses the reviewer's concern.

- Line 135

"In OSCs, TE control bypasses piRNA-dependent PTGS, which may represent a cost-saving strategy." I am not sure whether I understand the meaning of this sentence.

We apologize for the lack of clarity. Our intention was to convey that, in OSCs, TE repression primarily relies on Piwi-mediated nuclear silencing (TGS), whereas canonical piRNA-dependent cytoplasmic PTGS via the ping–pong pathway is largely absent. We have revised the sentence to make this point explicit. The revised sentences now read: "*In ovarian somatic cells, TE repression relies predominantly on Piwi-mediated nuclear silencing (TGS), whereas Aub/Ago3-dependent cytoplasmic PTGS (ping–pong amplification) is limited or absent, potentially reducing the energetic burden of sustained cytoplasmic cleavage and amplification. Breakdown of this surveillance could, in turn, create an opportunity for TEs to breach the somatic-germline barrier and invade ovarian germ cells*."

- Line 138:

"OGCs must contend not only with TEs that have been horizontally transmitted from OSCs". Is this the right use of the term 'horizontal transmission'?

We thank the reviewer for this comment. The revised sentences now read: "*Ovarian germ cells must contend not only with TEs originating in somatic cells"*

- Line 288

"These unicellular eukaryotes contain two nuclear compartments with a shared cytoplasm: a transcriptionally silent germline micronucleus (MIC) and a transcriptionally active somatic macronucleus (MAC)." These are rather two distinct nuclei, not nuclear compartments.

We thank the reviewer for this comment. We have revised the corresponding sentence to "*These unicellular eukaryotes contain two nuclei with a shared cytoplasm: a*

transcriptionally silent germline micronucleus (MIC) and a transcriptionally active somatic macronucleus (MAC)."

- On horizontal transfer of TEs: The recent rather rich evidence for horizontal transfer among TEs in Drosophilids by Robert Kofler might be worth mentioning/citing.

We have now cited the paper, Pianezza et al., 2025, on page 22.

- Citations: I appreciate the challenge when it comes to citing the literature in a consistent, fair, but mostly useful (for the reader) manner. Nevertheless, this manuscript does require quite some effort in making sure that the citations are adequate. Here are some examples:

- Overall, I suggest improving the citations by carefully checking whether individual statements are rooted in the right citations. The biology of uni/dual strand clusters for example refers to Brennecke et al 2007, but there are more important citations in later papers in my opinion.

We have now cited the paper, Mohn et al., 2014, on page 5.

- For several proteins in the piRNA pathway, it seems that the authors tend to cite later papers but not the original papers that have first linked them to transposon control or piRNA biology.

We have now cited the following papers in the piRNA section.

Page 7, L156 : Qi et al., 2011 (10.1074/jbc.M110.193888), Saito et al., 2010 (10.1101/gad.1989510), Szakmary et al., 2009 (10.1083/jcb.200903034)

Page 7, L159 : Saito et al., 2010 (10.1101/gad.1989510)

Page 7, L165 : Handler et al., 2013 (10.1016/j.molcel.2013.04.031)

Page 7, L170: Yashiro et al., 2018 (10.1016/j.celrep.2018.05.051)

Page 7, L174: Fabry et al., 2019 (10.7554/eLife.47999)

Page 8, L189 : Feltzin et al., 2015 (10.1111/acel.12323), Wang et al., 2016 (10.1242/dev.128116)

Page 9, L219 : ElMaghraby et al., 2019 (10.1016/j.cell.2019.07.007), Kneuss et al., 2019 (10.1101/gad.328690.119)

Page 10 L251 : Ishizu et al., 2019 (10.1016/j.celrep.2019.04.029), Olivieri et al., 2012 (10.1016/j.molcel.2012.07.021), Preall et al., 2012 (10.1261/rna.034405.112), Saito et al., 2010 (10.1101/gad.1989510)

Page 11, L272 : Anand and Kai, 2012 (10.1038/emboj.2011.449), Lin et al., 2023 (10.1083/jcb.202303125), Nishida et al., 2009 (10.1038/emboj.2009.365), Ryazansky et al., 2016 (10.1016/j.ejcb.2016.06.001)

- Line 169:

PICTS/PPNP/SFiNX/Pandas complex: one citation is missing.

We have now cited the paper, Fabry et al., 2019, on page 7.

- Citations in the ciliate chapter are on the thin side.

We have added citations in the revised manuscript (page 12).

- Line 372:

citations for the P-element part are almost entirely missing. There is for example a great review on the P-element by Teixera, Rio and colleagues.

We have added citations in the revised manuscript (page 15).

- Line 402:

the authors describe somatic expression of endogenous retroviruses. This chapter neglects the early, pioneering studies that discovered this mode of transposition (work from Vaury and Bucheton/Pelisson).

We thank the reviewer for pointing this out. In the ovaries, all infectious ERVs, including ZAM, are specifically transcribed in the somatic follicle cells (Senti et al., 2025), which surround the oocyte that they may infect. Related references are Brasset et al., 2006 (doi:10.1186/1742-4690-3-25), Lecher et al., 1997 (doi:10.1099/0022-1317-78-9-2379), and Pelisson et al., 1994 (doi:10.1002/j.1460-2075.1994.tb06760).

- Line 533:

entire paragraph without citations

We thank the reviewer for this. From another point of view, while these innate immune pathways provide a first line of defense against exogenous viral infection, in the germline the piRNA pathway triggers some adaptative immunity against TEs *per se*. Indeed, TE integration into piRNA clusters imposes a feedback loop: TEs self-limit their own proliferation by donating sequences into the piRNA network [Malone et al., 2009 (doi:10.1016/j.cell.2009.01.045), Czech et al., 2018 (https://doi.org/10.1146/annurev-genet- 120417031441), and Ozata et al., 2019 (https://doi.org/10.1038/ s41576-018-0073-3)]. Because piRNA clusters are typically heterochromatic and safe for insertions, TEs often “accidentally” land there; however, once integrated, they contribute to their

own silencing, stabilizing the TE–host equilibrium [Bergmann et al., 2006 (doi:10.1186/gb-2006-7-11-r112) and Zanni et al., 2013 (doi: 10.1073/pnas.1313677110)].

- Figure 4:

・one factor in silencing is mis-spelled: Cut up, not Cup up.

・For the section on dual-strand cluster biology, Nxt1 is mentioned, but Nxf3 (more important) is not.

・Nxf3 is missing in Table 2.

We fixed all. Thank you.

Dear Dr. Siomi,

I am pleased to inform you that your manuscript has been accepted for publication in the EMBO Journal.

Your manuscript will be processed for publication by EMBO Press. It will be copy edited and you will receive page proofs prior to publication. Please note that you will be contacted by Springer Nature Author Services to complete licensing and payment information.

You may qualify for financial assistance for your publication charges - either via a Springer Nature fully open access agreement or an EMBO initiative. Check your eligibility: https://link.springer.com/journal/44318/how-to-publish-with-us

Should you be planning a Press Release on your article, please get in contact with embo_production@springernature.com as early as possible in order to coordinate publication and release dates.

If you have any questions, please do not hesitate to contact the Editorial Office. Thank you for your contribution to The EMBO Journal.

Yours sincerely,

Cornelius Schneider, PhD
Editor
The EMBO Journal
c.schneider@embojournal.org

*****
Please note that it is The EMBO Journal policy for the transcript of the editorial process (containing referee reports and your response letters) to be published as an online supplement to each paper. If you should prefer removal of any referee-only figures included in the point-by-point response(s), e.g. because they may still be used for future publication or because they have been reproduced from published work by others, please do let us know immediately via response email.
More information is available here: https://link.springer.com/partners/embo-press/editorial-policies#Peer%20review
